# Impact of the macrocyclic structure and dynamic solvent effect on the reactivity of a localised singlet diradicaloid with π-single bonding character†

**DOI:** 10.1039/d0sc05311b

**Published:** 2020-11-10

**Authors:** Zhe Wang, Rikuo Akisaka, Sohshi Yabumoto, Tatsuo Nakagawa, Sayaka Hatano, Manabu Abe

**Affiliations:** Department of Chemistry, Graduate School of Science, Hiroshima University 1-3-1 Kagamiyama, Higashi-Hiroshima Hiroshima 739-8526 Japan mabe@hiroshima-u.ac.jp; Unisoku Co., Ltd. 2-4-3 Kasugano, Hirakata Osaka 573-0131 Japan; Hiroshima University Research Centre for Photo-Drug-Delivery-Systems (HiU-P-DDS), Hiroshima University 1-3-1 Kagamiyama, Higashi-Hiroshima Hiroshima 739-8526 Japan

## Abstract

Localised singlet diradicals are key intermediates in bond homolysis processes. Generally, these highly reactive species undergo radical–radical coupling reaction immediately after their generation. Therefore, their short-lived character hampers experimental investigations of their nature. In this study, we implemented the new concept of “stretch effect” to access a kinetically stabilised singlet diradicaloid. To this end, a macrocyclic structure was computationally designed to enable the experimental examination of a singlet diradicaloid with π-single bonding character. The kinetically stabilised diradicaloid exhibited a low carbon–carbon coupling reaction rate of 6.4 × 10^3^ s^−1^ (155.9 μs), approximately 11 and 1000 times slower than those of the first generation of macrocyclic system (7.0 × 10^4^ s^−1^, 14.2 μs) and the parent system lacking the macrocycle (5 × 10^6^ s^−1^, 200 ns) at 293 K in benzene, respectively. In addition, a significant dynamic solvent effect was observed for the first time in intramolecular radical–radical coupling reactions in viscous solvents such as glycerin triacetate. This theoretical and experimental study demonstrates that the stretch effect and solvent viscosity play important roles in retarding the σ-bond formation process, thus enabling a thorough examination of the nature of the singlet diradicaloid and paving the way toward a deeper understanding of reactive intermediates.

## Abstract 2

An extremely long-lived localised singlet diradical with π-single bonding character is found in a macrocyclic structure that retards the radical–radical coupling reaction by the “stretch and solvent-dynamic effects”.

## Graphical abstract

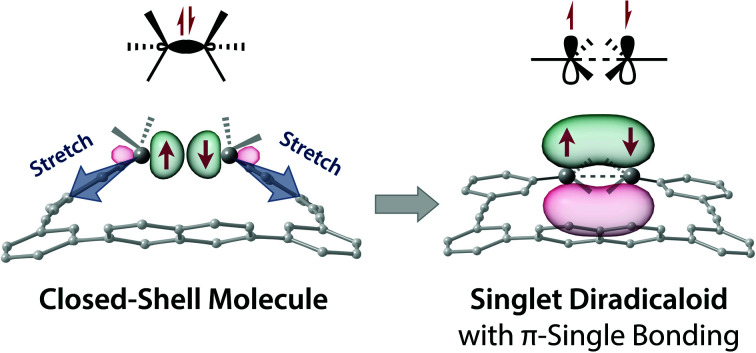

## Introduction

The insight into fundamental processes and innate reactivity often triggers innovation. For example, the isolation and fundamental understanding of carbenes enable their practical application in chemical synthesis.^1–13^ Although much effort was devoted to proving the existence and exploring the role of singlet diradicals, key intermediates in homolytic bond cleavage,^14–21^ in chemical reactions over the last century, important aspects remain unexplored. Diradicaloids, singlet diradicals with closed-shell character,^22^ have attracted significant attention not only because of their reactivity, but also due to their unique properties, which stem from the borderline character between open- and closed-shell molecules. In this context, non-linear optical properties^23^ and the singlet-fission phenomenon^24^ are typical examples. However, the reactivity of open-shell species renders the isolation of air-stable diradicaloids at room temperature challenging. In the last decade, several delocalised singlet diradicaloids, such as Tchitchibabin-type diradicals, have been isolated by exploiting steric and π-conjugation effects.^25–44^

**Scheme 1:**
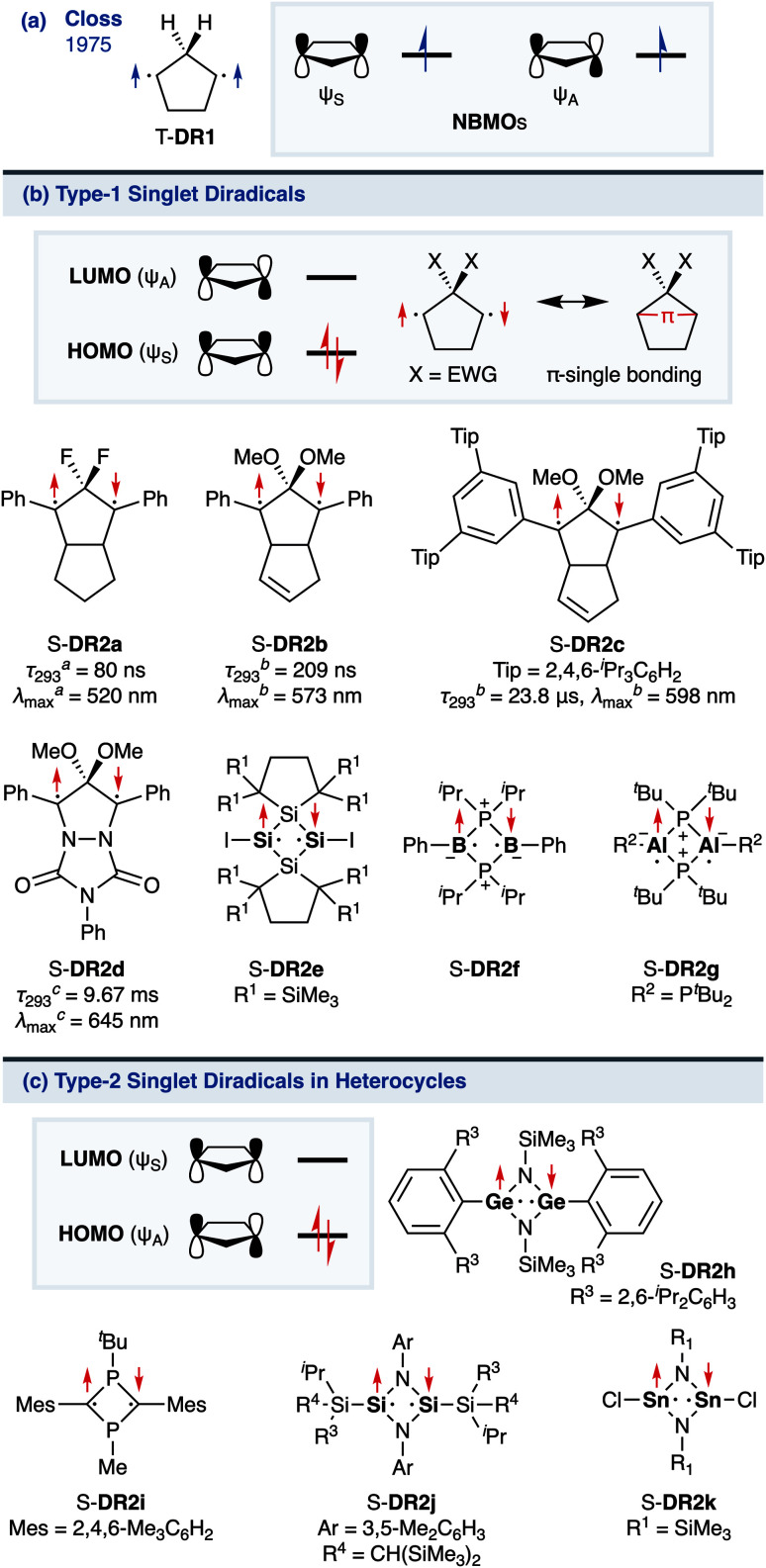
(a) Pioneering work of Closs; (b) Type-1 and (c) Type-2 localised singlet diradicaloids in (hetero)cyclobutane and -pentane systems. The lifetime *τ*_293_ and maximum absorption wavelength *λ*_max_ were determined in ^*a*^*n*-pentane, ^*b*^benzene, or ^*c*^toluene.

Regarding localised diradicals, highly reactive species that undergo fast radical–radical coupling reaction, the low-temperature matrix isolation of diradical T-DR1 was first achieved in 1975 (Scheme 1a). The isolation allowed a detailed investigation of the ground-state spin multiplicity and reactivity of this species, resulting in the elucidation of its triplet ground state and its heavy-atom tunnelling reaction.^45–47^ Furthermore, carbon–carbon singlet diradical S-DR2a (*τ*_293_ = 80 ns in *n*-pentane), in which electron-withdrawing groups (EWGs) lower the energy of the singlet state with regard to that of the triplet state, was first detected in 1998,^48^ whereas the longer-lived S-DR2b (*τ*_293_ = 209 ns in benzene) featuring flexible alkoxy groups has been studied in our laboratory.^49–52^

Localised singlet diradicaloids can be classified as Type-1 or Type-2 according to their most stable electronic configuration, which in turn depends on the relative HOMO and LUMO energy levels (Scheme 1).^53,54^ Hence, the π-single bonding character (C–π–C) characterises Type-1 molecules because the bonding orbital ψ_S_ is HOMO.^55^ Recently, long-lived singlet diradicaloids S-DR2c (*τ*_293_ = 23.8 μs in benzene) and S-DR2d (*τ*_293_ = 9.67 ms in toluene), featuring the bulky-substituent and nitrogen-atom effects, respectively, were observed at 293 K.^56,57^ Additionally, several heavy-atom analogues S-DR2e–k, including Type-2 singlet diradicaloids, have been isolated (Scheme 1).^58–64^ Very recently, five- and four-membered cyclic silicon analogues of π-single bonded species were reported.^58,65,66^

**Scheme 2:**
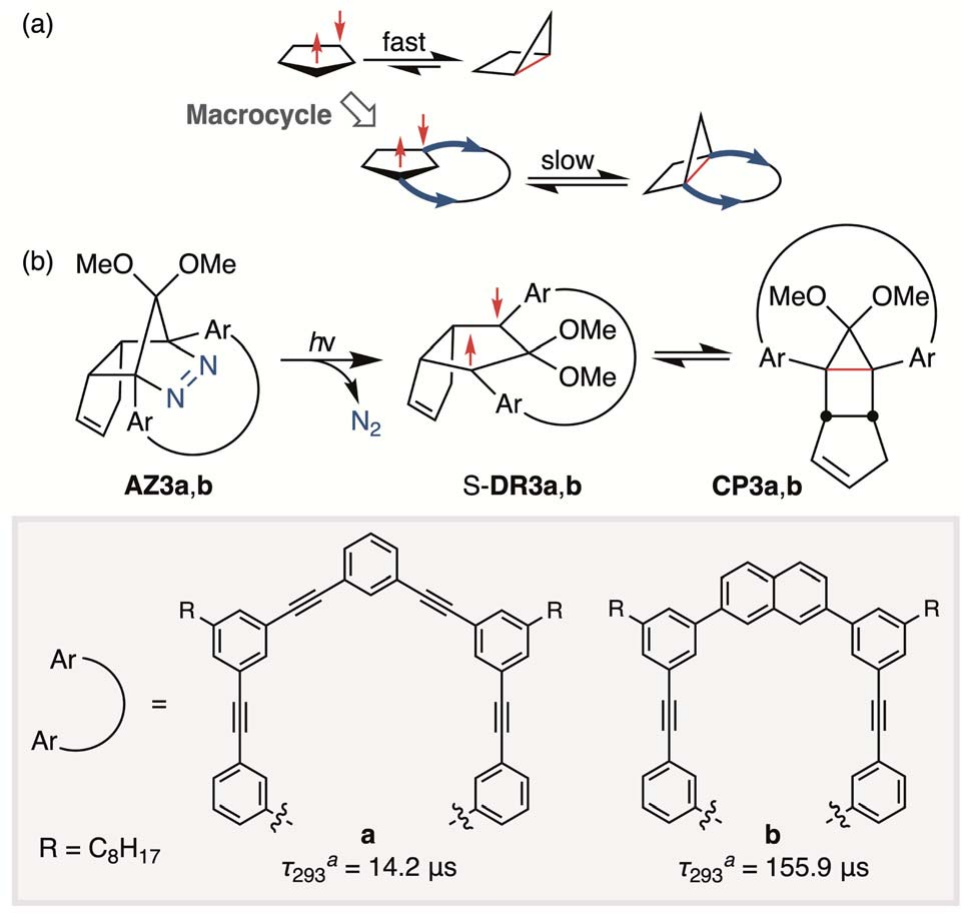
(a) Stretch effect induced by the macrocycle. (b) Localised diradicaloids investigated in this study. ^*a*^Lifetime values *τ*_293_ were determined in benzene.

In 2012, macrocyclic structures were designed to kinetically stabilise carbon–carbon singlet diradicaloids (Scheme 2).^67^ In these scaffolds, structural rigidity precludes the σ bond formation between the radical centres, a phenomenon termed “stretch effect” (Scheme 2a). Recently, this effect was studied using macrocyclic singlet diradicaloid S-DR3a. The moderate increase in the lifetime of S-DR3a to *τ*_293_ = 14.2 μs in benzene (Scheme 2b) indicated that the construction of macrocyclic structures is a useful strategy to extend the lifetime of singlet diradicaloids and enable more detailed investigations.^68^ This finding prompted us to devise a new fine-tuned macrocyclic structure. In this study, singlet diradicaloid S-DR3b featuring a naphthalene-containing macrocyclic system was designed and examined by computational and experimental studies. In addition, its reactivity toward radical–radical coupling, the features of this reaction, and the properties of its products were investigated in detail.

## Results and discussion

### Computations

**Fig. 1:**
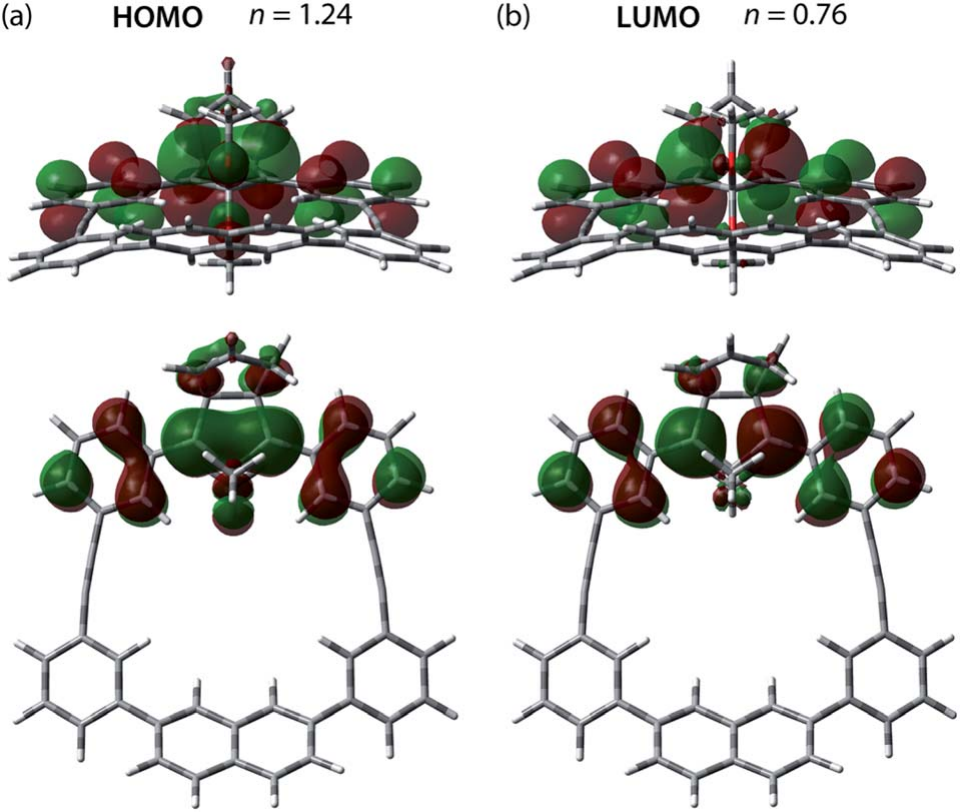
(a) HOMO (ψ_S_) and (b) LUMO (ψ_A_) orbitals and their occupation numbers (*n*) calculated for S-DR3b at the CASSCF(2,2)/6-31G(d) level of theory.

The effect of the macrocycle on the structure and reactivity of S-DR3b was evaluated by the complete active space self-consistent field (CASSCF)^69^ and broken-symmetry (BS)^70^ density functional theory (BS-DFT) methods at the (U)ωB97X-D/6-31G(d)^71–73^ level of theory within the Gaussian 16 ^74^ package. The effect was compared with that in S-DR3a. First, the singlet ground state of DR3b was confirmed by computing the singlet-triplet energy gap (Δ*E*_S–T_ = −7.49 kJ mol^−1^), which is similar to those of DR2b and DR3a (Δ*E*_S–T_ = −7.68 and −7.58 kJ mol^−1^), entries 1–3 in Table 1. Thus, the macrocyclic structure does not affect the relative energy difference between the two spin states. Next, the π-single bonding character (*i.e.*, the diradical character) of S-DR3b was compared to that of S-DR2b by calculating the occupation number of the HOMO and LUMO orbitals at the CASSCF(2,2)/6-31G(d) level of theory. The calculated HOMO and LUMO occupation numbers of S-DR3b were 1.24 and 0.76, respectively (Fig. 1), whereas those of S-DR2b were 1.37 and 0.63, respectively.^49^ Thus, the computations indicate that the energetic stabilisation of S-DR3b by electron delocalisation over the *meta*-connected π-conjugated system is negligible.

The kinetic stabilisation of S-DR3b by the macrocyclic structure was evaluated by comparing its computational results with those of S-DR2b at the same level of theory (Table 1, entries 1–3). Calculations of the ring-closed products CP were performed using the restricted method at the (R)ωB97X-D/6-31G(d) level of theory, whereas the corresponding transition states TS were assessed by computing the vibrational frequency and their intrinsic reaction coordinate (IRC, Fig. S17 in ESI†). According to the IRC calculations, the transition states of the ring-closing reactions towards *cis*- and *trans*-TS3b produced the metastable ring-closed conformers *par*-CP3b with a face-to-face orientation of the benzene rings (*par*-CP structure, Table 1). The barrierless *par-twi* isomerisation afforded the more stable conformers *cis-twi*- and *trans-twi*-CP3b with a nearly perpendicular orientation of the phenyl residues (*twi*-CP structure, Table 1). As expected, the energies of the ring-closed products *cis*- and *trans-twi*-CP3b were 35.15 and 37.00 kJ mol^−1^ higher (Δ*E*_DR__–__CP_) than those of *cis*- and *trans-twi*-CP2b, respectively (entries 1 and 3). The corresponding energy differences with CP3a were found to be 19.99 and 21.29 kJ mol^−1^ (entries 2 and 3). Thus, the difference between the energies of the singlet diradical and ring-closed product significantly decreased upon introduction of the macrocyclic structure in 3b. Moreover, the small energy difference between closed-shell *cis-twi*-CP3b and S-DR3b (Δ*E*_DR__–__CP_ = 3.99 kJ mol^−1^) suggests a significant contribution of the stretch effect to the increase in the molecular strain of CP3b, thus kinetically stabilising S-DR3b. Additionally, the stretch effect is reflected in the longer C–C bonds calculated for CP3b (Table 1, entries 1–3, values in brackets). Furthermore, this effect is significant in the parallel conformation of *par*-CP. The transition state enthalpies of *cis*- and *trans*-TS3b were larger than those of TS2b and TS3a by 5.91 and 8.60, 3.20 and 7.71 kJ mol^−1^, respectively. According to the computational analyses, the stretch effect in the newly designed macrocyclic structure is expected to provide a long-lived singlet diradical.

**Table 1:** Computational data and energy profiles of the ring-closing reaction of the parent species S-DR2b and its macrocyclic derivative S-DR3a,b

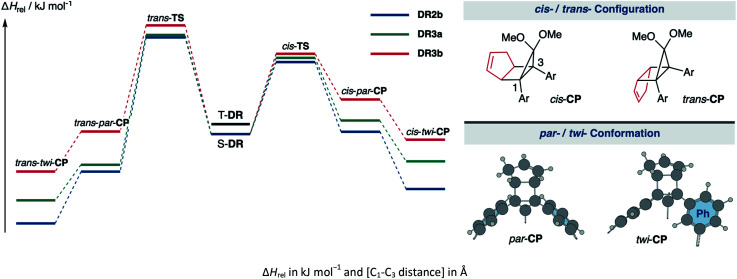										
Entry	Functions		S-DR	T-DR	*cis*-TS	*cis-par*-CP	*cis-twi*-CP	*trans*-TS	*trans-par*-CP	*trans-twi*-CP
1	ωB97X-D	2b	0.00 [2.380]	7.68 [2.399]	51.37 [2.063]	1.66 [1.594]	−39.14 [1.585]	68.92 [2.054]	−26.74 [1.576]	−63.66 [1.566]
2		3a	0.00 [2.379]	7.58 [2.399]	54.08 [2.047]	7.67 [1.600]	−23.98 [1.584]	69.81 [2.058]	−24.53 [1.574]	−47.95 [1.565]
3		3b	0.00 [2.381]	7.49 [2.400]	57.28 [2.041]	24.67 [1.622]	−3.99 [1.590]	77.52 [2.112]	1.86 [1.587]	−26.66 [1.567]
4	B3LYP	2b	0.00 [2.389]	9.43 [2.413]	44.23 [2.070]	24.99 [1.612]	−16.66 [1.608]	65.41 [2.016]	1.48 [1.589]	−37.88 [1.582]
5		3a	0.00 [2.389]	9.10 [2.413]	50.35 [1.989]	29.48 [1.630]	−0.30 [1.610]	66.58 [2.053]	1.82 [1.592]	−20.06 [1.583]
6		3b	0.00 [2.391]	9.08 [2.414]	55.29 [1.940]	53.25 [1.681]	17.56 [1.623]	76.21 [2.116]	36.81 [1.614]	8.83 [1.585]
7	CAM-B3LYP	2b	0.00 [2.382]	7.22 [2.401]	56.88 [2.030]	17.51 [1.592]	−25.96 [1.586]	76.37 [2.022]	−7.62 [1.572]	−48.20 [1.566]
8		3a	0.00 [2.383]	7.00 [2.401]	61.82 [2.011]	20.99 [1.601]	−9.47 [1.587]	77.38 [2.027]	−8.72 [1.574]	−30.93 [1.566]
9		3b	0.00 [2.384]	6.98 [2.402]	66.45 [2.004]	46.07 [1.631]	9.49 [1.596]	84.55 [2.082]	26.08 [1.591]	−10.52 [1.570]
10	M06-2x	2b	0.00 [2.372]	7.93 [2.393]	40.22 [2.132]	−14.71 [1.590]	−59.63 [1.582]	56.30 [2.131]	−42.02 [1.576]	−81.38 [1.563]
11		3a	0.00 [2.372]	7.70 [2.392]	42.08 [2.122]	−8.75 [1.597]	−43.69 [1.581]	58.27 [2.135]	−40.17 [1.572]	−65.51 [1.562]
12		3b	0.00 [2.373]	7.72 [2.394]	45.00 [2.118]	6.95 [1.620]	−23.53 [1.588]	68.41 [2.126]	−14.36 [1.583]	−30.43 [1.564]
13	ωB97	2b	0.00 [2.386]	6.25 [2.402]	62.27 [2.029]	−6.21 [1.576]	−49.45 [1.569]	80.40 [2.023]	−33.52 [1.560]	−73.87 [1.555]
14		3a	0.00 [2.386]	6.20 [2.402]	67.11 [2.013]	−1.36 [1.581]	−33.51 [1.570]	81.94 [2.024]	−33.54 [1.562]	−56.65 [1.554]
15		3b	0.00 [2.387]	6.13 [2.403]	71.62 [2.007]	21.30 [1.598]	−13.57 [1.575]	87.36 [2.075]	−2.49 [1.572]	−36.07 [1.557]
16	APF-D	2b	0.00 [2.371]	8.95 [2.393]	40.57 [2.070]	5.00 [1.593]	−37.86 [1.588]	60.70 [2.060]	−20.17 [1.576]	−59.76 [1.568]
17		3a	0.00 [2.370]	8.67 [2.392]	42.66 [2.054]	11.92 [1.601]	−23.08 [1.586]	60.33 [2.067]	−19.09 [1.575]	−45.06 [1.566]
18		3b	0.00 [2.371]	8.70 [2.393]	45.19 [2.047]	26.61 [1.627]	−3.09 [1.591]	69.16 [2.095]	7.02 [1.587]	−24.57 [1.567]

Computations were also conducted at the B3LYP,^75^ CAM-B3LYP,^76^ M06-2x,^77^ ωB97,^78^ and APF-D^79^ functions with the 6-31G(d) basis set. Although the relative energies computed by distinct methods were different, the general tendencies corroborate the stretch effect. For example, *cis*- and *trans-twi*-CP3b were higher in energy than S-DR3b by the B3LYP method (entry 6), whereas the data obtained by other computational methods indicates that the twisted ring-closed compounds are more stable than S-DR3b. The energy differences between S-DR3b and CP3b were much smaller than between S-DR2b,3a and CP2b,3a owing to the macrocyclic structure. Notably, the difference in the enthalpy between S-DR2b and *cis*-TS2b computed using the M06-2x and APF-D methods (Δ*H*_rel_ = 40.22 and 40.57 kJ mol^−1^, entries 10 and 16 respectively) were closest to the experimental activation energy values for the reaction of S-DR2b to *cis*-CP2b (*E*_a_ = 30.5 ± 0.4 kJ mol^−1^).^49^

**Fig. 2:**
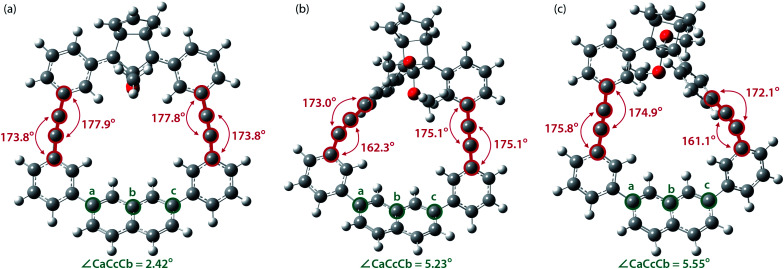
Bending angles of triple bonds and naphthyl moieties in (a) S-DR3b, (b) *cis-twi*-CP3b, and (c) *trans-twi*-CP3b, optimised at the (R,U)ωB97X-D/6-31G(d) level of theory.

To gain a deeper understanding of the effect of the designed macrocyclic skeleton, the geometry of the triple bonds and naphthyl moiety were analysed (Fig. 2).^100^ The triple bonds in S-DR3b were slightly bent to 178° and 174°, whereas the naphthyl moiety deviated from planarity by 2.4° (Fig. 2a). Larger values were obtained for *cis-twi*-CP3b, in which the bending angles of the triple bonds and naphthyl moiety were 162° and 5.2°, respectively (Fig. 2b). A similar bent structure was also confirmed for *trans-twi*-CP3b (Fig. 2c). Subsequently, the effect of bending on the molecular strain was assessed by computing the strain energies (SE_M_) of the macrocyclic units in AZ3b, S-DR3b, *cis*-CP3b, and *trans*-CP3b at the (R,U)ωB97X-D/6-31G(d) level of theory (Table 2a), since the structural parameters computed by this method were well aligned with the experimental data (X-ray crystallography, Table S2 in ESI†). The corresponding values were compared with those in 3a. The value of SE_M_ was calculated by subtracting the total electronic energy of the non-strained macrocyclic structure (nS), 2,7-bis(3-(phenylethynyl)-phenyl)naphthalene, from the energy of strained macrocycles in AZ-S, DR-S, *cis*-CP-S, and *trans*-CP-S. The latter were obtained by replacing the azo, diradical, and ring-closing units in AZ3a,b, S-DR3a,b, *cis*-CP3a,b, and *trans*-CP3a,b with two hydrogen atoms (Table 2a, example for 3b). Their energies were obtained by partial optimisation of the C–H bonds without optimising other moieties. The strain energies in AZ3a,b and S-DR3a,b were relatively small (entries 1–4, SE_M_ = 8.01 and 11.49 for 3a; 11.24 and 16.38 kJ mol^−1^ for 3b, respectively), whereas larger values were obtained for CP3a,b with bent alkynes (entries 5–12, approximately 15–19 for 3a; 29–38 for 3b kJ mol^−1^). Thus, the strain energy of 3b was found to be larger than that of 3a.

**Table 2:** Strain energy of the macrocycle (SE_M_) and molecular strain energy (SE) calculated at the (R,U)ωB97X-D/6-31G(d) level of theory

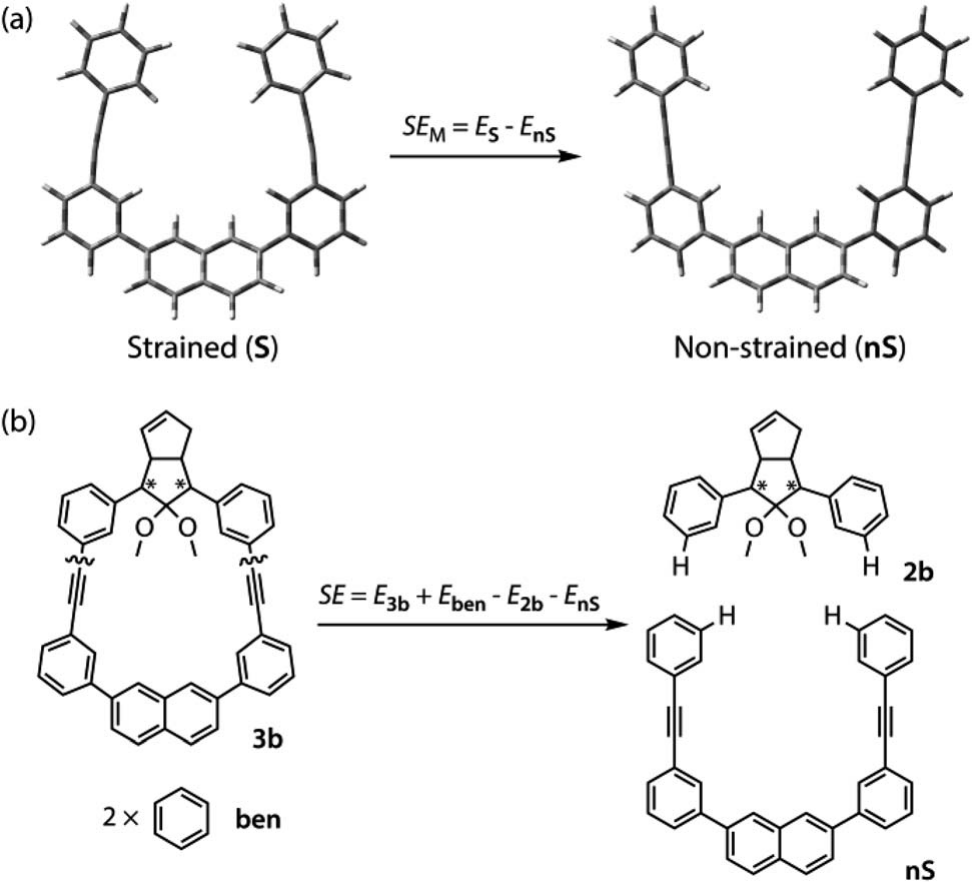				
Entry	Compounds		Energies in kJ mol^−1^	
			SE_M_	SE
1	AZ	3a	8.01	9.25
2		3b	11.24	17.45
3	S-DR	3a	11.49	5.11 (0.00)^a^
4		3b	16.38	8.92 (0.00)^b^
5	*cis-par*-CP	3a	19.19	11.47 (6.36)^a^
6		3b	29.13	32.18 (23.26)^b^
7	*cis-twi*-CP	3a	14.62	19.63 (14.52)^a^
8		3b	36.26	43.66 (34.74)^b^
9	*trans-par*-CP	3a	21.45	6.28 (1.17)^a^
10		3b	34.85	36.18 (27.26)^b^
11	*trans-twi*-CP	3a	15.31	19.97 (14.87)^a^
12		3b	38.56	45.08 (36.16)^b^

a Values relative to S-DR3a.

b Values relative to S-DR3b.

The molecular strain energies (SE) of AZ3a,b, S-DR3a,b, *cis*-CP3a,b, and *trans*-CP3a,b, which were estimated using the isodesmic reaction (Table 2b, example for 3b) and compared to the standard AZ2b, S-DR2b, *cis*-CP2b, and *trans*-CP2b, were larger than the corresponding SE_M_, with the exception of S-DR3a,b (entries 3 and 4). The strain energies relative to S-DR3a,b, which are indicated in parenthesis in Table 2 (entries 3–12), were very similar to the differences between the corresponding Δ*E*_DR__–__CP_ of 2b and 3a,b (ΔΔ*E*_DR__–__CP_ = 6.01, 15.16, 2.21, and 15.71 kJ mol^−1^ for 3a; 23.01, 35.15, 28.60, and 37.00 kJ mol^−1^ for 3b, respectively, Table 1, entries 1–3), indicating that the molecular strain strongly correlates with the macrocyclic structures. The molecular strain of 3b was larger than that of 3a. Furthermore, the computations clearly indicate that the kinetic stabilisation of S-DR3b by the macrocyclic scaffold suppresses bond formation in the singlet diradicaloid.

### Synthesis and characterisation

**Scheme 3:**
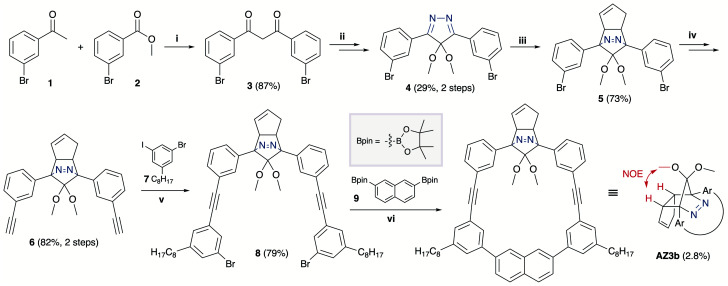
Synthetic route towards AZ3b. (i) NaH, THF, 0 °C, 20 h; (ii) 1. Ph_2_Se_2_, (NH_4_)_2_S_2_O_8_, MeOH, 75 °C, 3.5 h; 2. N_2_H_4_·H_2_O, CHCl_3_, 70 °C, 16 h; (iii) cyclopentadiene, TFA, DCM, 0 °C, 1.5 h; (iv) trimethylsilylacetylene, Pd(PPh_3_)_4_, CuI, TEA, THF, 60 °C, 43.5 h; 2. K_2_CO_3_,THF, MeOH, r.t., 16.5 h; (v) Pd(PPh_3_)_4_, CuI, TEA, THF, 60 °C, 39.5 h; (vi) Pd(OAc)_2_, SPhos, K_3_PO_4_, THF, H_2_O, 80 °C, 21 h.

The synthesis of azoalkane AZ3b, a precursor to S-DR3b, is shown in Scheme 3. Claisen condensation of 1 and 2 led to 3, which was dimethoxylated by oxidation with Ph_2_Se_2_ in MeOH. Subsequently, cyclisation with N_2_H_4_ afforded pyrazole 4. The Diels–Alder cycloaddition of 4 and cyclopentadiene delivered azoalkane 5, which was subjected to two sequential Sonogashira cross-coupling reactions to produce azoalkane 8.^68^ Macrocyclisation was accomplished by the one-pot consecutive inter- and intramolecular Suzuki–Miyaura coupling of azoalkane 8 and bisboronic ester 9 in the presence of Pd(OAc)_2_ and SPhos ligand,^80^ which delivered azo compound AZ3b in approximately 3% isolated yield with regard to 8. The flanking alkyl substituents (–C_8_H_17_) are required to ensure sufficient solubility.

**Fig. 3:**
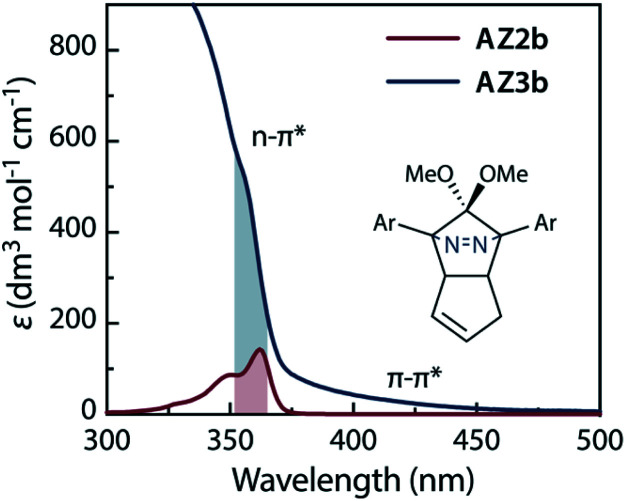
UV-vis absorption spectra of AZ2b (2.87 mM) and AZ3b (2.13 mM) in benzene at 298 K.

The molecular structure of AZ3b was confirmed by nuclear magnetic resonance spectroscopy (^1^H, ^13^C NMR) and ESI mass spectrometry (MS). The *endo* configuration was confirmed by the observed correlation between a methoxy group and the bridgehead protons in the two-dimensional nuclear Overhauser effect spectrum (NOESY, Scheme 3). Furthermore, X-ray crystallographic analysis of AZ3b corroborated the molecular structure, although disorder was observed for the octyl chains (Fig. S7 in ESI†). The UV-vis absorbance spectrum of AZ3b exhibits a maximum absorption at 356 nm (*ε*_356_ = 517 dm^3^ mol^−1^ cm^−1^), which is analogous to that of AZ2b (*ε*_358_ = 112 dm^3^ mol^−1^ cm^−1^). Hence, it stems from the overlap of the n–π* electronic transition of the azo chromophore with the π–π* one of the π-conjugated system, whereas the broad absorption band up to 450 nm corresponds to the π-conjugation in the macrocycle (Fig. 3).

### Analyses of the azoalkane photolysis and its products

**Fig. 4:**
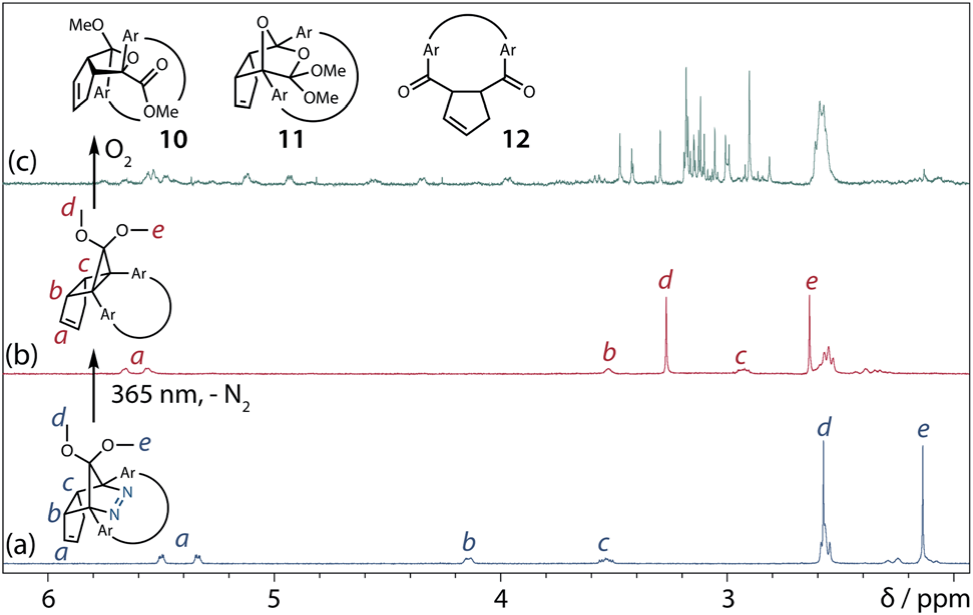
*In situ* ^1^H NMR (400 MHz) analysis of the photoreaction of AZ3b (4.47 mM in degassed C_6_D_6_). ^1^H NMR spectrum of (a) AZ3b before irradiation; (b) *trans*-CP3b after irradiation with a 365 nm LED lamp for 90 s at 298 K under nitrogen atmosphere; and (c) *trans*-CP3b decomposition upon exposure to air for 3 min.

**Scheme 4:**
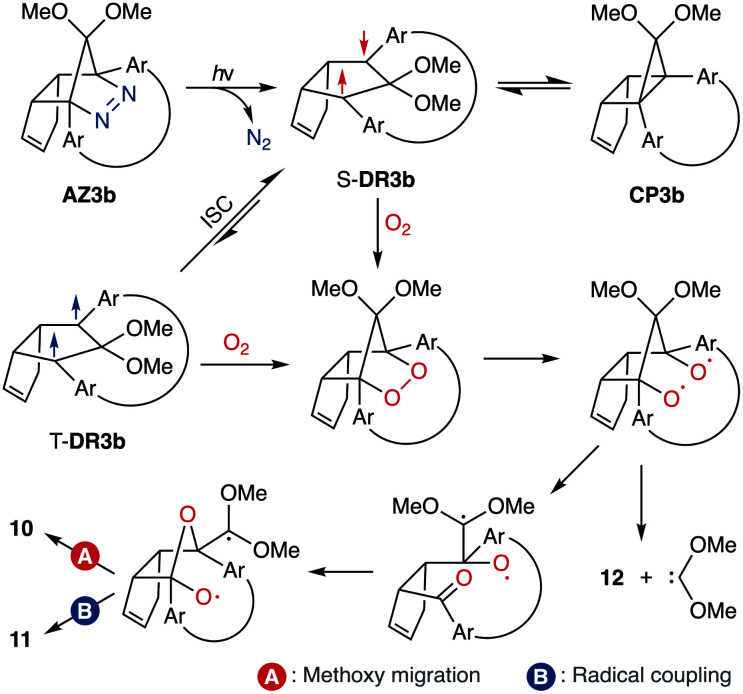
Proposed oxidation mechanism.

To gain insight into the ring-closing processes of S-DR3b, the photoreaction was carried out by subjecting AZ3b (4.47 mM in C_6_D_6_) to irradiation (*λ*_exc_ = 365 nm, LED lamp) in a sealed NMR tube under N_2_ atmosphere at 298 K. Direct analysis of the reaction mixture by ^1^H NMR spectroscopy (Fig. 4), including nuclear Overhauser effect measurements (NOE, Fig. S4 in ESI†), revealed the quantitative formation of the ring-closed product *trans*-CP3b (Fig. 4b). This sensitive compound (Fig. 4c) reacted with air to oxygenated products 10–12, which were isolated by preparative thin-layer chromatography and fully characterised by NMR and MS (Fig. S10 in ESI†). The fast decomposition of macrocycle-embedded *trans*-CP3b is likely due to the stretch effect, since the analogous photoproduct *trans*-CP2b without the macrocycle was stable towards air at 60 °C (Fig. S8 in ESI†). The proposed mechanism for the oxidation of *trans*-CP3b proceeds *via* the endoperoxide (Scheme 4), as suggested for the thermal decomposition of CP2b under air.^51^

**Fig. 5:**
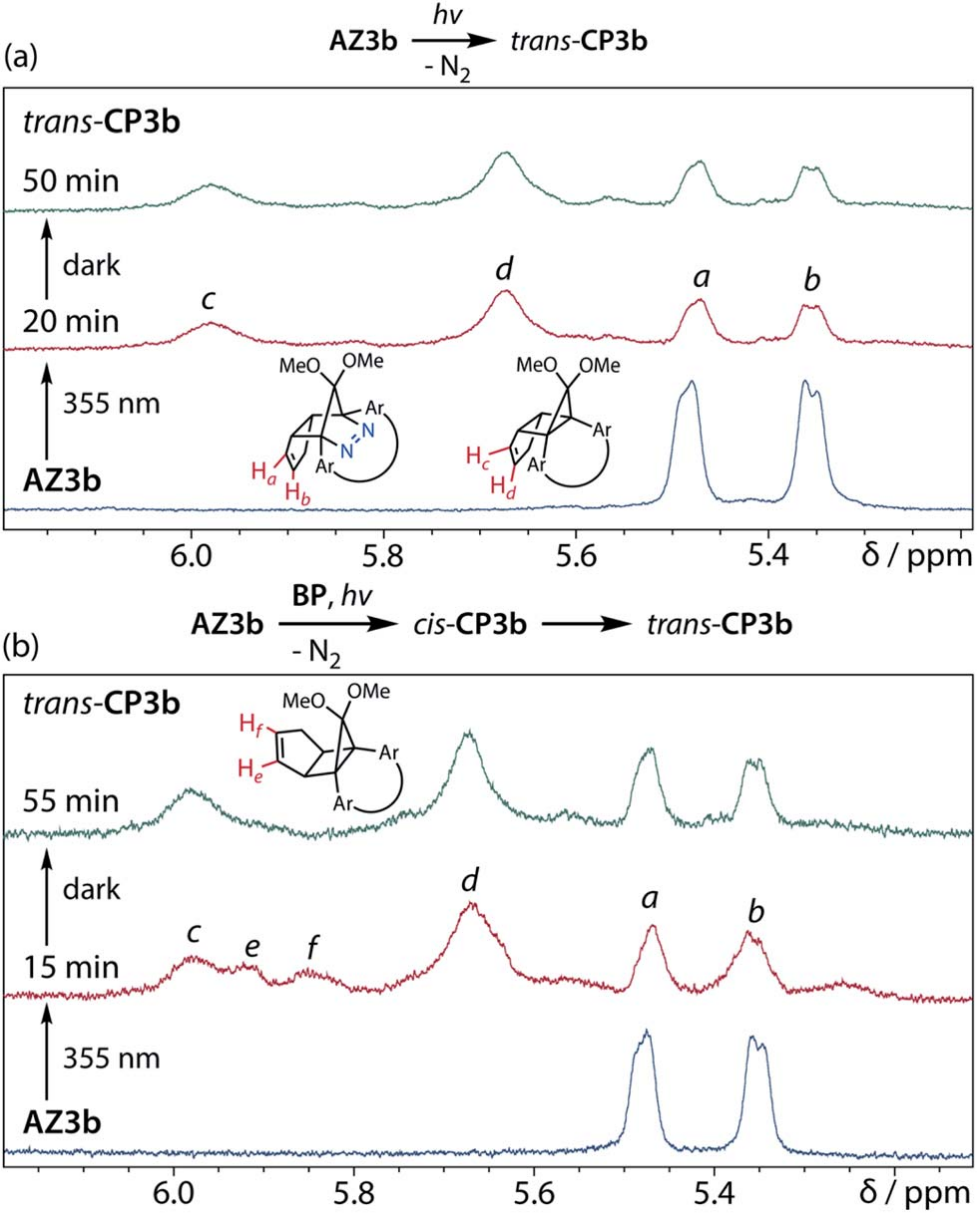
*In situ* ^1^H NMR (400 MHz) analysis of the N_2_ loss of AZ3b in degassed toluene-*d*_8_ at 199 K with Nd:YAG laser irradiation (*λ*_emi_ = 355 nm, 30 mJ). (a) Direct irradiation of AZ3b (6.49 mM); (b) Irradiation of AZ3b (0.60 mM) in the presence of benzophenone (9.32 mM).

**Scheme 5:**
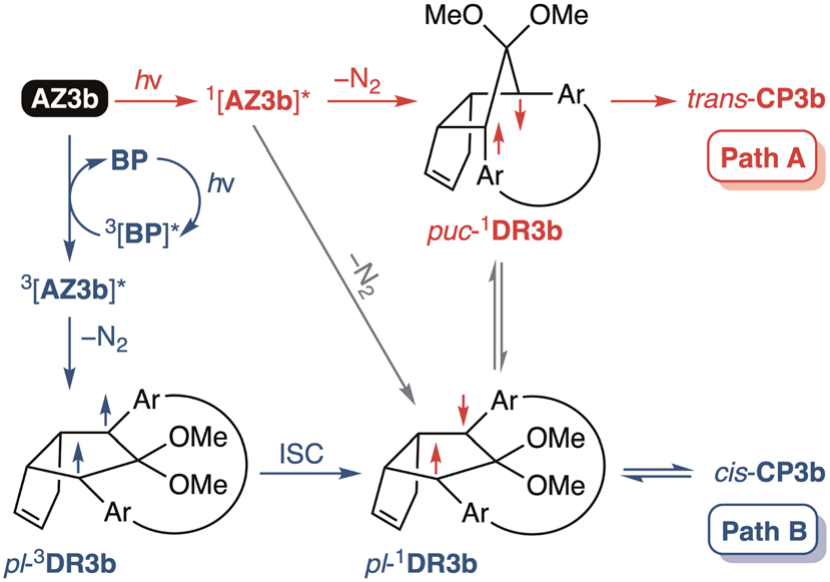
Decay pathways of photoexcited AZ3b.

Furthermore, *trans*-CP3b, which was computed to be energetically more stable than *cis*-CP3b (Table 1), was formed by photolysis of AZ3b at ∼25 °C (Fig. 4), although the calculated energy barrier for the formation of *cis*-CP3b was lower than that of *trans*-CP3b (Table 1). As the isomerisation of *cis*-CP3b to *trans*-CP3b is supposedly inhibited by a large activation energy (>70 kJ mol^−1^ from S-DR3b to *trans*-CP3b at 199 K, Table 1), low-temperature ^1^H NMR experiments were conducted to identify the primary product of the reaction at 199 K. To this end, the photolysis of AZ3b was carried out in degassed toluene-*d*_8_ (6.49 mM) under irradiation with a Nd:YAG laser (30 mJ per pulse, 355 nm), which was introduced into the NMR tube by a quartz rod.^81^*In situ*^1^H NMR monitoring of the reaction revealed the sole formation of *trans*-CP3b (vinylic signals c and d, Fig. 5a) alongside unreacted AZ3b (signals a and b). The exclusive formation of *trans*-CP3b is explained by the existence of the puckered diradical *puc*-^1^DR3b (path A, Scheme 5).^82^ Using the same experimental setup, a degassed toluene-*d*_8_ solution of AZ3b (0.60 mM, Abs_355_ = 0.32) and triplet sensitiser benzophenone (9.32 mM, Abs_355_ = 1.09) was irradiated (355 nm, 199 K). Interestingly, the NMR spectra acquired *in situ* contained new signals (e and f, approximately 5.9 ppm, Fig. 5b), which were converted to signals c and d in the dark. Hence, signals e and f correspond to *cis*-CP3b, which subsequently isomerises to the more stable *trans*-CP3b. The mechanism of the benzophenone-sensitised *cis*-CP3b formation involves the planar diradical intermediate *pl*-^1^DR3b, which is associated with a smaller activation energy (path B, Scheme 5).

### UV-vis absorption and EPR studies of the low-temperature AZ3b photolysis

**Fig. 6:**
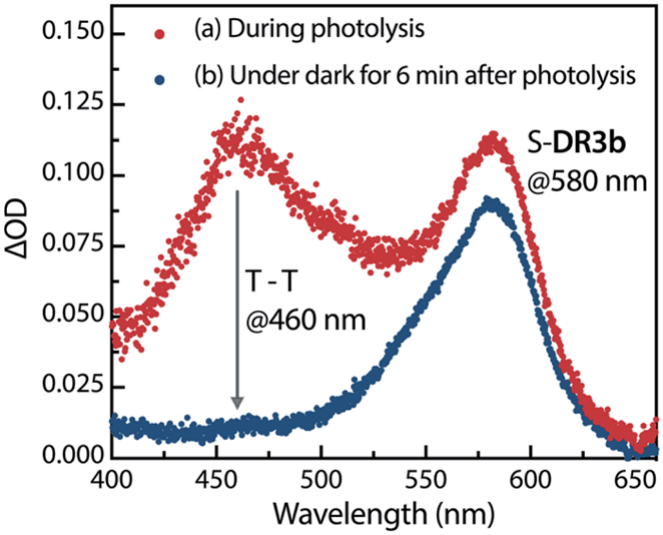
Low-temperature UV-vis absorption spectra of the photolysis of AZ3b (2.46 mM) in a degassed MTHF matrix at 90 K.

The experimental evidence presented above clearly suggests that S-DR3b is generated from AZ3b*via* photochemical nitrogen extrusion. To directly detect planar S-DR3b, low-temperature UV-vis absorbance measurements were performed during the photolysis of AZ3b (2.46 mM) in a degassed 2-methyltetrahydrofuran (MTHF) matrix under irradiation (*λ*_exc_ = 360 ± 10 nm, Xe lamp) at 90 K (Fig. 6). Two absorption bands were observed at 460 and 580 nm during the reaction (Fig. 6a). Once irradiation was ceased, the band at 460 nm disappeared within seconds, whereas that at 580 nm remained unchanged for over 3 h at 90 K under dark conditions (Fig. 6b). These observations suggest that the absorption at 460 nm stems from an electronically excited state, whereas the long-lived absorption band at 580 nm was assigned to the HOMO–LUMO electronic excitation (π–π*) of singlet diradicaloid S-DR3b, as it is typical for singlet diradicaloids S-DR2 (Scheme 1).^83^

**Fig. 7:**
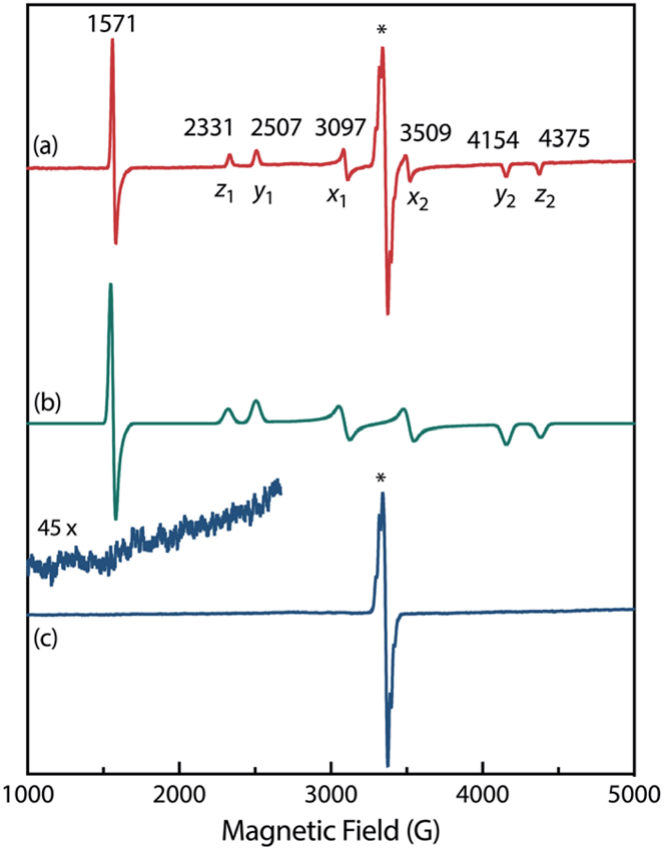
Experimental EPR spectra of AZ3b (4.92 mM in a MTHF matrix at 80 K) (a) during photolysis and (c) in the dark after irradiation; inset: enhanced view from 1000–2670 G. (b) EPR spectrum simulated using *D*/*hc* = 0.096 cm^−1^ and *E*/*hc* = 0.019 cm^−1^. The asterisk indicate signals that correspond to impurities in MTHF.

To confirm the spin multiplicities of the species associated with the absorption bands at 460 and 580 nm, low-temperature electron paramagnetic resonance (EPR) spectroscopy was conducted during the photochemical reaction of AZ3b (4.92 mM) in an MTHF matrix irradiated with a Hg lamp (*λ*_exc_ > 250 nm) at 80 K (Fig. 7). EPR signals typical of triplet species were observed during photolysis at 2331 (*z*_1_), 2507 (*y*_1_), 3097 (*x*_1_), 3509 (*x*_2_), 4154 (*y*_2_), and 4375 Gauss (G) (*z*_2_) corresponding to the allowed transition (|Δ*m*_s_| = 1) at 9.4 GHz resonance frequency (Fig. 7a). In addition, the half-field signal (|Δ*m*_s_| = 2) was detected at 1571 G. The signals at approximately 3400 G correspond to doublet impurities, which were also observed in a control experiment (MTHF irradiation under the same conditions). The obtained zero-field splitting (zfs) parameters of the triplet species were *D*/*hc* = 0.096 cm^−1^ and *E*/*hc* = 0.019 cm^−1^. The EPR spectrum simulated with *D*/*hc* = 0.096 cm^−1^ and *E*/*hc* = 0.019 cm^−1^ (Fig. 7b) reproduced well the experimental spectrum. The obtained zfs parameters were similar to those of the triplet excited state of 2-phenylnaphthalene (*D*/*hc* = 0.0963 cm^−1^ and *E*/*hc* = 0.0274 cm^−1^).^84^ The *D* value of triplet 1,3-diphenyl-cyclopentane-1,3-diyl diradicals was reported to be approximately 0.05 cm^−1^,^16,85^ which is much smaller than that observed in this study. When the irradiation was ceased at 80 K, the triplet signals disappeared (Fig. 7c), confirming that singlet diradicaloid S-DR3b is associated with the UV-vis absorbance at 580 nm (Fig. 6). Further, the triplet signals were short-lived at 5 K and the lifetimes of the decay signal monitored at 1562 G were nearly the same in the temperature range of 5–80 K (*τ*_5_ = 2.20 s and *τ*_80_ = 2.25 s, Fig. S11 in ESI†). The temperature-independency of the decay process and the large zfs parameters support the hypothesis that these EPR signals correspond to the triplet excited state of the naphthyl unit in AZ3b. Thus, the UV-vis absorption band at 460 nm was assigned to the T–T absorption of the naphthyl moiety (Fig. 6). Indeed, the T–T absorption band of a model compound 3b′ was predicted to appear at 466 nm using the TD-DFT method at the ωB97X-D/6-31G(d) level of theory (Fig. S18 in ESI†).

To further confirm the assignment of the EPR triplet signals, the zfs parameters, *D*/*hc* and *E*/*hc*, were computed for the triplet states of DR2b, DR3a, and DR3b at the B3LYP/EPR-II^86^ level using the ORCA 4.2.1 program package^87,88^ (Table 3). To evaluate the accuracy of computed values, the experimentally known zfs values of triplet molecules DR4–6 ^85^ were also simulated at the same level of theory (entries 4–6). As shown in entries 4–6, the calculated (cald) zfs parameters, especially *D*/*hc* values, well reproduced the experimental values of triplet diradicals DR4–6. Thus, *D*/*hc* value of triplet state DR3b should be around 0.057 (entry 3), which is not consistent with the relatively large *D*/*hc* value of 0.096 cm^−1^ in the photolysis of AZ3b (Fig. 7a).

**Table 3:** Experimental and computed zfs parameters, *D*/*hc* and *E*/*hc*, of triplet state of DR2b, DR3a, DR3b, DR4–6

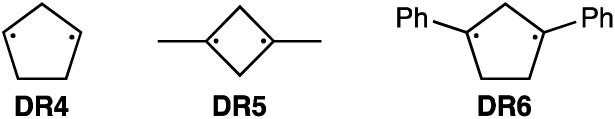			
Entry		zfs parameters (in cm^−1^) exp (calcd)^a^	
		*D*/*hc*	*E*/*hc*
1	DR2b	nd (0.061)	nd (0.0051)
2	DR3a	nd (0.059)	nd (0.0064)
3	DR3b	nd (0.057)	nd (0.0060)
4	DR4	0.084 (0.089)	0.0020 (0.0019)
5	DR5	0.112 (0.113)	0.0050 (0.0023)
6	DR6	0.045 (0.044)	0.0010 (0.0020)

a Calculated at the B3LYP/EPR-II level of theory using ORCA 4.2.1 program package.

### Time-resolved absorption spectroscopy

**Fig. 8:**
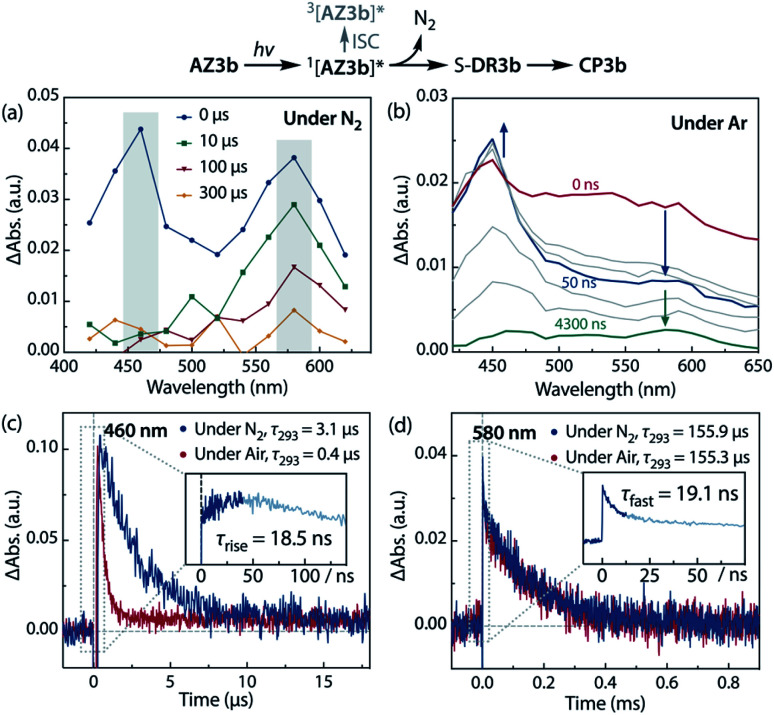
Transient absorption spectra during the laser flash photolysis of AZ3b (*λ*_emi_ = 355 nm) at 293 K in benzene. (a) Sub-microsecond transient absorption spectra of AZ3b (2.68 mM, Abs_355_ = 0.72) under N_2_; (b) sub-nanosecond transient absorption spectra of AZ3b (7.19 mM, Abs_355_ = 0.77) under Ar; time profile monitored at (c) 460 nm under N_2_ and air; inset: time delay from −15 to 140 ns under Ar; and (d) at 580 nm under N_2_; inset: time delay from −10 to 75 ns under Ar.

Sub-microsecond transient absorption (TA) spectroscopy was conducted to monitor the laser flash photolysis (LFP) of AZ3b (2.68 mM, Abs_355_ = 0.72) in degassed benzene at 293 K using a Nd:YAG laser (355 nm, 5 ns pulse, 7 mJ; Fig. 8a). In addition, sub-nanosecond TA spectroscopy (*λ*_exc_ = 355 nm, 25 ps pulse, 80 μJ) was employed to study the photolysis of AZ3b (7.19 mM, Abs_355_ = 0.77) in benzene using a randomly interleaved pulse train (RIPT)^89^ method under an Ar atmosphere (Fig. 8b). The strong TA bands observed at 460 and 580 nm (Fig. 8a and b) were similar to those observed in the low-temperature absorption measurements (Fig. 6). With a *τ*_293_ of 3.08 ± 0.02 μs, the transient species at 460 nm was assigned to the triplet excited state of the naphthyl moiety, as suggested by the EPR measurements. The lifetime of this species significantly decreased to 426 ± 4 ns upon exposure to air due to quenching by molecular oxygen with a rate constant *k*_q_(O_2_) of 1.10 × 10^9^ M^−1^ s^−1^ (Fig. 8c). In contrast, the transient species associated with the signal at 580 nm was not quenched by O_2_ (Fig. 8d), supporting its identification as singlet diradical S-DR3b. A dual decay process was observed at 580 nm (Fig. 8d, inset). The fast decay process was attributed to the depletion of the singlet excited naphthyl moiety because the fall rate constant (*k*_f_ = 5.24 × 10^7^ s^−1^) was consistent with the rise rate constant (*k*_r_ = 5.41 × 10^7^ s^−1^) of the triplet species at 460 nm (Fig. 8c, inset). Further, the single exponential decay process with *k*_d_ = 6.4 × 10^3^ s^−1^ (*τ*_293_ = 155.9 ± 3.3 μs) was observed for the slow decay of the signal at 580 nm, which corresponds to the ring-closing (σ-bond formation) reaction to *cis*-CP3b (Fig. 5). As predicted by the computations, the singlet diradical S-DR3b was extremely long-lived with a lifetime approximately 1000-fold that of S-DR2b (*τ*_293_ = 209 ns),^49^ and 11-fold that of S-DR3a (*τ*_293_ = 14.2 μs).^68^ Thus, the experimental results demonstrate that the stretch effect induced by the macrocyclic structure increases the kinetic stabilisation of the singlet diradicaloid.

Variable temperature laser flash photolysis (VT-LFP) measurements were conducted at five temperatures in the range of 273–303 K. The activation parameters *E*_a_, log *A*, Δ*H*^‡^, Δ*S*^‡^, and Δ*G*^‡^_293_ of the ring-closing reaction of S-DR3b to CP3b in benzene were determined by the Arrhenius and Eyring plots (Table 4, Fig. S15 in ESI†). The activation energy and enthalpy of this process, determined as 58.4 ± 1.1 and 56.0 ± 1.1 kJ mol^−1^, respectively, are approximately 28 and 6 kJ mol^−1^ higher than the values of the corresponding reactions of S-DR2b and S-DR3a, respectively (entry 3). Unlike the ring-closing process of S-DR2b, the corresponding reactions of S-DR3a,b are associated with positive activation entropies (entries 2,3), although the decay process (*i.e.* the intramolecular σ-bond formation event), is the same, suggesting that the transition states of theses reactions should be very similar.^68^ Indeed, the activation entropy of the ring-closing reaction of S-DR3b computed at the (U)ωB97X-D/6-31G(d) level of theory was −22.04 J mol^−1^ K^−1^. This unusual observation prompted us to investigate the effect of the solvent on the lifetime in more detail.

**Table 4:** Lifetime *τ*_293_ of singlet diradicals S-DR at 293 K and activation parameters (*E*_a_, log *A*, Δ*H*^‡^, Δ*S*^‡^, Δ*G*^‡^_293_) of the ring-closing process in benzene

Entry	S-DR	*τ*_293_/μs	*E*_a_/kJ mol^−1^	log *A*/s^−1^	Δ*H*^‡^/kJ mol^−1^	Δ*S*^‡^/J mol^−1^ K^−1^	Δ*G*^‡^_293_/kJ mol^−1^
1	2b	0.21 ± 0.01	30.5 ± 0.4	12.1 ± 0.1	28.0 ± 0.4	−21.5 ± 0.8	34.2 ± 0.8
2	3a	14.2 ± 0.8	52.3 ± 0.4	14.1 ± 0.1	49.7 ± 0.4	17.1 ± 1.2	44.7 ± 0.4
3	3b	155.9 ± 3.3	58.4 ± 1.1	14.2 ± 0.2	56.0 ± 1.1	18.1 ± 2.3	50.7 ± 1.1

### Effect of the solvent on the reactivity of singlet diradicals

**Scheme 6:**
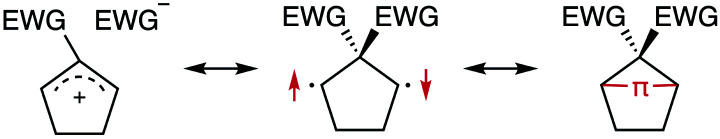
Resonance structures of singlet diradical species.

According to previous studies, the zwitterionic character of singlet diradicaloids such as S-DR2b presumably renders their lifetimes dependent on the polarity of the solvent (Scheme 6),^51^ with longer lifetimes expected in polar solvents. To investigate this relationship and the influence of the stretch effect on the diradical's stability in solution, LFP measurements of AZ2b and AZ3b were conducted in carbon tetrachloride, diethyl ether, ethyl acetate, toluene, 1,4-dioxane, acetone, glycerin triacetate (GTA), chloroform, dichloromethane, 1,2-dichloroethane, and dimethyl sulfoxide, thus encompassing a broad range of polarity (π*)^90,91^ and viscosity (*η*)^92^ (Table 5). In this study, the Kamlet–Abboud–Taft π* scale was used as an empirical polarity parameter because it was found to more suitably describe the effect of solvent polarity on the lifetime of S-DR2b than parameters such as *E*_T_(30)^93^ and dielectric constant^94^ (Table 5 and Fig. S16 in ESI†). The determination of the diradical's lifetime in acetonitrile and *n*-hexane was hampered by the insolubility of AZ3b in these solvents. Interestingly, among low viscous solvents including diethyl ether (*η* = 0.24 cP, entry 3), the longest lifetime of S-DR3b was measured in benzene (*η* = 0.65 cP, *τ*_293_ = 155.9 μs, entry 7). This value was over five-fold that in acetone (*η* = 0.32 cP, *τ*_293_ = 27.9 μs, entry 8), although acetone is more polar than benzene (π* = 0.62 and 0.55 kcal mol^−1^, respectively). Regarding highly viscous solvents, the lifetime of S-DR3b was surprisingly long in GTA (*η* = 23.0 cP, *τ*_293_ = 400.2 μs, entry 9). This value is 2.5- and 13-fold those in benzene and acetone, respectively, despite the similar polarity of these solvents (π*(GTA) = 0.63 kcal mol^−1^). In addition, the activation parameters of the ring-closing process were larger in GTA than in other solvents. In particular, the activation entropy significantly increased to 57.5 J mol^−1^ K^−1^ (Table S6 in ESI†). In contrast, the smallest activation entropy was determined in diethyl ether (*η* = 0.24 cP, Δ*S*^‡^ = 8.5 J mol^−1^ K^−1^), the lowest viscous solvent. These interesting observations indicate that both solvent polarity and viscosity play important roles in the ring-closing process of singlet diradicaloids.

**Table 5:** Lifetime *τ*_293_ of singlet diradicals S-DR2b and S-DR3b at 293 K in different solvents encompassing a wide range of polarity (π*) and viscosity (*η*). The numbers in parentheses indicate the ascending order of the values in each column

Entry	Solvent	π*/kcal mol^−1^	*η* (20 °C)/cP	*τ*_293_ of S-DR2b/ns	*τ*_293_ of S-DR3b/μs
1	*n*-Hexane	−0.11 (1)	0.31 (2)	90.1 (1)	nd
2	Tetrachloride carbon	0.21 (2)	0.97 (10)	187.1 (5)	17.2 (1)
3	Diethyl ether	0.24 (3)	0.24 (1)	136.3 (2)	46.8 (5)
4	Ethyl acetate	0.45 (4)	0.46 (5)	182.6 (4)	73.1 (8)
5	Toluene	0.49 (5)	0.59 (7)	170.4 (3)	116.5 (10)
6	1,4-Dioxane	0.49 (5)	1.18 (11)	250.6 (8)	61.4 (6)
7	Benzene	0.55 (7)	0.65 (8)	210.0 (6)	155.9 (11)
8	Acetone	0.62 (8)	0.32 (3)	231.4 (7)	27.9 (3)
9	Glycerin triacetate	0.63 (9)	23.00 (13)	517.1 (13)	400.2 (12)
10	Chloroform	0.69 (10)	0.58 (6)	404.8 (12)	65.4 (7)
11	Dichloromethane	0.73 (11)	0.44 (4)	294.0 (9)	46.6 (4)
12	1,2-Dichloroethane	0.73 (11)	0.79 (9)	307.6 (10)	22.8 (2)
13	Dimethyl sulfoxide	1.00 (13)	2.24 (12)	393.5 (11)	95.1 (9)

As the radical–radical coupling reaction strongly depends on the solvent viscosity, the dynamic solvent effect should play an important role in the isomerisation of S-DR to CP.^95–98,101^ This effect can be expressed by eqn (1), where R, I, and P are the reactant, intermediate, and product, respectively. In a low-viscosity solvent, the conversion of I to P is the rate-limiting step (*k*_f_ ≫ *k*_1_) according to the transition state theory (TST). Thus, the observed rate constant (*k*_obs_) is nearly equal to *k*_1_.1
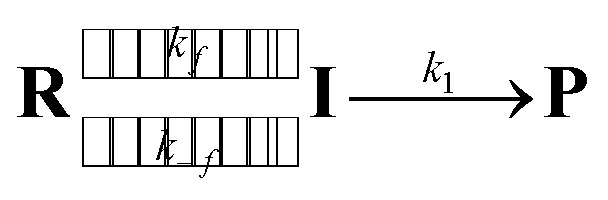


In high-viscosity solvents, the reaction rate *k*_obs_ is limited by solvent thermal fluctuations, rendering the TST no longer valid. Thus, the observed rate constant *k*_obs_ can be expressed by eqn (2):

2 1/*k*_obs_ = 1/*k*_TST_ + 1/*k*_f_,

where *k*_f_ and *k*_TST_ are the viscosity-dependent rate constant for solvent thermal fluctuation and viscosity-independent rate constant, respectively. If the dynamic solvent effect dominates the reaction (*k*_f_ ≪ *k*_TST_), the observed rate constant *k*_obs_ can be expressed by *k*_f_. In contrast, the observed rate constant can be expressed by *k*_TST_ if the solvent viscosity is low (*k*_obs_ ≈ *k*_TST_ ≈ *k*_1_).

**Fig. 9:**
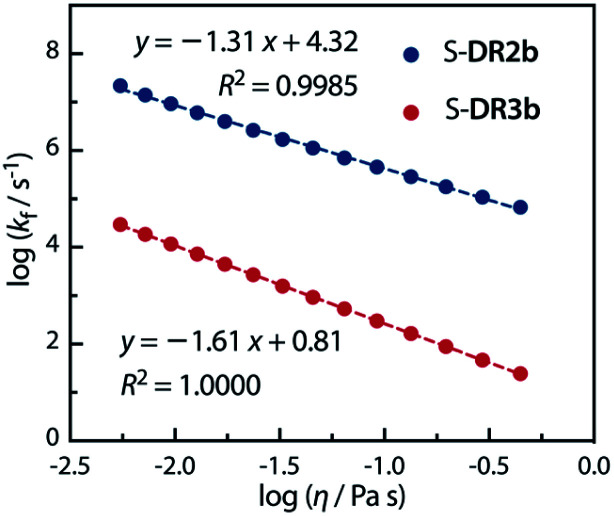
Dependence of the solvent thermal fluctuation rate constant log *k*_f_ calculated by eqn (2) on the viscosity of GTA. *k*_Ac_ and *k*_GTA_ were calculated from the Eyring plot.

Acetone (Ac, π* = 0.62 kcal mol^−1^, *η* = 0.32 cP) and GTA (π* = 0.63 kcal mol^−1^, *η* = 23.0 cP) are equally polar but very differently viscous. We assumed that the solvent thermal fluctuations in acetone are sufficiently fast to render the solvent dynamic effect due to the low viscosity negligible, such that *k*_TST_ ≈ *k*_Ac_ and *k*_obs_ ≈ *k*_GTA_. Thus, the rate constant for the solvent thermal fluctuation *k*_f_ can be estimated by *k*_f_ = (1/*k*_GTA_ − 1/*k*_Ac_)^−1^. The strong linear correlation between *k*_f_ and the viscosity of GTA^99^ proved the validity of the solvent dynamic effect for the singlet diradical system (Fig. 9).

**Fig. 10:**
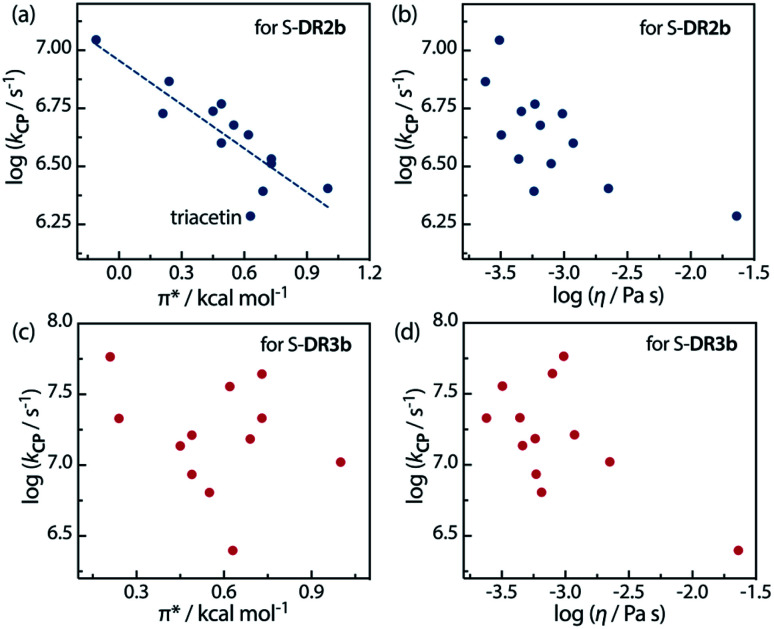
Correlations between the rate constant log *k*_CP_ and the solvent polarity (π*) and viscosity (*η*) for (a and b) S-DR2b and (c and d) S-DR3b.

Furthermore, the correlations between log *k*_CP_ and the polarity and viscosity are shown in Fig. 10, where *k*_CP_ (= *k*_d_ = 1/*τ*_293_ for S-DR3b) is the rate constant of the radical–radical coupling process. Regarding S-DR2b, a good correlation was observed between the solvent polarity and the lifetime, although the high viscosity of GTA led to a large deviation from the linear correlation (Fig. 10a). However, in the case of S-DR3b, obvious correlations between log *k*_CP_ and the polarity or viscosity of the solvents were not observed (Fig. 10c and d). Nevertheless, the effect of the viscosity on the lifetime suggests that the dynamic solvent effect should be considered to understand the phenomena.

The effect of solvent polarity (π*) and viscosity (*η*) on the lifetime of the singlet diradicals was further examined by performing a regression analysis according to eqn (3), in which *A* and *B* are the polarity and viscosity coefficients, respectively, and *C* is a constant term. All terms are compound-dependent.

3 *τ* = Aπ* + *Bη* + *C*

**Fig. 11:**
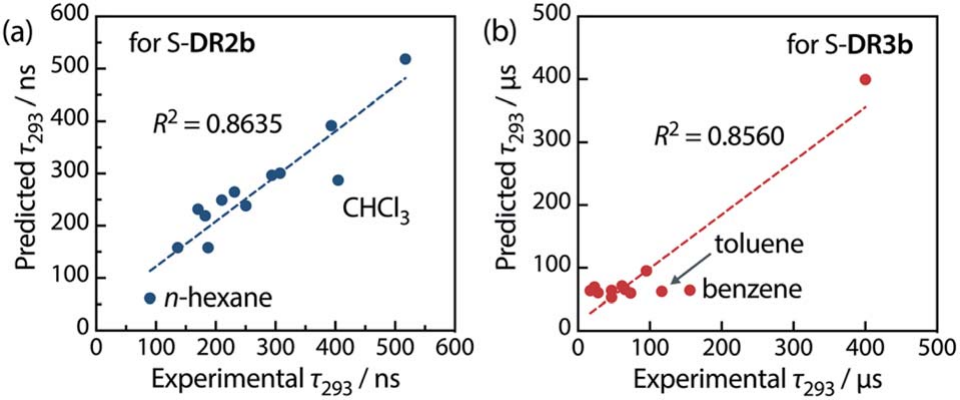
Correlation between the experimental and predicted lifetime values *τ*_293_ for (a) S-DR2b and (b) S-DR3b.

Table 6 lists the calculated coefficients for S-DR2b and S-DR3b. The polarity of the solvent is the dominant factor determining the lifetime of S-DR2b, as the corresponding coefficient is much larger than the viscosity one (278.43 and 11.06, respectively). In contrast, the coefficients *A* and *B* are similar for S-DR3b, suggesting that both polarity and viscosity strongly influence its lifetime (Table 6). The regression analyses were validated by plotting the experimental lifetime values *τ*_293_ was plotted against the ones predicted by eqn (3) (Fig. 11). A good linear correlation is observed in both cases (*R*^2^ = 0.86), despite the slight deviations of the data points corresponding to benzene and toluene (S-DR3b) and chloroform (S-DR2b). To gain insight into the effect of the macrocyclic structure on the relationship between viscosity and lifetime, the molecular volumes of S-DR2b and S-DR3b were computed at the (U)ωB97X-D/6-31G(d) level of theory. The obtained values of 497.04 and 303.44 cm^3^ mol^−1^ for S-DR3b and S-DR2b, respectively, indicate that the solvent viscosity effect is more pronounced in the ring-closing of S-DR3b to *cis*-CP3b than in the corresponding reaction of S-DR2b.

**Table 6:** Regression analyses for fitting the observed lifetime *τ*_293_ to eqn (3)

Coefficient	S-DR2b	S-DR3b
*A*	278.43	15 985.07
*B*	11.06	14 940.91
*C*	88.69	45 794.25

## Conclusions

Localised singlet diradicals are usually highly elusive species due to the fast radical–radical coupling reaction. In the past two decades, continuous efforts were made to extend the lifetime of putative intermediates and experimentally elucidate the bond homolysis process. In this study, singlet diradicaloid S-DR3b possessing a tailored macrocyclic structure was computationally designed and experimentally obtained from precursor AZ3b. S-DR3b was found to be extremely long-lived in benzene (*τ*_293_ = 155.9 μs), boasting approximately 1000-fold the lifetime of its analogue S-DR2b lacking the macrocycle. This substantial increase in the macrocyclic diradicaloid's lifetime is due to kinetic stabilisation resulting from the “stretch effect”, a recently introduced concept. Time-resolved laser flash photolysis studies of S-DR3b indicated that the reactivity of S-DR3b was largely influenced by the viscosity of the solvent, suggesting that the dynamic solvent effect plays an important role in the molecular transformation. S-DR3b was exceptionally long-lived in GTA (*τ*_293_ = 400.2 μs), with the thus far lowest rate constant of the carbon–carbon radical coupling process. Furthermore, the ring-closed product CP3b was thermally labile under air, producing oxidation products 10–12*via* an endoperoxide intermediate. Additionally, the formation of *cis*-CP3b and its isomerisation to *trans*-CP3b were observed at a low temperature (199 K). The experimental evidence provided herein corroborates the newly introduced stretch effect and quantifies its contribution to the stabilisation of singlet diradicals. Hence, this study establishes a new strategy towards a deeper understanding of the character and reactivity of singlet diradicaloids. The stretch effect confirmed in this study is expected to apply for kinetically stabilise other reactive intermediates.

## Conflicts of interest

There are no conflicts to declare.

## Supplementary Material

SC-012-D0SC05311B-s001
