# Supplementary material for: Impact of the macrocyclic structure and dynamic solvent effect on the reactivity of a localised singlet diradicaloid with π-single bonding character
Source: Chem Sci. 2020 Nov 10;12(2):613–25. doi: 10.1039/d0sc05311b (PMC8179019; doi:10.1039/d0sc05311b)
Supplement: SC-012-D0SC05311B-s001 [file SC-012-D0SC05311B-s001.pdf]

Electronic Supplementary Material (ESI) for Chemical Science.
This journal is © The Royal Society of Chemistry 2020

Supporting Information

Impact of the Macrocyclic Structure and Dynamic Solvent Effect on the Reactivity of a Localised Singlet Diradicaloid with π-Single Bonding Character

Zhe Wang,[a] Rikuo Akisaka,[a] Sohshi Yabumoto,[b] Tatsuo Nakagawa,[b] Sayaka Hatano,[a] and Manabu Abe*[a,c]

[a]Department of Chemistry, Graduate School of Science, Hiroshima University, 1-3-1 Kagamiyama, Higashi-Hiroshima, Hiroshima 739-8526, Japan

[b]Unisoku Co., Ltd., 2-4-3 Kasugano, Hirakata, Osaka 573-0131, Japan

[c]Hiroshima University Research Center for Photo-Drug-Delivery-Systems (HiU-P-DDS), Hiroshima University, 1-3-1 Kagamiyama, Higashi-Hiroshima, Hiroshima 739-8526, Japan

*E-mail: mabe@hiroshima-u.ac.jp

Table of Contents

1. Experimental Section 1

2. Spectral Data 6

3. X-ray Crystallographic Structure of AZ3b 9

4. Photolysis of AZ2b at 298 K 11

5. Low Temperature In-situ NMR Measurement Setup 11

6. Mass Spectra of Oxygenated Products 12

7. Decay of EPR Signal at 5−80 K Under Dark 13

8. Time-resolved Transient Absorption Spectroscopy 14

9. Correlation of $E_T(30)$ and Dielectric Constant ε_r on Singlet Diradical 17

10. Computational Details 18

11. References 92

1. Experimental Section

All commercially available reagents were purchased from TCI, Wako, Sigma Aldrich or Oakwood Chemical and were used without further purification. Dry solvents for spectroscopy analysis were purchased from commercial suppliers. NMR spectra were recorded on a Bruker Ascend 400 (^{1}H NMR: 400 MHz, ^{13}C NMR: 100 MHz) spectrometer at 298 K and referenced to the residual solvent peak. Coupling constants (*J*) are denoted in Hz and chemical shifts (δ) in ppm. The abbreviations s, d, t, dd, dt, td and m stand for the resonance multiplicities singlet, doublet, triplet, doublet of doublets, doublet of triplets, triplet of doublets and multiplet, respectively. Mass spectrometric data were measured with Thermo Fisher Scientific LTQ Orbitrap XL. UV-vis spectra were recorded on a SHIMADZU UV-3600 Plus spectrometer. The spectra were collected at room temperature using a slit width of 1 nm with middle scan rate. The excitation source for sub-microsecond laser flash photolysis was a tunable Nd:YAG minilite laser at 355 nm. The monitoring system consisted of a 150 W xenon arc lamp as light source, a Unisoku MD200 monochromator detection and a photomultiplier. The temperature was controlled by Unisoku CoolSpek USP-203-B. Sub-nanosecond transient absorption measurements were conducted with Unisoku PicoTAS system in 2 mm cuvette. The excitation source was a passive Q-SWITCH microchip laser at 355 nm. Recycling chromatographic separation in GPC were performed by LC-9210, Japan Analytical Industry Co., Ltd. with chloroform as mobile phase.

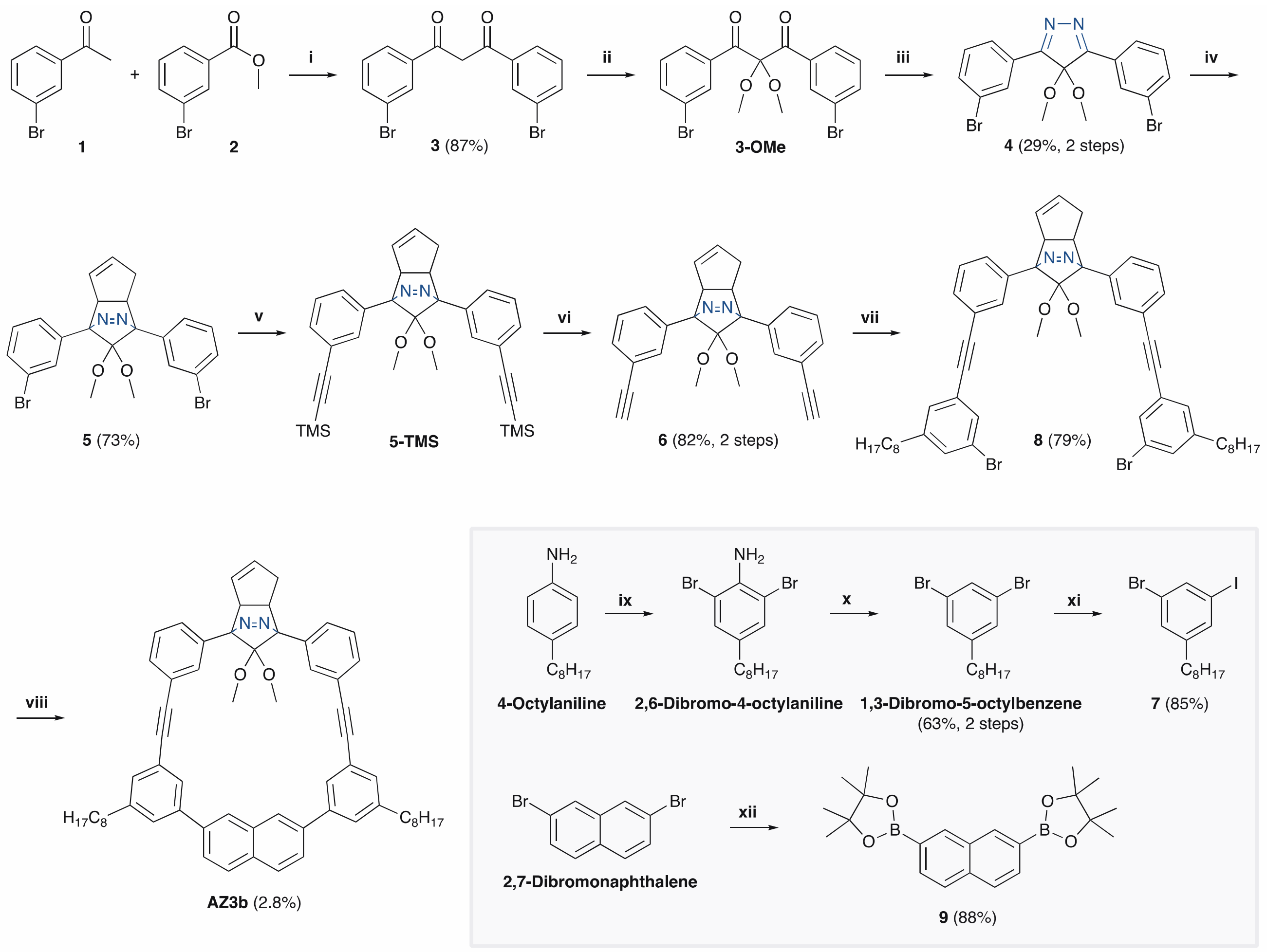

Scheme S1. Overview of synthesis.

(i) A suspension of sodium hydride (4.97 g, 60%, 124.17 mmol, 2 equiv.) in dry THF (100 mL) was stirred at room temperature for 30 minutes in a side-arm round flask under nitrogen atmosphere. After cooling to 0°C, 3-bromoacetophenone (**1**, 8.00 mL, 60.29 mmol, 1 equiv.) and a solution of methyl 3-bromobenzoate (**2**, 12.71 g, 59.10 mmol, 1 equiv.) in THF (20 mL) were added to the stirred solution. The reaction mixture was stirred at 0°C for 5 h and at room temperature for additional 14.5 h. The reaction was quenched by pouring into a mixture of crashed ice and 1 mol L^{-1} hydrochloric acid solution. The suspension was filtered through Büchner funnel, and the organic product was washed with hexane to give **3** (19.58 g, 51.25 mmol, 87%) as white solid. **^{1}H NMR** (400 MHz, $CDCl_3$): δ = 8.12 (s, 2H), 7.91 (d, *J* = 7.83 Hz, 2H), 7.69 (d, *J* = 8.01 Hz, 2H), 7.38 (dd, *J* = 7.95, 7.89 Hz, 2H), 6.76 (s, 1H). **^{13}C NMR** (100 MHz, $CDCl_3$): δ = 184.41, 137.26, 135.49, 130.29, 130.25, 125.78, 123.02, 93.44. **HRMS (ESI)** calculated for $C_{15}H_{10}O_2Br_2Na$ $[M+Na]^+$ = 404.89193, found 404.89203.

(ii) A two-neck flask equipped with a condenser was charged with **3** (22.23 g, 58.19 mmol, 1 equiv.), diphenyl diselenide (9.08 g, 29.09 mmol, 0.5 equiv.) and ammonium persulfate (26.55 g, 116.35 mmol, 2 equiv.) under nitrogen atmosphere. And the solids were mixed with methanol (220 mL). The reaction mixture was refluxed at 75°C for 3.5 h and then allowed to cool down to room temperature. Water was added to quench the reaction until all of the ammonium persulfate had dissolved, and the organic product was extracted with $CHCl_3$. The combined organic layers were dried over Na_2SO_4 and filtered. Removal of solvent under reduced pressure afforded **3-OMe** in a mixture as a dark orange oil, which was used for the next step without further purification since **3-OMe** is labile in silica gel.

(iii) Hydrazine monohydrate (3.20 mL) was slowly added to the solution of **3-OMe** (10.49 g, crude) in $CHCl_3$ (100 mL) while stirring under nitrogen atmosphere. The reaction mixture was refluxed at 70°C for 16 h and saturated $NaHCO_3$ aqueous solution was added to quench the reaction at room temperature. After stirring at room temperature for 30 minutes, the resulting mixture was extracted with $CHCl_3$. The combined organic layers were washed with brine, dried over $MgSO_4$ and filtered. The solvent was removed *in vacuo*. The residue was purified by silica gel column chromatography (hexane/dichloromethane = 2:1) to give **4** (2.85 g, 6.01 mmol, 29% of 2 steps) as yellow solid. **^{1}H NMR** (400 MHz, $CDCl_3$): δ = 8.41 (s, 2H), 8.19 (d, *J* = 7.91 Hz, 2H), 7.70 (d, *J* = 8.09 Hz, 2H), 7.40 (dd, *J* = 8.00, 7.92 Hz, 2H), 3.07 (s, 6H). **^{13}C NMR** (100 MHz, $CDCl_3$): δ = 166.06, 135.48, 130.64, 130.50, 129.34, 126.32, 123.26, 117.08, 52.12. **HRMS (ESI)** calculated for $C_{17}H_{14}N_2O_2Br_2Na$ $[M+Na]^+$ = 460.92938, found 460.92923.

(iv) Under exclusion of light, a side-arm round flask was charged with the solution of **4** (3.01 g, 6.85 mmol, 1 equiv.) in CH_2Cl_2 (100 mL) and cyclopentadiene (11.53 mL, 137.80 mmol, 20 equiv.) under nitrogen atmosphere. The reaction mixture was cooled to 0°C and trifluoroacetic acid (0.10 mL, 1.34 mmol, 0.2 eq.) was added to the reaction mixture while stirring. After stirring at 0°C for additional 1.5 h, saturated $NaHCO_3$ aqueous solution was added for neutralization. The organic product was extracted by CH_2Cl_2 and the combined organic layers were washed with brine, dried over $MgSO_4$ and filtered. The solvent was removed *in vacuo*. The residue was washed with hexane to give **5** (2.52 g, 5.01 mmol, 73%) as white solid. **^{1}H NMR** (400 MHz, $CDCl_3$): δ = 8.13 (s, 1H), 8.04 (s, 1H), 7.92 (d, *J* = 7.72 Hz, 1H), 7.82 (d, *J* = 7.72 Hz, 1H), 7.54 (t, *J* = 7.38 Hz, 2H), 7.34 (td, *J* = 7.96, 2.67 Hz, 2H), 5.61–5.56 (m,

1H), 5.51–5.46 (m, 1H), 4.15–4.09 (m, 1H), 3.70–3.62 (m, 1H), 3.00 (s, 3H), 2.71 (s, 3H), 2.41–2.38 (m, 1H), 2.34–2.15 (m, 1H). **^{13}C NMR** (100 MHz, $CDCl_3$): δ = 138.43, 138.25, 134.60, 131.79, 131.33, 131.18, 131.04, 130.07, 130.02, 127.27, 126.55, 126.12, 122.80, 122.73, 118.39, 94.00, 92.24, 57.26, 52.34, 51.99, 42.40, 32.16. **HRMS (ESI)** calculated for $C_{22}H_{20}N_2O_2Br_2Na$ $[M+Na]^+$ = 524.97837, found 524.97839.

(v) Under exclusion of light, a side-arm round flask with a condenser was charged with **5** (0.76 g, 1.51 mmol, 1 equiv.), tetrakis(triphenyl-phosphine)palladium(0) (0.18 g, 0.16 mmol, 0.1 equiv.) and copper(I) iodide (0.04 g, 0.21 mmol, 0.1 equiv.) under nitrogen atmosphere. Anhydrous THF (20 mL), anhydrous triethylamine (5 mL) and trimethylsilylacetylene (2.25 mL, 16.26 mmol, 10 equiv.) were added into flask via a syringe. The reaction mixture was refluxed at 60°C for 43.5 h in the absence of light. After cooling to room temperature, the reaction mixture was filtered through a pad of celite and concentrated. The residue was redissolved in $CHCl_3$, organic layers were washed with saturated NH_4Cl aqueous solution and dried over Na_2SO_4. After filtering, the solvent was removed under reduced pressure. The residue was purified through a short silica gel column (hexane/ethyl acetate = 5:1) to afford **5-TMS** in mixture, hence **5-TMS** was directly used for the next step reaction.

(vi) Under exclusion of light, the solution of **5-TMS** (4.23 g, mixture) in THF (50 mL), potassium carbonate (0.32 g) and methanol (90 mL) were charged into a round-bottom flask. The reaction mixture was stirred at room temperature for 16.5 h. After filtering through a pad of celite, the solvent was removed *in vacuo*. Water was added to the resulting crude and the organic product was extracted with ethyl acetate. The combined organic layers were washed with brine, dried over Na_2SO_4, filtered and concentrated. The residue was purified by silica gel column chromatography (hexane/ethyl acetate = 10:1) to give **6** (1.98 g, 5.01 mmol, 82% of 2 steps) as a white solid. **^{1}H NMR** (400 MHz, $CDCl_3$): δ = 8.10 (s, 1H), 8.02–7.97 (m, 2H), 7.91 (d, J = 7.58 Hz, 1H), 7.53 (dd, J = 6.89, 7.35 Hz, 2H), 7.43 (td, J = 7.81, 2.99 Hz, 2H), 5.61–5.55 (m, 1H), 5.51–5.46 (m, 1H), 4.18–4.12 (m, 1H), 3.74–3.65 (m, 1H), 3.11 (s, 2H), 2.98 (s, 3H), 2.69 (s, 3H), 2.41–2.30 (m, 1H), 2.25–2.14 (m, 1H). **^{13}C NMR** (100 MHz, $CDCl_3$): δ = 136.53, 136.35, 134.49, 132.23, 131.87, 131.71, 131.52, 129.42, 128.64, 128.53, 128.49, 126.27, 122.35, 122.30, 118.33, 94.08, 92.35, 83.71, 83.69, 57.12, 52.29, 52.24, 51.93, 51.90, 42.28, 32.18. **HRMS (ESI)** calculated for $C_{26}H_{22}N_2O_2Na$ $[M+Na]^+$ = 417.15735, found 417.15695.

(vii) Under exclusion of light, a side-arm round flask with a condenser was charged with **6** (1.41 g, 3.57 mmol, 1 equiv.), tetrakis(triphenyl-phosphine)palladium(0) (0.22 g, 0.19 mmol, 5% equiv.) and copper(I) iodide (0.06 g, 0.31 mmol, 0.1 equiv.) under nitrogen atmosphere. Anhydrous THF (36 mL), anhydrous triethylamine (13 mL) and 1-bromo-3-iodo-5-*n*-octylbenzene (**7**, 2.50 mL, 9.49 mmol, 2.5 equiv.) were added into flask via a syringe. The reaction mixture was refluxed at 60°C for 39.5 h in the absence of light. After cooling to room temperature, the reaction mixture was filtered through a pad of celite and concentrated. The residue was redissolved in $CHCl_3$, organic layers were sequentially washed with saturated NH_4Cl aqueous solution and water before dried over Na_2SO_4. After filtering, the solvent was removed under reduced pressure. The residue was purified by silica gel column chromatography (hexane/ethyl acetate = 10:1) to afford **8** (2.61 g, 2.82 mmol, 79%) as an orange oil. **^{1}H NMR** (400 MHz, $CDCl_3$): δ = 8.14 (s, 1H), 8.05 (s, 1H), 7.99 (d, J = 7.73 Hz, 1H), 7.91 (d, J = 8.19 Hz, 1H), 7.58–7.44 (m, 6H), 7.34–7.24 (m,

4H), 5.63–5.57 (m, 1H), 5.55–5.49 (m, 1H), 4.23–4.16 (m, 1H), 3.78–3.69 (m, 1H), 3.03 (s, 3H), 2.73 (s, 3H), 2.58 (t, J = 8.24 Hz, 4H), 2.45–2.33 (m, 1H), 2.29–2.18 (m, 1H), 1.68–1.55 (m, 4H), 1.40–1.20 (m, 20H), 0.89 (t, J = 7.37 Hz, 6H). **^{13}C NMR** (100 MHz, $CDCl_3$): δ = 145.19, 136.57, 136.39, 134.54, 131.84, 131.63, 131.57, 131.37, 131.20, 131.12, 130.33, 129.10, 128.65, 128.61, 128.35, 126.34, 124.83, 123.12, 123.08, 122.00, 118.37, 94.21, 92.47, 90.27, 90.25, 88.35, 88.32, 57.12, 52.28, 51.98, 42.28, 35.58, 32.25, 31.87, 31.10, 29.41, 29.21, 29.19, 22.67, 14.11. **HRMS (ESI)** calculated for $C_{54}H_{60}N_2O_2Br_2Na$ $[M+Na]^+$ = 951.28888, found 951.28933.

(viii) Under exclusion of light, **8** (660.0 mg, 0.71 mmol, 1 equiv.), palladium(II) acetate (34.2 mg, 0.15 mmol, 0.2 equiv.), **9** (269.1 mg, 0.71 mmol, 1 equiv.), potassium phosphate (603.4 mg, 2.84 mmol, 4 equiv.), 2-dicyclohexylphosphino-2',6'-dimethoxybiphenyl (**SPhos**, 117.2 mg, 0.29 mmol, 0.4 equiv.) were charged into a side-arm flask with a condenser. Degassed THF (100 mL) and water (15 mL) were added into the flask under nitrogen atmosphere. The reaction mixture was refluxed at 80°C for 21 h in the absence of light. After cooling to room temperature, the reaction mixture was filtered through a pad of celite and concentrated. The residue was redissolved in $CHCl_3$, organic layers were washed with brine before and over Na_2SO_4. After filtering, the solvent was removed *in vacuo*. The residue was purified by silica gel column chromatography (hexane/dichloromethane = 1:2) to afford **AZ3b** (17.0 mg, 0.02 mmol, 2.8%) as a white solid. **^{1}H NMR** (400 MHz, C_6D_6): δ = 9.08 (d, J = 7.40 Hz, 2H), 8.27 (d, J = 5.16 Hz, 2H), 7.75 (d, J = 8.46 Hz, 2H), 7.72–7.65 (m, 4H), 7.63 (d, J = 8.17 Hz, 2H), 7.55 (s, 2H), 7.51 (s, 2H), 7.40 (d, J = 7.80 Hz, 1H), 7.31 (d, J = 8.08 Hz, 1H), 7.21–7.17 (m, 2H), 5.53–5.46 (m, 1H), 5.36–5.31 (m, 1H), 4.18–4.10 (m, 1H), 3.58–3.48 (m, 1H), 2.58 (s, 3H), 2.57 (t, J = 8.40 Hz, 4H), 2.32–2.21 (m, 1H), 2.14 (s, 3H), 2.14–2.05 (m, 1H), 1.66–1.53 (m, 4H), 1.36–1.20 (m, 20H), 0.96–0.85 (m, 6H). **^{13}C NMR** (400 MHz, $CDCl_3$): δ = 144.08, 141.21, 137.80, 137.34, 136.98, 136.92, 134.87, 134.61, 132.58, 132.35, 129.99, 129.93, 129.15, 128.65, 128.59, 128.54, 128.50, 126.90, 126.70, 120.50, 124.91, 124.80, 124.31, 119.52, 95.20, 93.70, 91.80, 90.88, 54.81, 52.15, 51.04, 41.13, 36.46, 32.35, 31.87, 29.95, 29.80, 29.71, 23.13, 14.40. **HRMS (ESI)** calculated for $C_{64}H_{66}N_2O_2Na$ $[M+Na]^+$ = 917.50165, found 917.50177.

(ix) In a round-bottom flask, 4-*n*-octylaniline (5.00 mL, 21.87 mmol, 1 equiv.) was dissolved in *N*,*N*-dimethylformamide (20 mL) and iced at 0°C. The solution of *N*-bromosuccinimide (10.00 g, 56.19 mmol, 2.5 equiv.) in *N*,*N*-dimethyl-formamide (30 mL) was added into flask dropwise. The reaction mixture was stirred at 0°C for 30 minutes and at room temperature for additional 3 h. Then, water was added into the resulting mixture and the organic product was extracted with ethyl acetate. The combined organic layers were washed with saturated $Na_2S_2O_3$ aqueous solution, dried over $MgSO_4$ and filtered. Removal of solvent under reduced pressure gave 2,6-dibromo-4-*n*-octylaniline as a red solid in mixture (11.62 g), which was used for next step without further purification.

(x) A solution of 2,6-dibromo-4-*n*-octylaniline (11.62 g, crude) in ethanol (250 mL), concentrated sulfuric acid (30 mL) were charged into a side-arm flask with a condenser. Sodium nitrite (6.62 g) was slowly added into the mixture little by little while stirring. After refluxing at 70°C for 2 h, ice water was added to quench the reaction. The organic product was extracted by dichloromethane, and the organic layers were dried over Na_2SO_4, filtered and removed solvent under reduced pressure. The residue was purified by silica gel column chromatography (hexane only) to

afford 1,3-dibromo-5-*n*-octylbenzene (4.80 g, 13.78 mmol, 63% of 2 steps) as a colorless liquid. **^{1}H NMR** (400 MHz, $CDCl_3$): δ = 7.48 (s, 1H), 7.25 (s, 2H), 2.54 (t, *J* = 7.63 Hz, 2H), 1.63–1.53 (m, 2H), 1.36–1.20 (m, 10H), 0.88 (t, *J* = 7.09 Hz, 3H). **HRMS (GC-EI)** calculated for $C_{14}H_{20}Br_2$ $[M]^+$ = 347.99118, found 347.99081.

(xi) At –78°C, a 2.3 M solution of *n*-BuLi in hexane (17.00 mL, 39.1 mmol, 1.1 equiv.) was added slowly to a solution of 1,3-dibromo-5-*n*-octylbenzene (12.14 g, 34.9 mmol, 1 equiv.) in THF (250 mL) via a syringe under nitrogen atmosphere. The reaction mixture was stirred at -78°C for 40 minutes, then a solution of 1,2-diiodoethane (11.83 g, 41.97 mmol, 1.2 equiv.) in THF (60 mL) was slowly added and the reaction mixture was allowed to warm to room temperature and stirred for additional 4 h. Then, solvent was removed *in vacuo*, and the residue was redissolved in diethyl ether. The solution was sequentially washed with saturated Na_2SO_3 aqueous solution, saturated $NaHCO_3$ aqueous solution and water before dried over Na_2SO_4 and concentrated *in vacuo*. The crude was purified by silica gel column chromatography (hexane only) to provide **7** (11.72 g, 29.67 mmol, 85%) as a colorless liquid. **^{1}H NMR** (400 MHz, $CDCl_3$): δ = 7.66 (s, 1H), 7.45 (s, 1H), 7.28 (s, 1H), 2.51 (t, *J* = 8.09 Hz, 2H), 1.62–1.51 (m, 2H), 1.34–1.19 (m, 10H), 0.88 (t, *J* = 6.86 Hz, 3H). **HRMS (GC-EI)** calculated for $C_{14}H_{20}BrI$ $[M]^+$ = 393.97931, found 393.97853.

(xii) A side-arm round flask with a condenser was charged with 2,7-dibromonaphthalene (1.23 g, 4.30 mmol, 1 equiv.), [1,1′-bis(diphenylphosphino)ferrocene]dichloropalladium(II) (0.16 g, 0.22 mmol, 5% equiv.), bis(pinacolato)diboron (3.27g, 12.88 mmol, 3 equiv.), potassium acetate (2.54 g, 25.91 mmol, 6 equiv.) and 1,4-dioxane (10 mL) under nitrogen atmosphere. The reaction mixture was stirred at 75°C for 20 h. After cooling to room temperature, the mixture was filtered through a pad of celite and removed solvent *in vacuo*. The residue was purified by silica gel column chromatography (hexane/dichloromethane = 2:1) and gel permeation chromatography (chloroform only) to give **9** (1.44 g, 3.78 mmol, 88%) as a white solid. **^{1}H NMR** (400 MHz, $CDCl_3$): δ = 8.41 (s, 2H), 7.86 (d, *J* = 8.14 Hz, 2H), 7.80 (d, *J* = 8.14 Hz, 2H), 1.38 (s, 24H). **^{13}C NMR** (100 MHz, $CDCl_3$): δ = 137.11, 131.53, 126.78, 83.87, 25.02, 24.92. **HRMS (APCI)** calculated for $C_{22}H_{31}O_4B_2$ $[M+H]^+$ = 381.24030, found 381.24133.

2. Spectral Data

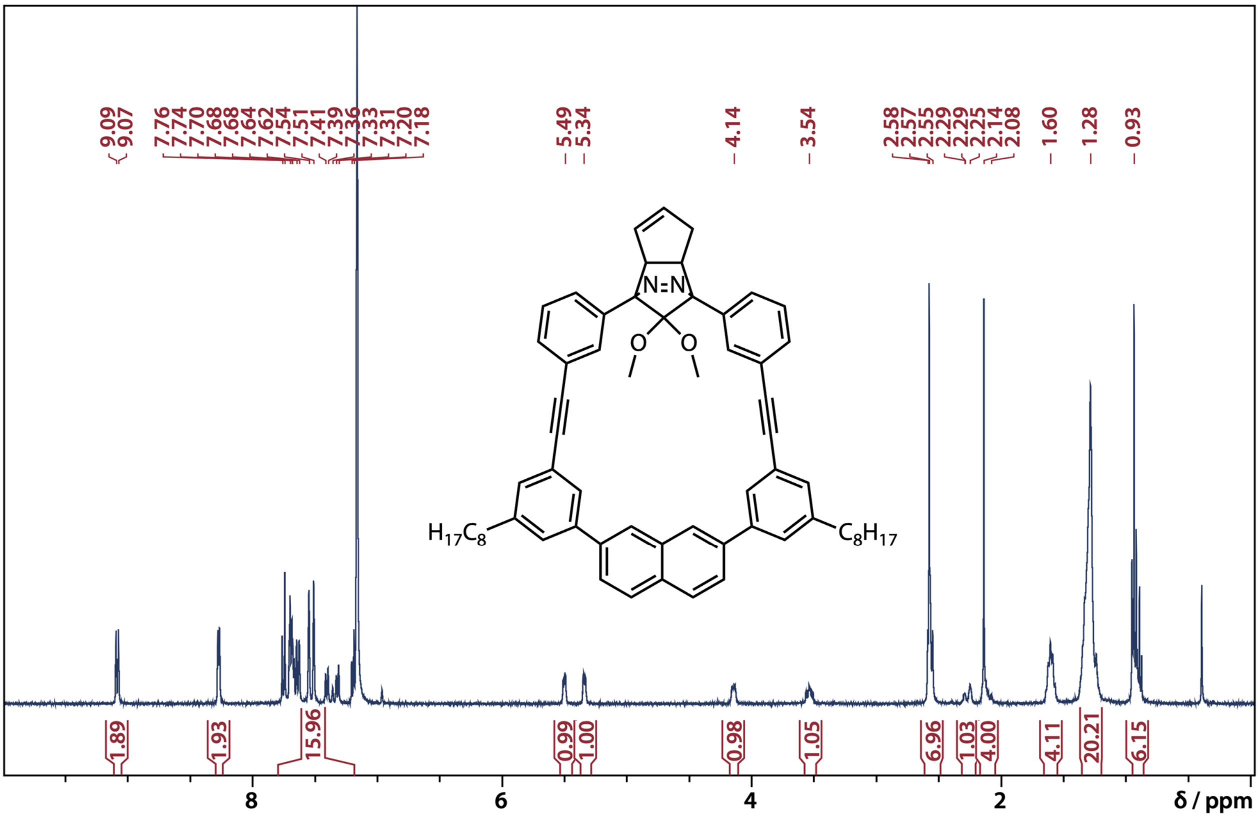

Figure S1. ^{1}H NMR spectrum of **AZ3b** (C_6D_6, 400 MHz).

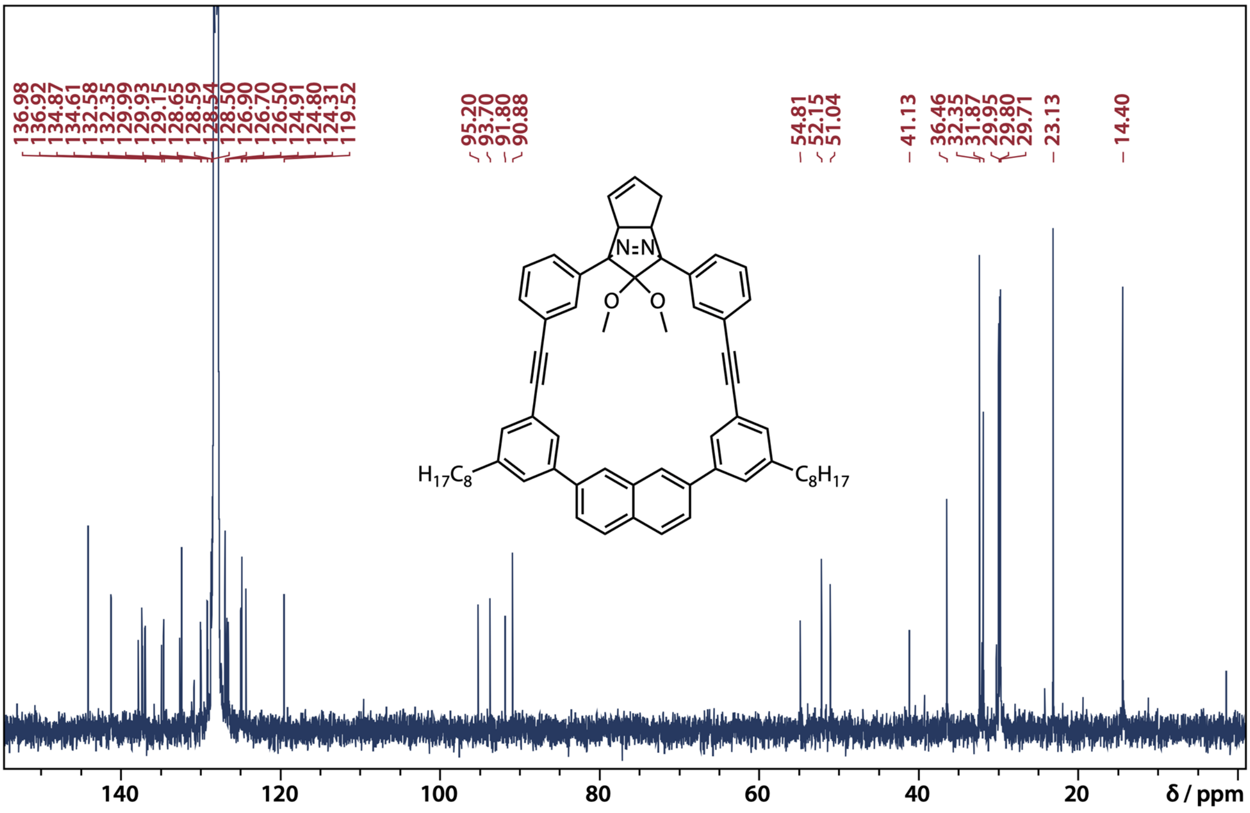

Figure S2. ^{13}C NMR spectrum of **AZ3b** (C_6D_6, 100 MHz).

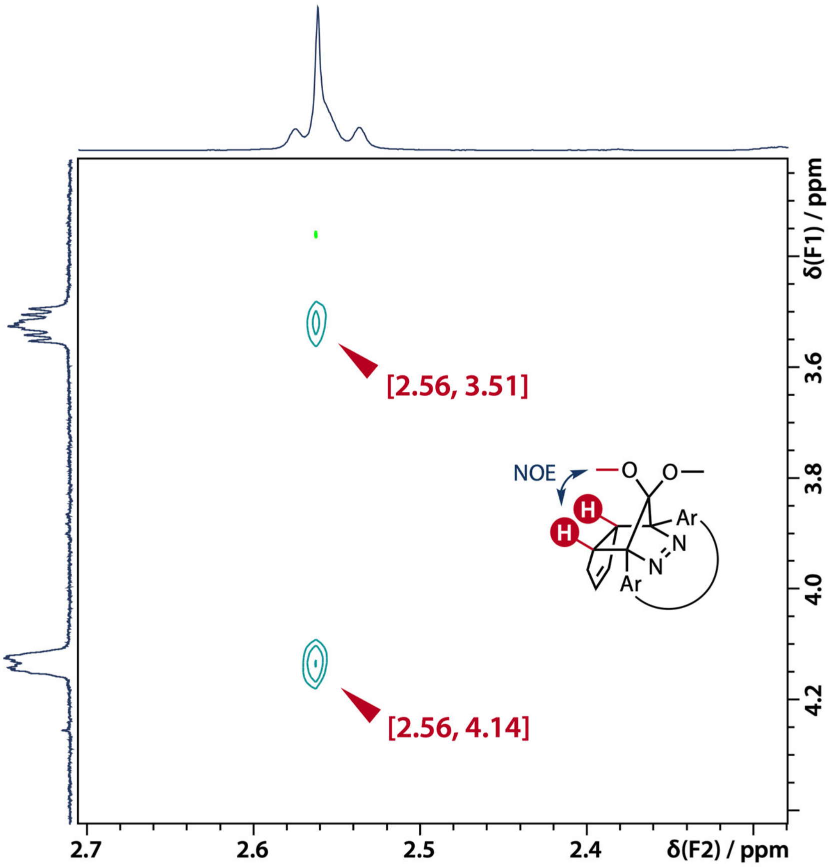

Figure S3. 2D NOESY NMR spectrum of **AZ3b** (C_6D_6, 400 MHz).

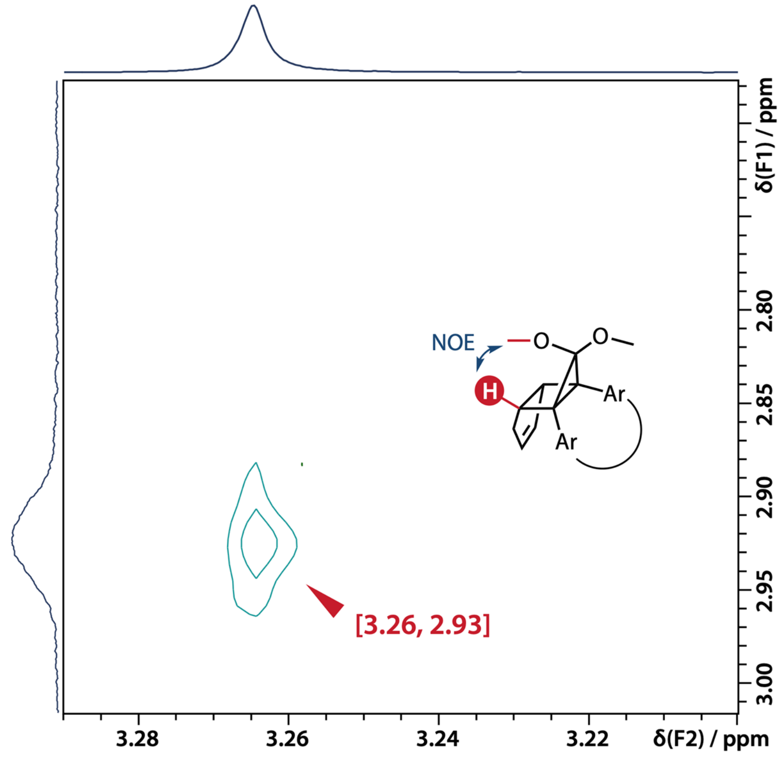

Figure S4. 2D NOESY NMR spectrum of *trans*-**CP3b** (C_6D_6, 400 MHz).

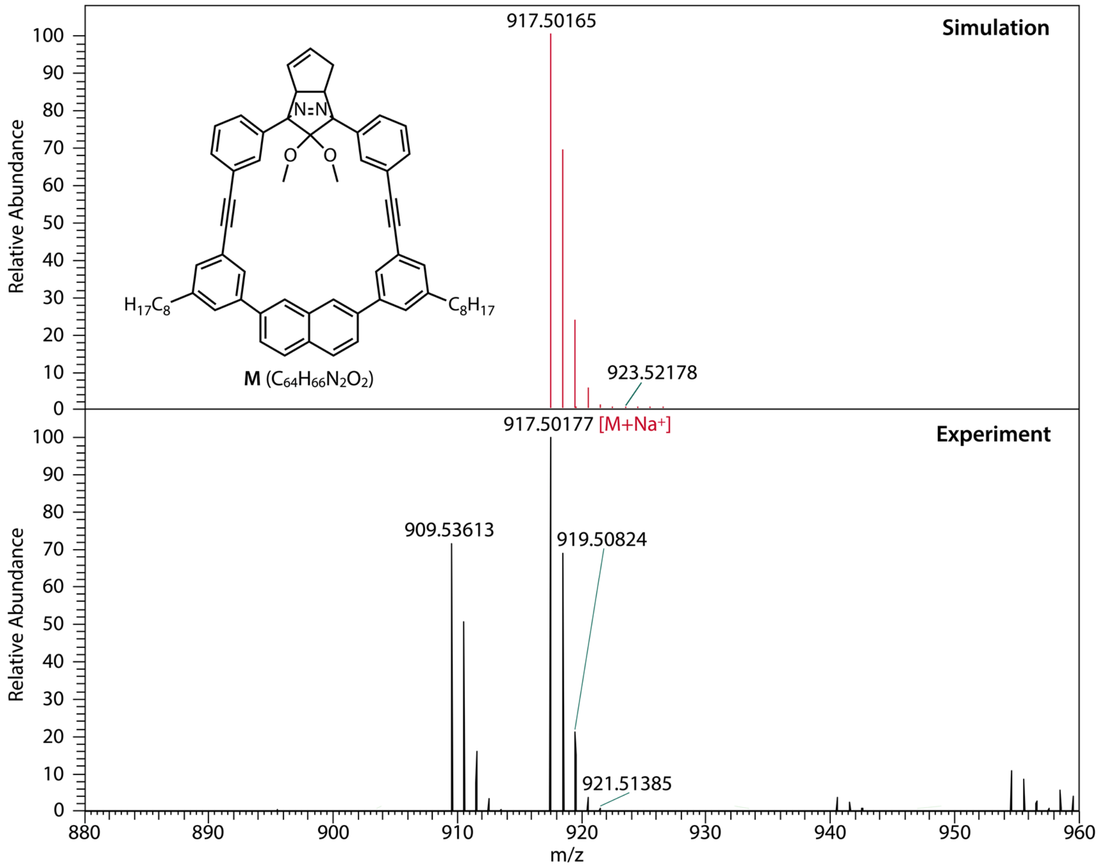

Figure S5. High resolution mass spectrum (ESI) of **AZ3b**.

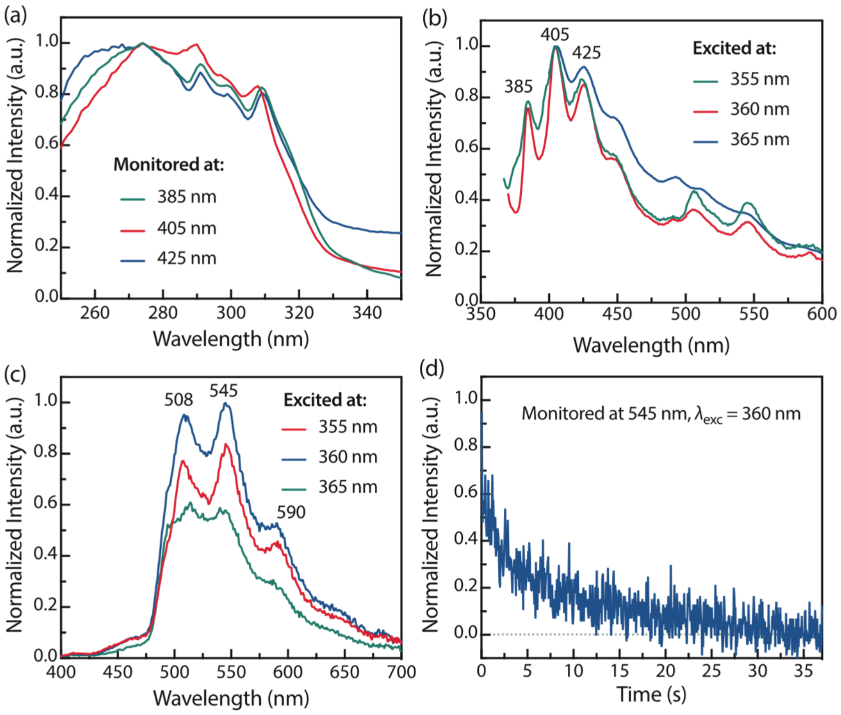

Figure S6. Excitation and emission spectra of **AZ3b** (0.37 mM) in MTHF matrix at 77 K. (a) Excitation spectra (λ_{exc} = 360 nm) monitored at 385, 405 and 425 nm; (b) Fluorescence spectra excited at 355, 360 and 365 nm; (c) Phosphorescence spectra excited at 355, 360 and 365 nm; (d) Time profile of phosphorescence at 545 nm (λ_{exc} = 360 nm).

3. X-ray Crystallographic Structure of AZ3b

Table S1. Crystal data and structure refinement for **AZ3b**.

Bond precision:	C-C = 0.0070 A		Wavelength=0.71073
Cell:	a=32.428(5)	b=24.305(3)	c=16.353(2)
	alpha=90	beta=112.760(2)	gamma=90
Temperature:	100 K		
	Calculated		Reported
Volume	11885(3)		11885(3)
Space group	C 2/c		C 1 2/c 1
Hall group	-C 2yc		-C 2yc
Moiety formula	C64 H66 N2 O2, 1.5(C6 H6), 0.5(C6 H4)		?
Sum formula	C76 H77 N2 O2		C76 H78 N2 O2
Mr	1050.40		1051.40
Dx,g cm-3	1.174		1.175
Z	8		8
Mu (mm-1)	0.069		0.069
F000	4504.0		4512.0
F000’	4505.66		
h,k,lmax	36,27,18		36,27,18
Nref	9084		9058
Tmin,Tmax	0.980,0.997		0.890,1.000
Tmin’	0.980		

Correction method= # Reported T Limits: Tmin=0.890 Tmax=1.000

AbsCorr = MULTI-SCAN

Data completeness= 0.997 Theta(max)= 23.770

R(reflections)= 0.0752(6106) wR2(reflections)= 0.2163(9058)

S = 1.033 Npar= 726

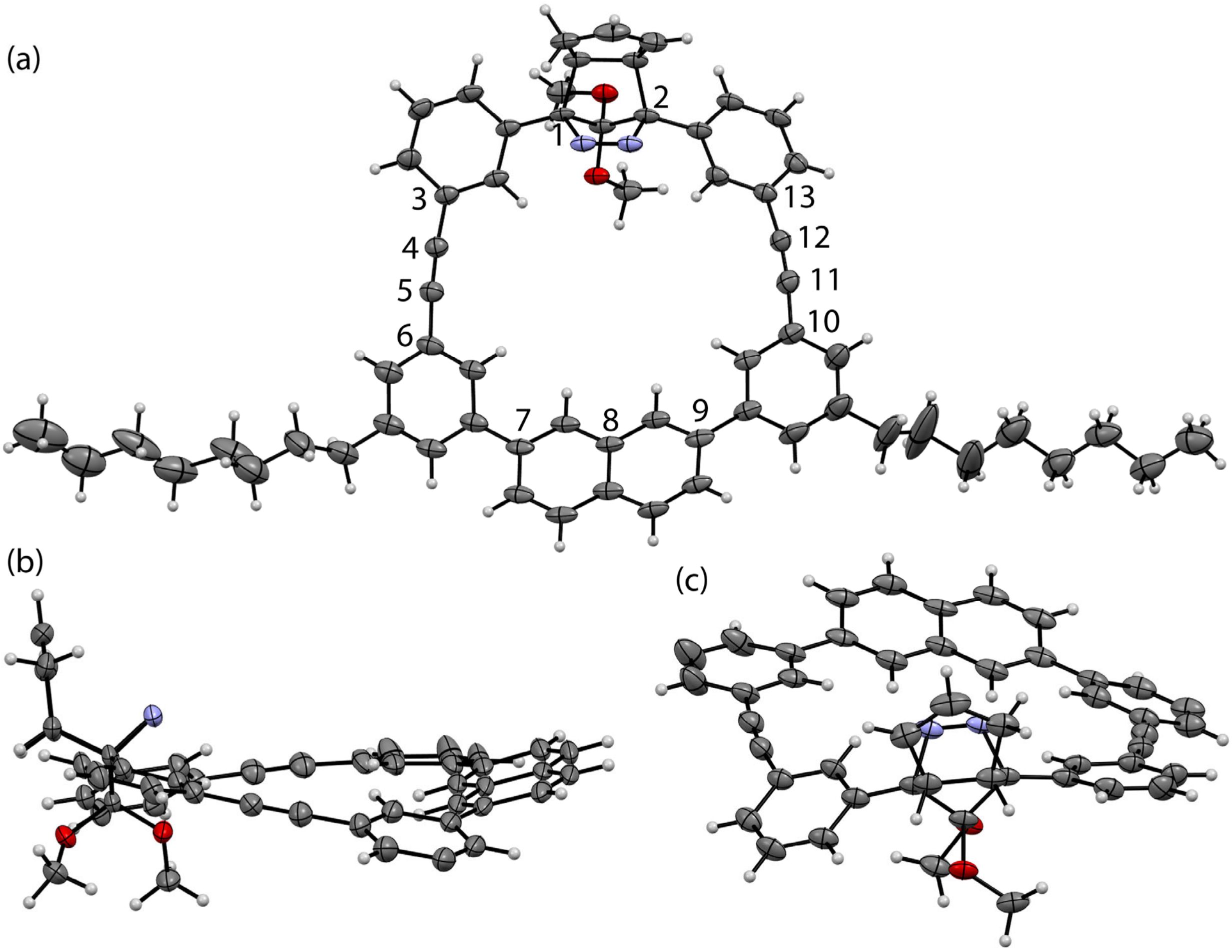

Figure S7. X-ray crystallographic structure of **AZ3b** in (a) top view, (b) side view and (c) front view, atomic displacement was observed at octyl chains. Octyl chains are omitted in side and front views.

Table S2. Experimental and computed geometry data at (R)ωB97X-D/6-31G(d) level of theory of **AZ3b**.

Entry		Experimental Data	Computed Data
1	C1-C2	2.263 Å	2.248 Å
2	C3-C4-C5	175.14°	175.61°
3	C4-C5-C6	175.31°	173.58°
4	C7-C8-C9	177.79°	176.09°
5	C10-C11-C12	172.67°	172.70°
6	C11-C12-C13	172.99°	174.88°

4. Photolysis of AZ2b at 298 K

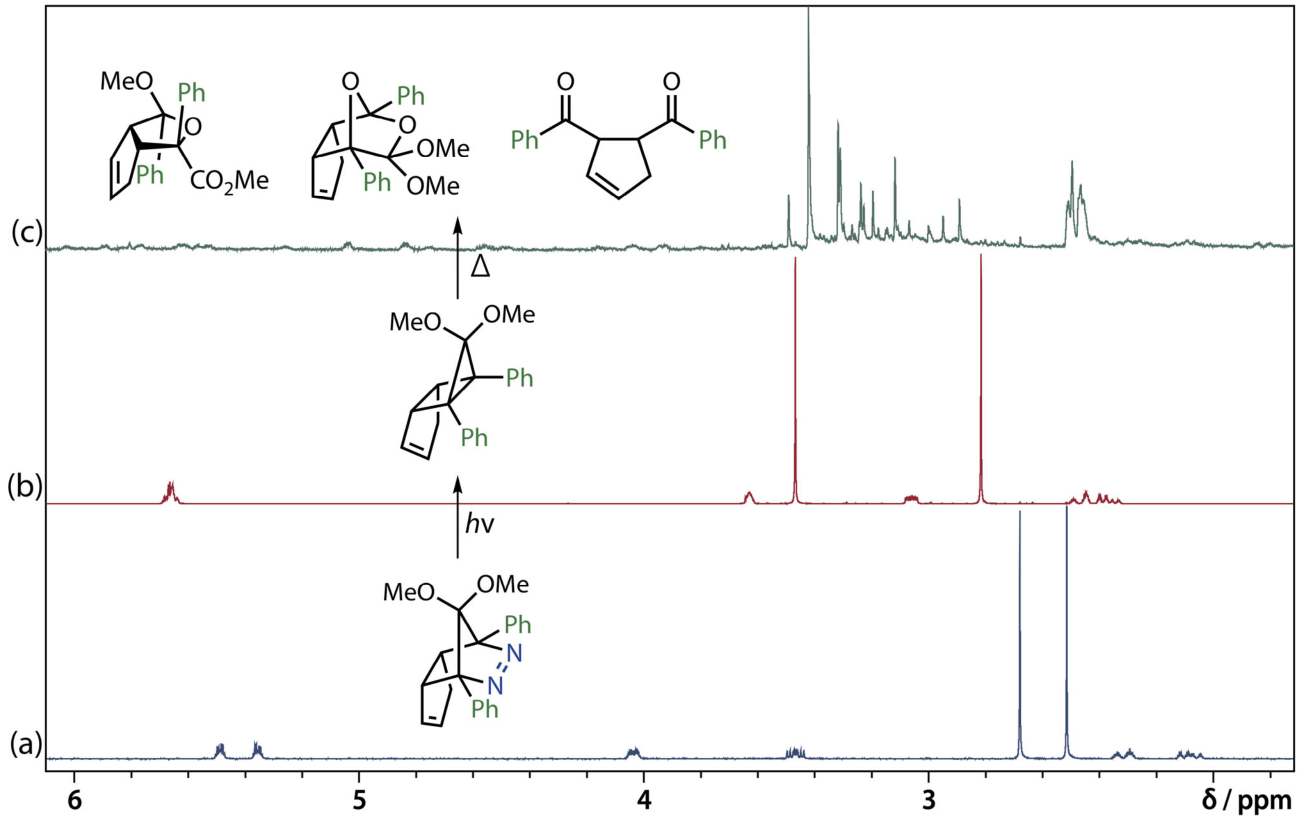

Figure S8. In-situ ^{1}H NMR (400 MHz) analysis of photoreaction of **AZ2b** in degassed C_6D_6, (a) ^{1}H NMR spectrum of **AZ2b** before irradiation; (b) ^{1}H NMR spectrum of *trans*-**CP2b** after irradiation with a 365 nm LED lamp for 120 s at 298 K under nitrogen atmosphere; (c) ^{1}H NMR spectrum of decomposed *trans*-**CP2b** after heating at 100°C for 5 h under air condition.

5. Low Temperature In-situ NMR Measurement Setup

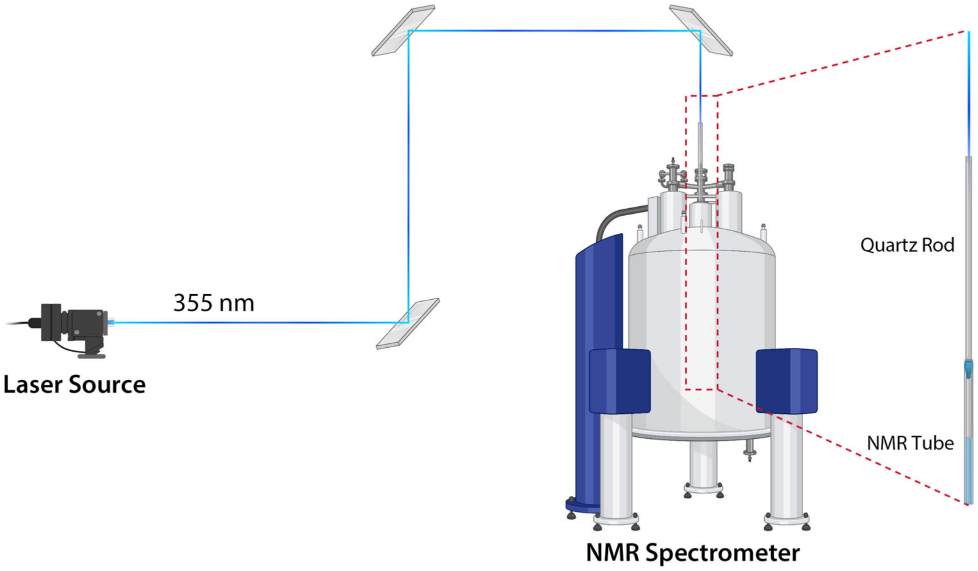

Figure S9. Low temperature in-situ NMR measurement setup.

6. Mass Spectra of Oxygenated Products

Oxygenated products **10**–**12** were isolated by PTLC with a mixture solvent of hexane/dichloromethane = 1:1 (v/v), and identified by high resolution mass spectroscopy.

Table S3. R_f value of oxygenated products**10**–**12** and mass analysis results.

Compound	R_f (in 1:1 of hex./DCM)	Ionization Method	Detected Ions
**10**	0.56	ESI	$C_{63}H_{63}O_3$ [M–OCH_3]$^+$ and $C_{64}H_{66}O_4Na$ [M+Na]$^+$
**11**	0.68	ESI	$C_{64}H_{66}O_4Na$ [M+Na]$^+$
**12**	0.33	APCI	$C_{61}H_{61}O_2$ [M+H]$^+$

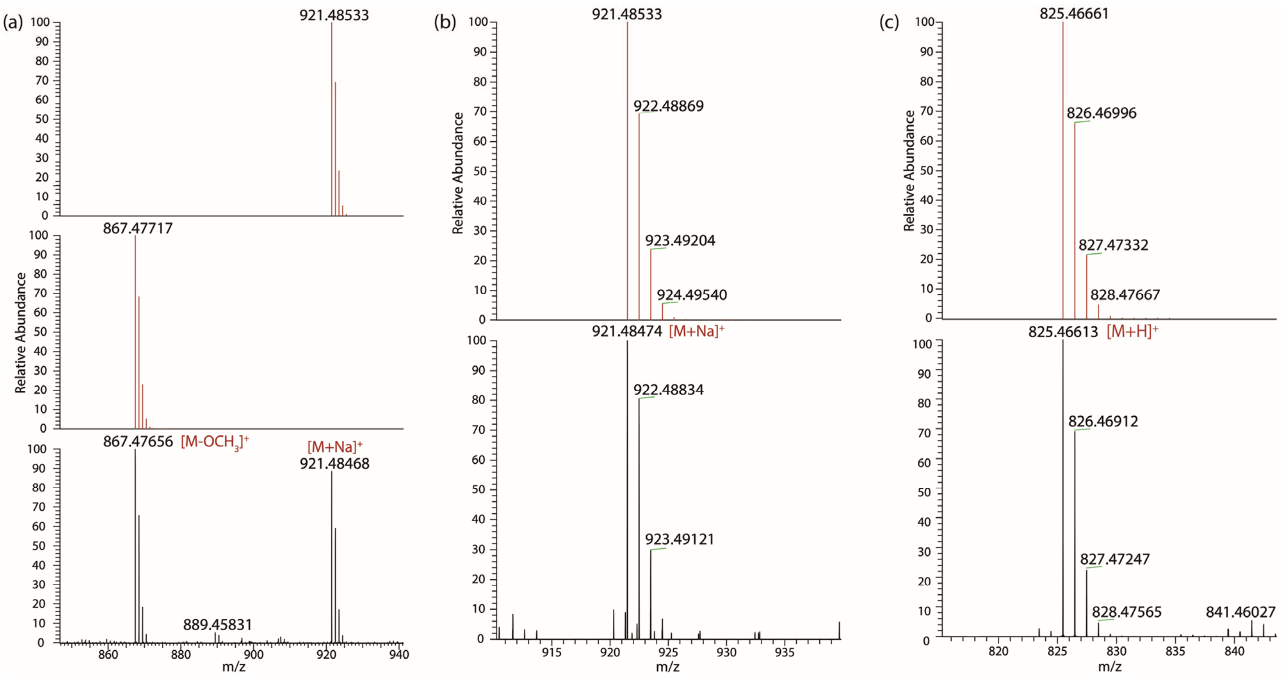

Figure S10. High resolution mass spectra of (a) **10** (ESI, found [M–OCH_3]$^+$ and [M+Na]$^+$), (b) **11** (ESI, found [M+Na]$^+$) and (c) **12** (APCI, found [M+H]$^+$).

7. Decay of EPR Signal at 5−80 K Under Dark

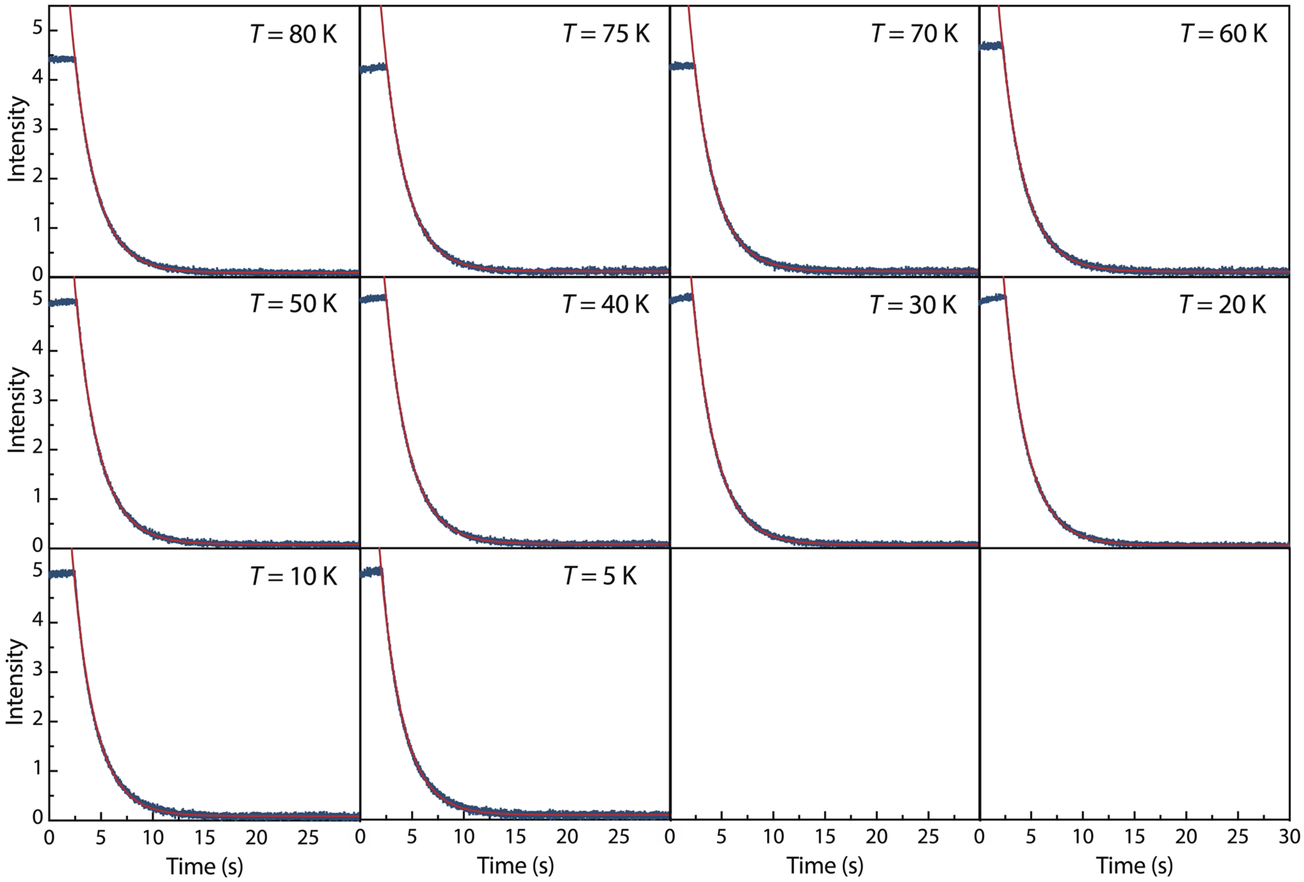

Figure S11. EPR time profile monitored at 1562 G at 5−80 K.

Table S4. Lifetime and decay rate constant of EPR signal at 1562 G under dark condition.

Entry	Temperature / K	τ_d / s	k_d / s^{-1}
1	5	2.196	0.4554
2	10	2.190	0.4566
3	20	2.253	0.4439
4	30	2.295	0.4356
5	40	2.285	0.4377
6	50	2.301	0.4346
7	60	2.285	0.4376
8	70	2.271	0.4404
9	75	2.232	0.4481
10	80	2.253	0.4439

8. Time-resolved Transient Absorption Spectroscopy

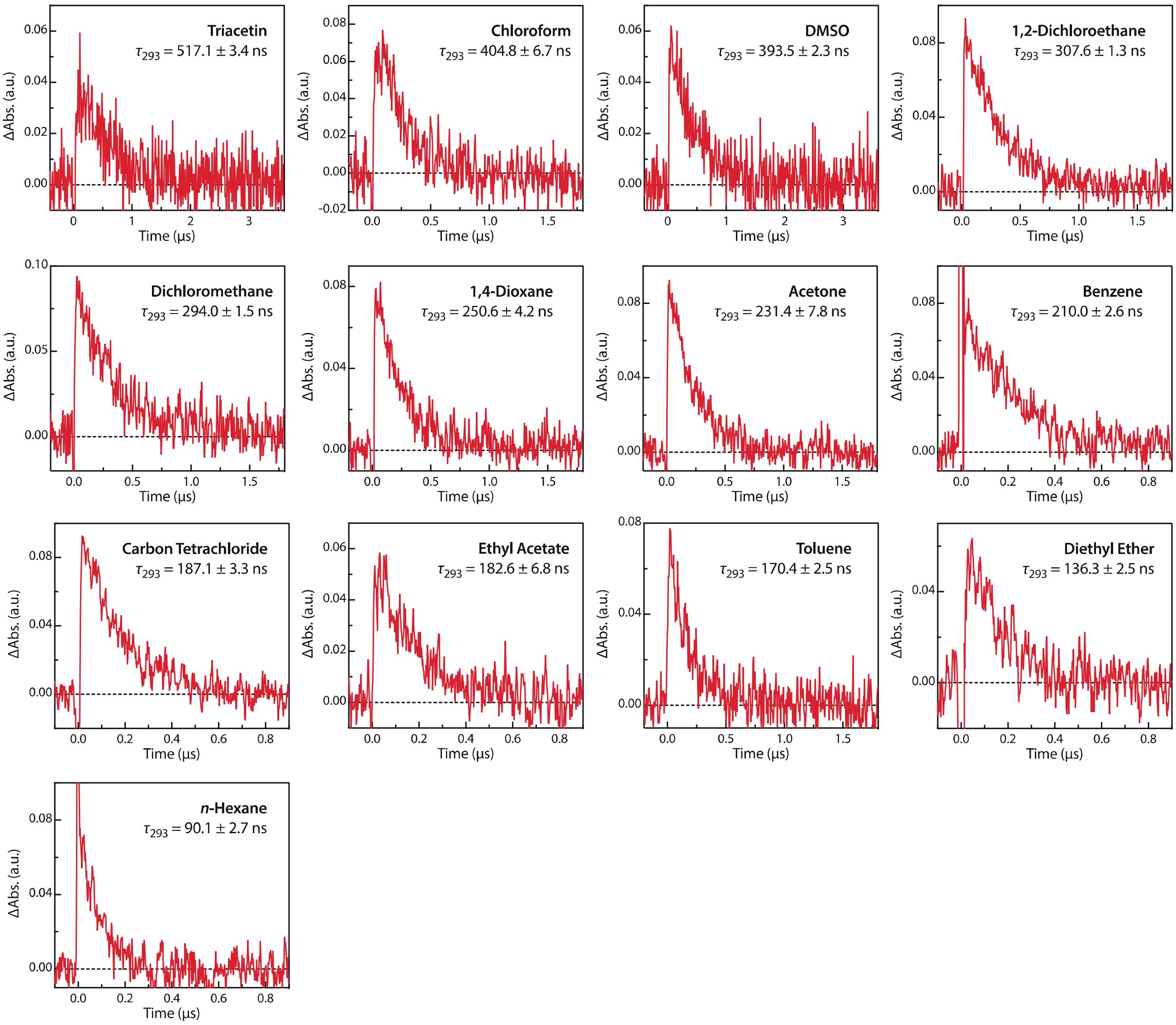

Figure S12. Decay profile monitored at 570−580 nm of S-**DR2b** at 293 K in triacetin, chloroform, DMSO, 1,2-dichloroethane, dichloromethane, 1,4-dioxane, acetone, benzene, carbon tetrachloride, ethyl acetate, toluene, diethyl ether and *n*-hexane. Lifetime are calculated from single-exponential decay model fitting.

Table S5. Activation parameters (E_a, log A, $\Delta H^‡$, $\Delta S^‡$, $\Delta G^‡_{293}$) of ring-closing processes for S-**DR2b** determined by Arrhenius and Eyring plots.

Entry	Solvent	$E_a{}^a$ / kJ mol^{-1}	log A^a / s^{-1}	$\Delta H^{‡b}$ / kJ mol^{-1}	$\Delta S^{‡b}$ / J mol^{-1} K^{-1}	$\Delta G^{‡b}_{293}$ / kJ mol^{-1}
1	Triacetin	51.9 ± 1.5	15.5 ± 0.4	49.3 ± 1.5	42.4 ± 4.9	37.0 ± 1.5
2	$CHCl_3$	34.1 ± 1.1	12.5 ± 0.2	31.7 ± 1.1	−13.7 ± 3.1	35.8 ± 1.1
3	DMSO	40.2 ± 1.2	13.5 ± 0.2	37.6 ± 1.2	4.7 ± 2.8	36.2 ± 1.2
4	CH_2ClCH_2Cl	28.8 ± 1.2	11.7 ± 0.2	26.4 ± 1.2	−29.9 ± 4.0	35.2 ± 1.2
5	CH_2Cl_2	32.9 ± 0.2	12.4 ± 0.1	30.7 ± 0.2	−15.3 ± 0.8	35.1 ± 0.2
6	1,4-Dioxane	34.5 ± 1.0	12.7 ± 0.2	31.9 ± 1.0	−10.0 ± 3.3	34.8 ± 1.0

Continued Table S5.

Entry	Solvent	$E_a{}^a$ / kJ mol^{-1}	log A^a / s^{-1}	$\Delta H^{\ddagger b}$ / kJ mol^{-1}	$\Delta S^{\ddagger b}$ / J mol^{-1} K^{-1}	$\Delta G^{\ddagger b}{}_{293}$ / kJ mol^{-1}
7	Acetone	37.8 ± 0.5	13.4 ± 0.1	35.4 ± 0.5	2.8 ± 1.9	34.6 ± 0.5
8	Benzene	30.5 ± 0.4	12.1 ± 0.1	28.0 ± 0.4	−21.5 ± 0.8	34.2 ± 0.8
9	CCl_4	33.1 ± 0.6	12.7 ± 0.1	30.7 ± 0.6	−11.2 ± 2.1	34.0 ± 0.6
10	EtOAc	32.7 ± 0.9	12.5 ± 0.2	30.3 ± 0.9	−14.0 ± 3.1	34.4 ± 0.9
11	Toluene	33.5 ± 1.0	12.7 ± 0.2	31.0 ± 1.1	−11.1 ± 3.6	34.3 ± 1.1
12	Ether	29.6 ± 0.5	12.1 ± 0.1	27.3 ± 0.5	−21.0 ± 1.8	33.4 ± 0.5
13	Hexane	29.3 ± 0.8	12.2 ± 0.2	26.9 ± 0.8	−18.7 ± 3.0	32.4 ± 0.8

Determined from [a] Arrhenius plots and [b] Eyring plots, respectively, with lifetime of S-**DR2b** at five temperatures between 253 and 333 K, errors are standard errors obtained from regression analysis.

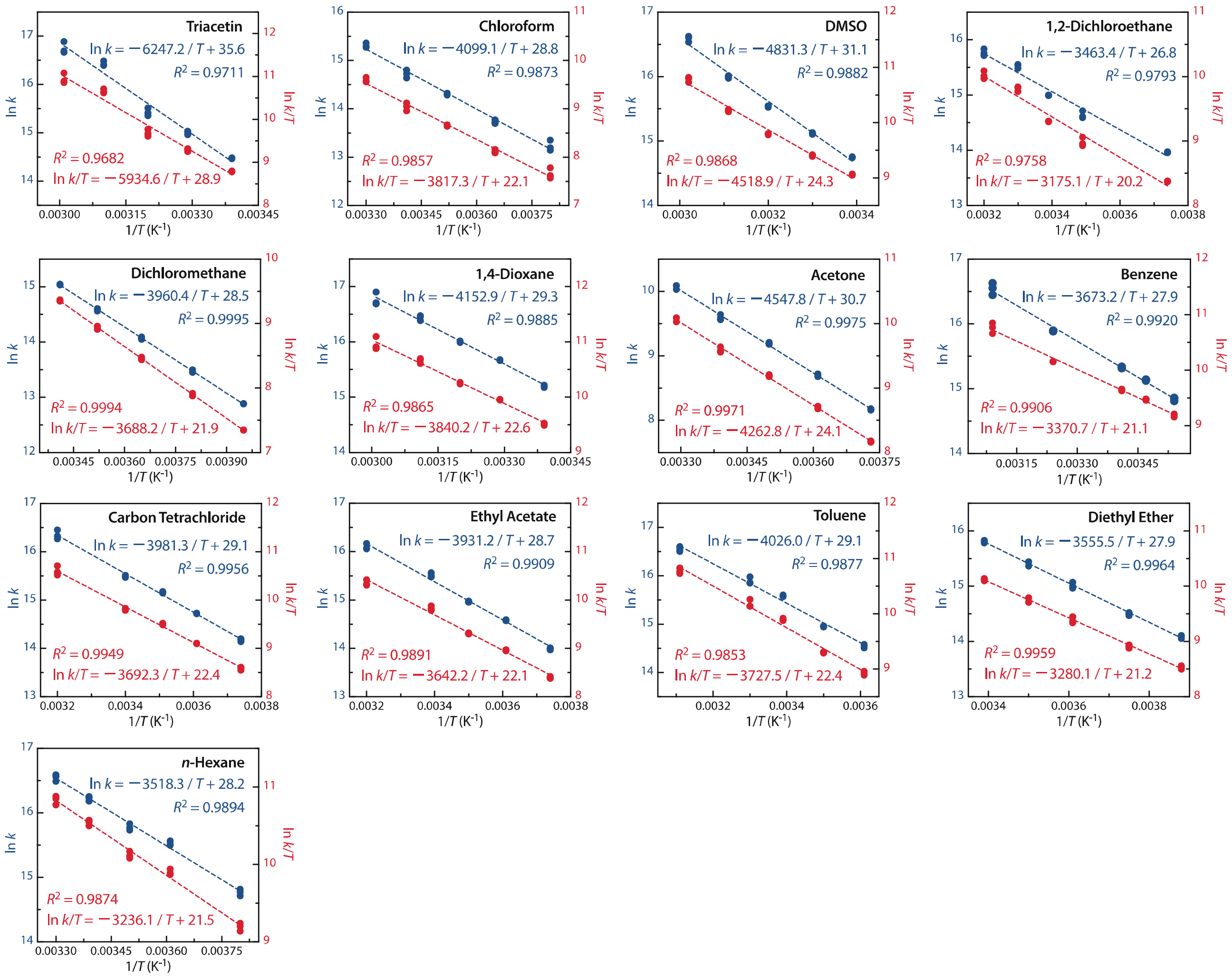

Figure S13. Arrhenius plots (left y axis, blue line) and Eyring plots (right y axis, red line) for the decay processes of S-**DR2b** in triacetin, chloroform, DMSO, 1,2-dichloroethane, dichloromethane, 1,4-dioxane, acetone, benzene, carbon tetrachloride, ethyl acetate, toluene, diethyl ether and *n*-hexane.

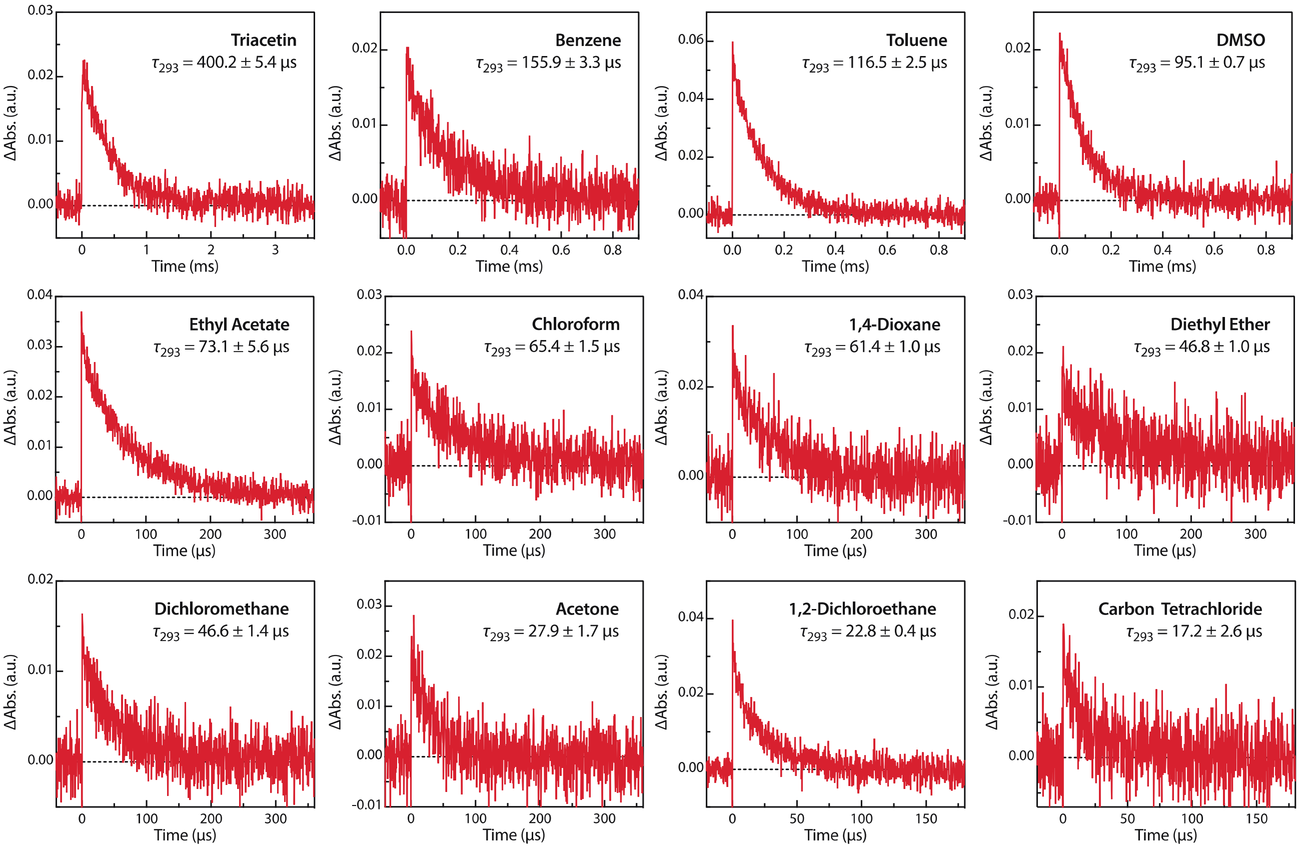

Figure S14. Decay profile monitored at 580 nm of S-**DR3b** at 293 K in triacetin, benzene, toluene, DMSO, ethyl acetate, chloroform, 1,4-dioxane, diethyl ether, dichloromethane, acetone, 1,2-dichloroethane and carbon tetrachloride. Lifetime are calculated from single-exponential decay model fitting.

Table S6. Activation parameters (E_a, log A, $\Delta H^{\ddagger}$, $\Delta S^{\ddagger}$, $\Delta G^{\ddagger}{}_{293}$) of ring-closing processes for S-**DR3b** determined by Arrhenius and Eyring plots.

Entry	Solvent	$E_a{}^a$ / kJ mol^{-1}	log A^a / s^{-1}	$\Delta H^{\ddagger b}$ / kJ mol^{-1}	$\Delta S^{\ddagger b}$ / J mol^{-1} K^{-1}	$\Delta G^{\ddagger b}{}_{293}$ / kJ mol^{-1}
1	Triacetin	72.0 ± 0.9	16.2 ± 0.2	69.6 ± 0.9	57.5 ± 0.8	52.7 ± 0.9
2	Benzene	58.4 ± 1.1	14.2 ± 0.2	56.0 ± 1.1	18.1 ± 2.3	50.7 ± 1.1
3	Toluene	59.9 ± 2.0	14.6 ± 0.4	57.4 ± 2.0	26.5 ± 2.7	49.7 ± 2.0
4	EtOAc	54.8 ± 1.7	13.9 ± 0.3	52.4 ± 1.7	13.9 ± 2.9	48.3 ± 1.7
5	1,4-Dioxane	59.0 ± 1.1	14.7 ± 0.2	56.5 ± 1.1	28.4 ± 1.8	48.2 ± 1.1
6	Ether	52.3 ± 2.1	13.7 ± 0.4	49.9 ± 2.1	8.5 ± 3.2	47.5 ± 2.1
7	Acetone	57.2 ± 2.7	14.7 ± 0.5	54.8 ± 2.7	29.0 ± 4.3	46.3 ± 2.7

Determined from ^{a} Arrhenius plots and ^{b} Eyring plots, respectively, with lifetime of S-**DR3b** at five temperatures between 253 and 333 K, errors are standard errors obtained from regression analysis.

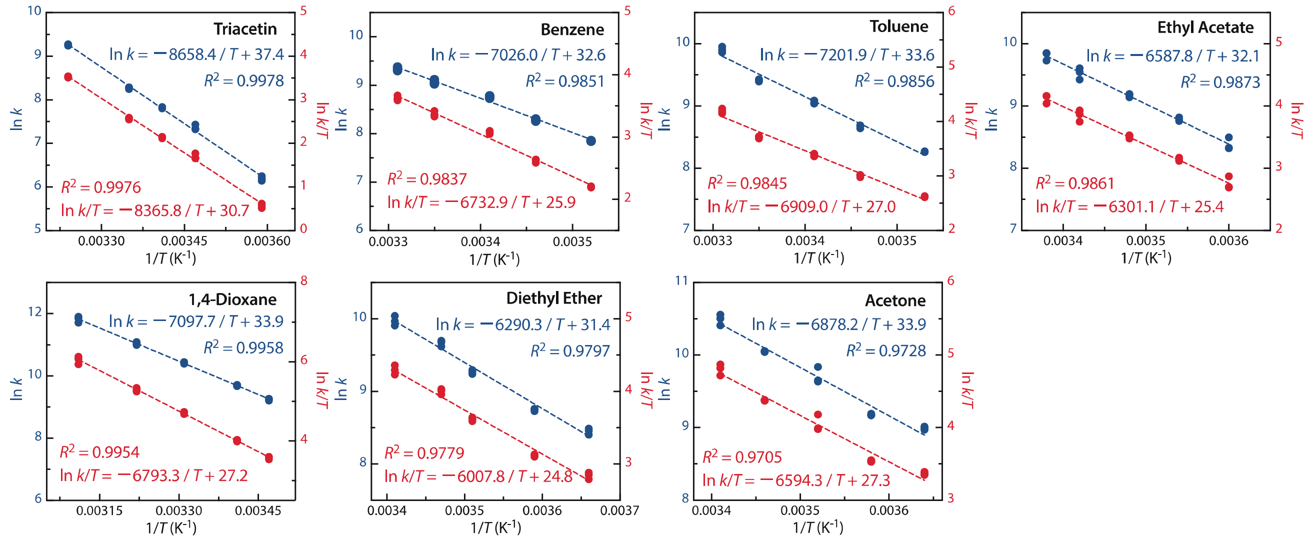

Figure S15. Arrhenius plots (left y axis, blue line) and Eyring plots (right y axis, red line) for the decay processes of S-**DR3b** in triacetin, benzene, toluene, ethyl acetate, 1,4-dioxane, diethyl ether and acetone.

9. Correlation of $E_T(30)$ and Dielectric Constant ε_r on Singlet Diradical

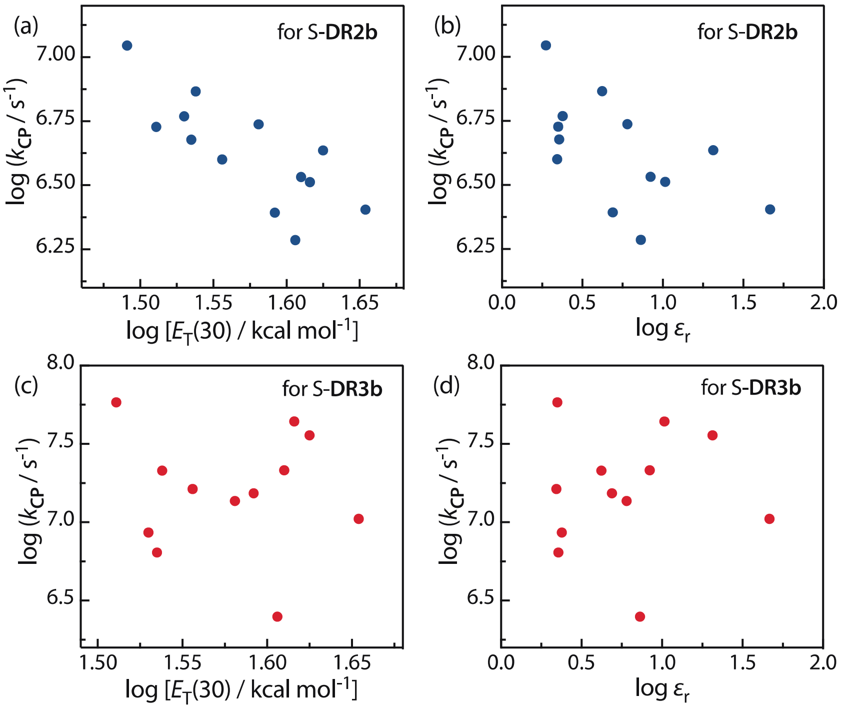

Figure S16. Correlation of rate constant log k_{CP} against $E_T(30)$ and dielectric constant ε_r of solvent for (a,b) S-**DR2b** and (c,d) S-**DR3b**.

10. Computational Details

10.1 General Information

Quantum chemical computations in gas phase have been performed with the *Gaussian 16* (Revision. B.01) suite of programs. Charge, spin multiplicity, number of imaginary frequencies, energies (in Hartree) and Cartesian coordinates (in Å) of computed geometries at (R,U)ωB97X-D/6-31G(d) level of theory are listed in section 10.4. The energy minimum structures and transition state structures were confirmed by vibrational frequency analysis.

Zero-filed splitting (ZFS) parameters (*D* tensor and *E*/*D* ratio) calculation were performed with ORCA 4.2.1 program at B3LYP/EPR-II level of theory. In all ZFS calculations, the resolution of identity and chain of spheres approximation[S1],[S2] (RIJCOSX) and the automatic auxiliary basis sets[S3] (AutoAux) were used. Demo calculations with B3LYP/EPR-III, BP86/EPR-II, BP86/EPR-III and CASSCF(2,2)/def2-SVP[S4] level let to comparable results. The *D* tensor calculation included the spin-spin and the spin-orbit components (SSANDSO).

10.2 Calculated Intrinsic Reaction Coordinate Paths for Transition States (TS)

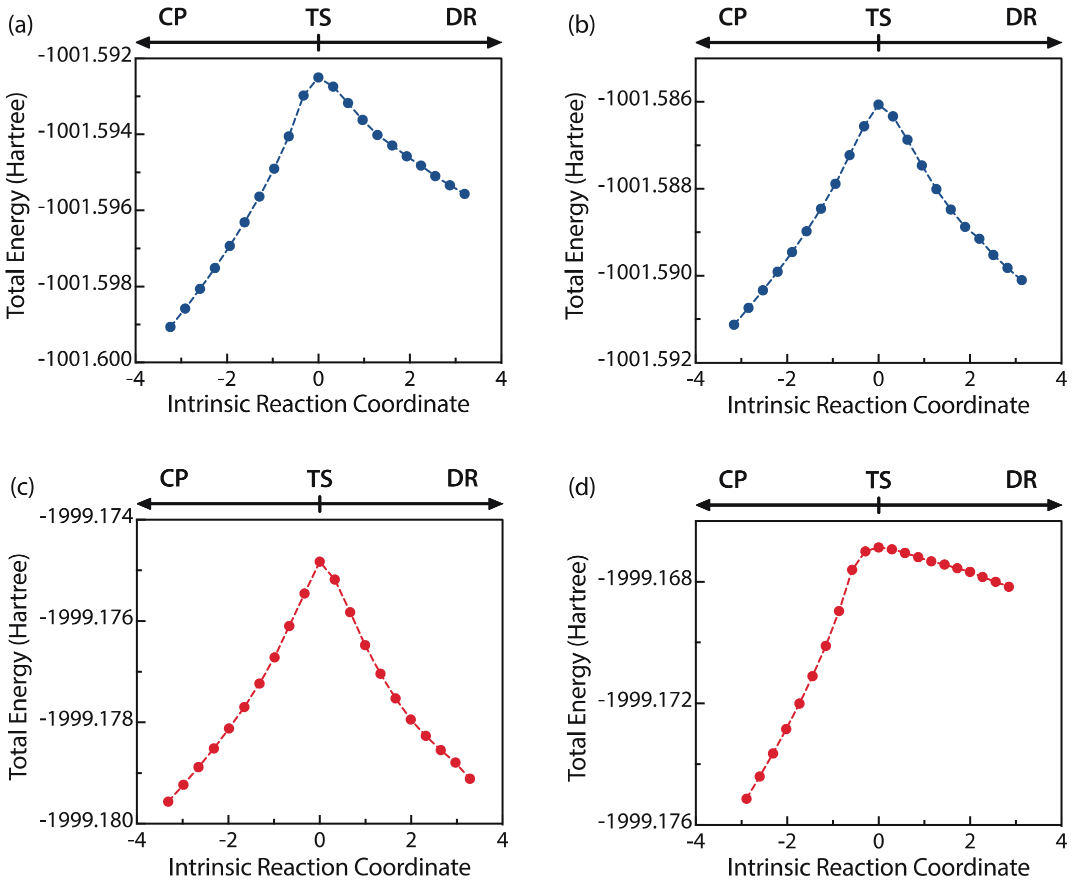

Figure S17. IRC paths for (a) *cis*-**TS2b**, (b) *trans*-**TS2b**, (c) *cis*-**TS3b** and (d) *trans*-**TS3b**.

10.3 Calculated UV-vis Spectra for Macrocyclic Skeleton

TD-DFT calculation of macrocyclic skeleton in **3a** and **3b** were performed at (U)ωB97X-D/6-31G(d) level of theory. 25 excited states were solved and integral equation formalism polarizable continuum model (IEFPCM, implicit solvation model) were considered for benzene environment.

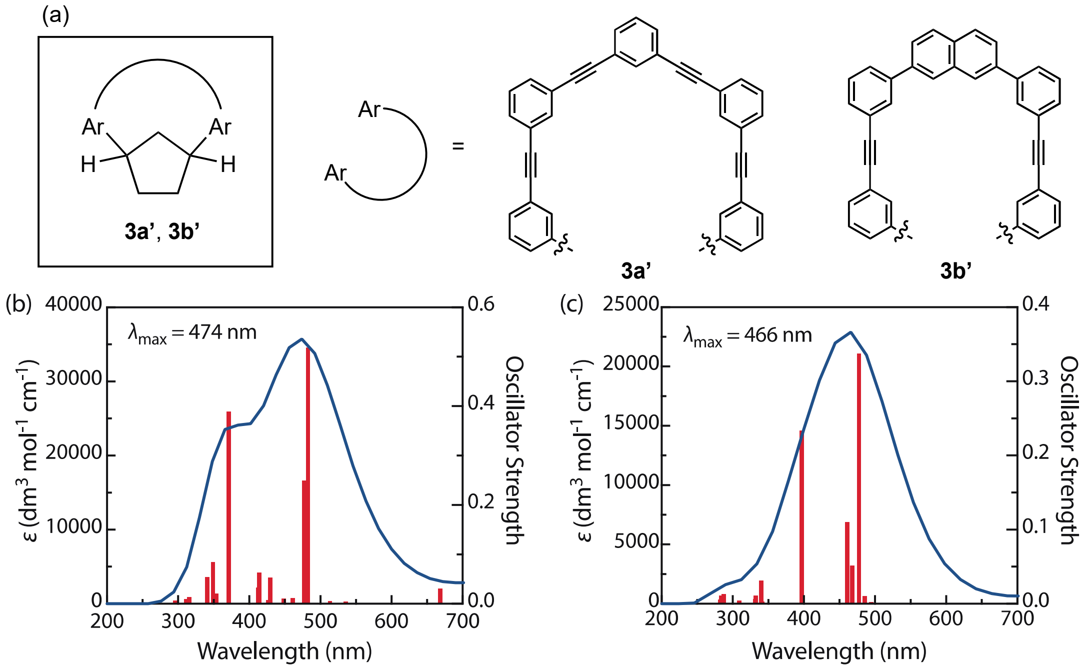

Figure S18. (a) Model molecules for calculations and calculated UV-vis spectra of (b) **3a** and (c) **3b**.

10.4 Cartesian Coordinate of Computed Geometries

Table S7. List and page number of computed geometries.

	**2a**	**3a**	**3b**
**AZ**	20	29	47
S-**DR**	21	31	49
T-**DR**	22	33	51
cis-**TS**	23	35	53
cis-*par*-**CP**	24	37	55
cis-*twi*-**CP**	25	39	57
trans-**TS**	26	41	59
trans-*par*-**CP**	27	43	61
trans-*twi*-**CP**	28	45	63
Macrocyclic Skeleton		**3a**	**3b**
Optimized Skeleton		65	79
**AZ**		67	81
S-**DR**		69	83
cis-*par*-**CP**		71	85
cis-*twi*-**CP**		73	87
trans-*par*-**CP**		75	89
trans-*twi*-**CP**		77	91
Benzene		92	

AZ2b

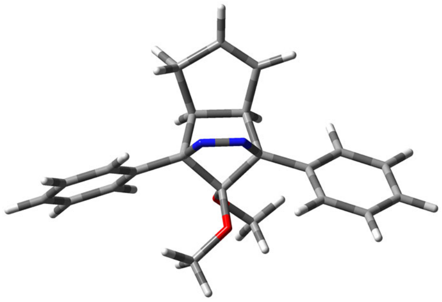

`#p opt freq rwb97xd/6-31g(d)`

Charge = 0, Multiplicity = 1

Number of imaginary frequencies = 0

Sum of electronic and zero-point Energies = -1110.705543 Hartree

Sum of electronic and thermal Energies = -1110.684347 Hartree

Sum of electronic and enthalpy Energies = -1110.683403 Hartree

Sum of electronic and thermal Free Energies = -1110.754757 Hartree

	Cartesian Coordinates				Cartesian Coordinates		
**Atom**	***X***	***Y***	***Z***	**Atom**	***X***	***Y***	***Z***
C	-0.770568	1.591565	0.737055	C	-4.857790	-0.029367	0.567916
H	-1.173418	1.416496	1.739889	H	-3.275792	1.141505	1.409214
C	-1.135280	0.375327	-0.166348	H	-2.206265	-1.357769	-1.908392
C	-0.025014	-0.639803	0.264473	H	-4.546105	-2.146775	-2.062496
C	1.111855	0.347086	-0.164142	H	-5.598379	0.357696	1.261694
C	0.782847	1.575134	0.736341	H	-6.252857	-1.298597	-0.469488
H	1.178231	1.379480	1.733176	C	2.553519	-0.081970	-0.148751
N	0.624100	0.718143	-1.513035	C	5.240907	-0.869684	-0.090623
N	-0.618809	0.737078	-1.520674	C	3.336547	-0.010881	-1.302502
O	0.085005	-0.904045	1.626406	C	3.133175	-0.543585	1.037730
O	-0.169453	-1.796100	-0.484712	C	4.465470	-0.938895	1.064480
C	0.879756	-2.749531	-0.411059	C	4.672478	-0.402918	-1.271254
H	1.707417	-2.474914	-1.072192	H	2.889580	0.347730	-2.223014
H	0.449290	-3.697846	-0.738670	H	2.528113	-0.610682	1.936558
H	1.255992	-2.850629	0.611871	H	4.900180	-1.301533	1.991548
C	-0.992676	-1.628433	2.203068	H	5.269810	-0.342443	-2.176426
H	-0.615809	-2.044725	3.138949	H	6.282496	-1.176428	-0.068595
H	-1.323992	-2.435645	1.543585	C	1.247155	2.948664	0.197590
H	-1.842389	-0.969808	2.412119	H	1.855847	3.483468	0.936105
C	-2.569268	-0.069837	-0.231038	H	1.862463	2.854265	-0.705231
C	-5.224778	-0.954945	-0.403015	C	-0.045123	3.672967	-0.086007
C	-3.539894	0.411825	0.649650	H	-0.065279	4.678760	-0.494367
C	-2.951381	-0.990576	-1.211852	C	-1.126379	2.950826	0.199005
C	-4.266871	-1.430956	-1.294907	H	-2.152707	3.270941	0.051705

S-**DR2b**

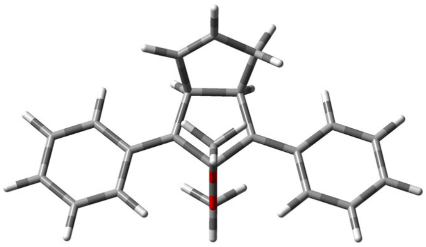

```
#p opt freq uwb97xd/6-31g(d) guess=(mix,always)
```

Charge = 0, Multiplicity = 1, <S^2> = 1.0102

Number of Imaginary Frequencies = 0

Sum of electronic and zero-point Energies = -1001.224799 Hartree

Sum of electronic and thermal Energies = -1001.204066 Hartree

Sum of electronic and enthalpy Energies = -1001.203122 Hartree

Sum of electronic and thermal Free Energies = -1001.274029 Hartree

	Cartesian Coordinates				Cartesian Coordinates		
**Atom**	***X***	***Y***	***Z***	**Atom**	***X***	***Y***	***Z***
C	-1.205066	0.271131	-0.248612	H	-2.126415	-2.073988	0.734161
C	-0.020877	-0.598882	0.116405	H	-4.479162	-2.787293	0.874353
C	1.174694	0.259460	-0.242830	H	-5.727655	0.926168	-0.889058
C	0.778922	1.606303	-0.767120	H	-6.296525	-1.296039	0.068894
C	-0.776048	1.632167	-0.714615	O	-0.026071	-1.034328	1.465260
C	2.526040	-0.190822	-0.149048	O	-0.025838	-1.852510	-0.543158
C	5.206960	-1.063622	0.019666	C	-0.006425	-0.003520	2.424511
C	3.593093	0.604289	-0.636806	H	-0.012752	-0.496342	3.398241
C	2.854204	-1.445491	0.425972	H	-0.884802	0.650108	2.342270
C	4.171416	-1.864841	0.504890	H	0.898923	0.614196	2.340261
C	4.907030	0.172652	-0.552629	C	-0.017672	-1.786704	-1.949147
H	3.380967	1.566184	-1.093536	H	-0.027684	-2.819767	-2.300837
H	2.057830	-2.072257	0.807592	H	0.885199	-1.288840	-2.329596
H	4.397560	-2.828960	0.951270	H	-0.904326	-1.267768	-2.339332
H	5.704598	0.801839	-0.937283	H	-1.210358	1.863892	-1.698939
H	6.237260	-1.400034	0.085937	H	1.128340	1.728157	-1.801055
C	-2.563218	-0.154187	-0.160440	C	-1.085365	2.739369	0.274243
C	-5.260244	-0.978270	0.004308	H	-2.100064	2.960895	0.589858
C	-3.618343	0.677074	-0.613588	C	0.009638	3.352854	0.712357
C	-2.912446	-1.419759	0.378267	H	0.019248	4.162065	1.436740
C	-4.237076	-1.814664	0.455638	C	1.270442	2.809774	0.097631
C	-4.939895	0.269254	-0.531473	H	1.772195	3.568685	-0.516583
H	-3.391640	1.648319	-1.042708	H	1.995954	2.494103	0.857045

T-DR2b

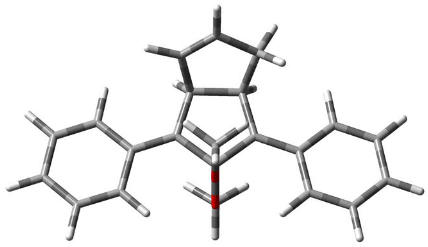

```
#p opt freq uwb97xd/6-31g(d)
```

Charge = 0, Multiplicity = 3, <S^2> = 2.0805

Number of Imaginary Frequencies = 0

Sum of electronic and zero-point Energies = -1001.221904 Hartree

Sum of electronic and thermal Energies = -1001.201140 Hartree

Sum of electronic and enthalpy Energies = -1001.200195 Hartree

Sum of electronic and thermal Free Energies = -1001.272190 Hartree

	Cartesian Coordinates				Cartesian Coordinates		
**Atom**	***X***	***Y***	***Z***	**Atom**	***X***	***Y***	***Z***
C	-1.215694	0.271975	-0.250607	H	-2.126002	-2.085547	0.712154
C	-0.022046	-0.594582	0.110927	H	-4.476752	-2.801834	0.856682
C	1.183664	0.261526	-0.239637	H	-5.738863	0.930227	-0.858025
C	0.781468	1.604779	-0.773271	H	-6.300334	-1.303565	0.078061
C	-0.777972	1.633239	-0.712230	O	-0.030511	-1.032473	1.455493
C	2.528362	-0.192101	-0.142983	O	-0.024071	-1.838295	-0.561657
C	5.207532	-1.075174	0.031293	C	-0.013319	-0.005335	2.420223
C	3.601842	0.601882	-0.623929	H	-0.016877	-0.503232	3.391223
C	2.851805	-1.451976	0.427765	H	-0.894820	0.643999	2.341209
C	4.167246	-1.875131	0.509385	H	0.890073	0.614756	2.336618
C	4.913148	0.164872	-0.537180	C	-0.013009	-1.759469	-1.968043
H	3.394415	1.566293	-1.077557	H	-0.023122	-2.789249	-2.328779
H	2.052506	-2.078787	0.803010	H	0.891642	-1.259450	-2.340547
H	4.388849	-2.842066	0.952035	H	-0.899074	-1.236633	-2.353545
H	5.714287	0.792754	-0.916639	H	-1.219019	1.875617	-1.690625
H	6.236484	-1.415125	0.099787	H	1.130080	1.718319	-1.808510
C	-2.567871	-0.154285	-0.158761	C	-1.077309	2.734838	0.286703
C	-5.264782	-0.983989	0.011426	H	-2.088958	2.953013	0.614247
C	-3.628519	0.681202	-0.597054	C	0.021066	3.351447	0.711228
C	-2.914176	-1.427689	0.367976	H	0.036229	4.163337	1.432405
C	-4.237821	-1.824219	0.447763	C	1.276532	2.815073	0.080377
C	-4.948201	0.270402	-0.512241	H	1.764532	3.576057	-0.542669
H	-3.404968	1.657684	-1.015984	H	2.014679	2.505867	0.829990

cis-**TS2b**

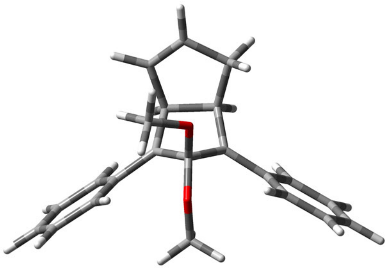

`#p opt=(ts,calcfc) freq uwb97xd/6-31g(d) guess=(mix,always)`

Charge = 0, Multiplicity = 1

Number of Imaginary Frequencies = 1, v_i = -421.21

Sum of electronic and zero-point Energies = -1001.204566 Hartree

Sum of electronic and thermal Energies = -1001.184501 Hartree

Sum of electronic and enthalpy Energies = -1001.183557 Hartree

Sum of electronic and thermal Free Energies = -1001.252744 Hartree

	Cartesian Coordinates				Cartesian Coordinates		
**Atom**	***X***	***Y***	***Z***	**Atom**	***X***	***Y***	***Z***
C	-0.991815	0.388461	-0.233452	H	-2.362245	-0.885643	1.738215
C	0.040784	0.300546	0.849923	H	-4.430585	-2.201091	1.510759
C	1.071250	0.411710	-0.211868	H	-4.380485	-1.549555	-2.732796
C	0.815463	1.604545	-1.096813	H	-5.455372	-2.547964	-0.726797
C	-0.721736	1.572584	-1.140957	O	0.128696	1.318487	1.809215
C	2.294087	-0.378193	-0.275372	O	0.029106	-0.903286	1.592819
C	4.713857	-1.806781	-0.374739	C	-1.043959	1.527571	2.563380
C	2.926962	-0.644364	-1.501398	H	-0.883354	2.439271	3.142517
C	2.906619	-0.844104	0.903277	H	-1.237912	0.692229	3.246990
C	4.102931	-1.546457	0.850539	H	-1.916337	1.672984	1.910858
C	4.120242	-1.352732	-1.550467	C	0.069728	-2.159710	0.938518
H	2.468412	-0.307686	-2.426483	H	-0.798940	-2.747988	1.250329
H	2.432120	-0.635757	1.856337	H	0.987352	-2.681486	1.228289
H	4.565068	-1.888649	1.771988	H	0.052567	-2.065941	-0.150721
H	4.588148	-1.554340	-2.509668	H	-1.098609	1.396080	-2.153795
H	5.648331	-2.358704	-0.413140	H	1.240482	1.460773	-2.093014
C	-2.181872	-0.444687	-0.363976	C	-1.148153	2.922192	-0.614799
C	-4.545317	-1.964196	-0.626250	H	-2.189510	3.210378	-0.509309
C	-2.781774	-0.652985	-1.620338	C	-0.099059	3.671150	-0.278251
C	-2.803820	-1.027069	0.758548	H	-0.163627	4.666910	0.152138
C	-3.968032	-1.771339	0.626889	C	1.225914	2.988879	-0.518177
C	-3.943767	-1.401468	-1.749407	H	1.850802	3.553175	-1.220661
H	-2.322924	-0.236565	-2.511346	H	1.793003	2.886647	0.414185

cis-*par*-**CP2b**

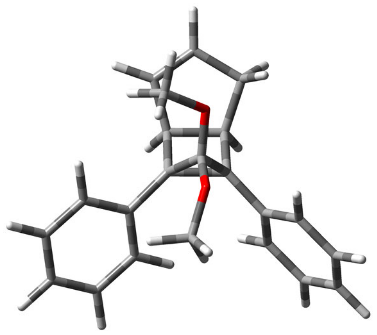

`#p opt freq rwb97xd/6-31g(d)`

Charge = 0, Multiplicity = 1

Number of Imaginary Frequencies = 0

Sum of electronic and zero-point Energies = -1001.223623 Hartree

Sum of electronic and thermal Energies = -1001.203434 Hartree

Sum of electronic and enthalpy Energies = -1001.202489 Hartree

Sum of electronic and thermal Free Energies = -1001.272171 Hartree

	Cartesian Coordinates				Cartesian Coordinates		
**Atom**	***X***	***Y***	***Z***	**Atom**	***X***	***Y***	***Z***
C	-0.806420	0.252994	-0.259625	H	-3.277539	-0.160294	0.669398
C	-0.147892	0.893540	0.944579	H	-4.635291	-2.227665	0.554701
C	0.737844	0.648414	-0.236485	H	-1.374702	-4.247373	-1.375317
C	0.487473	1.645191	-1.386576	H	-3.682012	-4.284697	-0.459921
C	-1.007967	1.259529	-1.427102	O	-0.401317	2.184774	1.340212
C	1.997182	-0.141683	-0.165657	O	0.080903	0.162511	2.113730
C	4.422514	-1.550435	-0.094328	C	-1.654560	2.346045	1.969644
C	2.390181	-0.946736	-1.240196	H	-1.737127	3.402470	2.231077
C	2.848010	-0.039136	0.939271	H	-1.724553	1.732446	2.873963
C	4.049029	-0.741208	0.974862	H	-2.471534	2.086278	1.282063
C	3.589767	-1.649038	-1.206102	C	0.040209	-1.249103	2.183297
H	1.749157	-1.017567	-2.116085	H	-0.986395	-1.627464	2.182808
H	2.552943	0.584934	1.776113	H	0.516578	-1.499350	3.134338
H	4.697390	-0.652661	1.841856	H	0.601418	-1.731934	1.377607
H	3.875449	-2.272195	-2.048625	H	-1.300880	0.695162	-2.319566
H	5.359679	-2.098381	-0.064158	H	1.033934	1.325298	-2.277287
C	-1.561139	-1.028564	-0.289070	C	-1.744867	2.561642	-1.298463
C	-3.091942	-3.374109	-0.416298	H	-2.827497	2.644364	-1.310346
C	-1.041796	-2.185379	-0.872480	C	-0.897442	3.587117	-1.201733
C	-2.865765	-1.057775	0.214679	H	-1.199049	4.627274	-1.111284
C	-3.625956	-2.220186	0.153384	C	0.559511	3.175294	-1.187012
C	-1.798606	-3.352198	-0.929419	H	1.123998	3.665125	-1.989879
H	-0.027887	-2.176900	-1.259392	H	1.033023	3.449539	-0.238626

cis-*twi*-**CP2b**

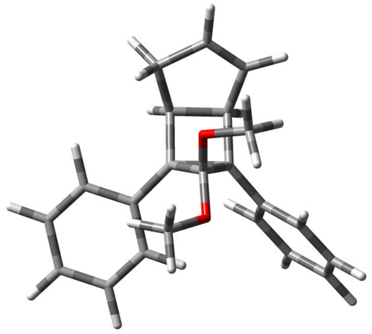

`#p opt freq rwb97xd/6-31g(d)`

Charge = 0, Multiplicity = 1

Number of Imaginary Frequencies = 0

Sum of electronic and zero-point Energies = -1001.239352 Hartree

Sum of electronic and thermal Energies = -1001.218972 Hartree

Sum of electronic and enthalpy Energies = -1001.218028 Hartree

Sum of electronic and thermal Free Energies = -1001.288039 Hartree

Atom	Cartesian Coordinates			Atom	Cartesian Coordinates		
	X	*Y*	*Z*		*X*	*Y*	*Z*
H	1.271657	0.727462	-2.410723	H	-3.162880	1.258121	-0.973702
C	1.008558	1.276048	-1.499960	H	-0.847815	-1.982167	0.623137
C	-0.490691	1.646580	-1.396264	H	-2.875146	-3.382447	0.714966
H	-1.063185	1.330533	-2.271783	H	-5.194932	-0.137878	-0.873185
C	-0.670908	0.623687	-0.259605	H	-5.063687	-2.471042	-0.026413
C	0.871237	0.264630	-0.331534	C	1.539986	-1.061028	-0.356217
C	0.264919	0.870223	0.894807	C	2.850964	-3.532900	-0.496663
O	0.498929	2.158474	1.330355	C	1.232992	-1.974221	-1.369473
O	0.181342	-0.043228	1.934838	C	2.509046	-1.405066	0.589147
C	1.789057	2.327029	1.884611	C	3.160742	-2.632059	0.519014
H	1.903960	1.747354	2.808599	C	1.882684	-3.201742	-1.441040
H	1.899898	3.391055	2.100431	H	0.463906	-1.721846	-2.095401
H	2.563167	2.022602	1.168269	H	2.739363	-0.709016	1.389678
C	-0.816344	0.277784	2.892108	H	3.911595	-2.886758	1.261497
H	-0.872112	-0.576246	3.568996	H	1.628955	-3.902360	-2.231278
H	-1.791754	0.427858	2.416178	H	3.359167	-4.491316	-0.549488
H	-0.548206	1.178973	3.454222	C	-0.567907	3.172952	-1.172904
C	-1.860571	-0.261280	-0.180772	H	-1.010175	3.431296	-0.204759
C	-4.171262	-1.853615	-0.069279	H	-1.164014	3.669521	-1.948727
C	-3.097901	0.238383	-0.602212	C	0.883734	3.599924	-1.232633
C	-1.797247	-1.577577	0.292438	H	1.178322	4.641366	-1.134761
C	-2.944135	-2.362584	0.347237	C	1.736187	2.584754	-1.379340
C	-4.243981	-0.547500	-0.544644	H	2.816807	2.678432	-1.428225

trans-**TS2b**

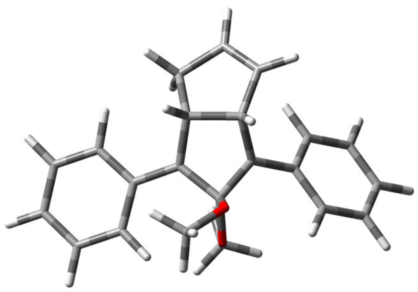

`#p opt=(ts,calcfc) freq uwb97xd/6-31g(d) guess=(mix,always)`

Charge = 0, Multiplicity = 1

Number of Imaginary Frequencies = 1, ν_i = -449.91

Sum of electronic and zero-point Energies = -1001.197863 Hartree

Sum of electronic and thermal Energies = -1001.177815 Hartree

Sum of electronic and enthalpy Energies = -1001.176871 Hartree

Sum of electronic and thermal Free Energies = -1001.246214 Hartree

	**Cartesian Coordinates**				**Cartesian Coordinates**		
**Atom**	***X***	***Y***	***Z***	**Atom**	***X***	***Y***	***Z***
C	0.830281	1.527836	1.280027	H	5.650468	-1.215774	-1.800275
H	0.992843	1.361277	2.353459	C	-2.246528	0.011722	-0.287317
C	1.526668	2.765085	0.790768	C	-4.770877	-0.557400	-1.455257
H	2.564759	2.982614	1.019215	C	-3.078790	1.032296	-0.792841
C	0.718246	3.519111	0.052882	C	-2.750911	-1.306221	-0.362721
H	0.990546	4.456146	-0.424140	C	-3.982268	-1.582243	-0.939045
C	-0.636288	2.885615	-0.122385	C	-4.312762	0.753002	-1.365832
H	-1.453081	3.597002	0.046554	H	-2.774467	2.067204	-0.726762
H	-0.738157	2.517235	-1.151389	H	-2.173425	-2.117189	0.060259
C	-0.659786	1.711436	0.909561	H	-4.333293	-2.609718	-0.973676
H	-1.300901	1.992680	1.758125	H	-5.734038	-0.775489	-1.906715
C	1.061951	0.231570	0.545552	H	4.533584	0.896723	-2.471139
C	-0.983954	0.325326	0.388984	H	-4.921352	1.570280	-1.742172
C	-0.020953	-0.695077	0.935232	O	-0.041123	-0.968960	2.310824
C	2.300906	-0.161081	-0.125698	O	-0.015067	-1.954864	0.296600
C	4.715872	-0.921075	-1.332042	C	-1.255937	-1.504204	2.793091
C	2.937792	-1.359205	0.238677	H	-2.109963	-0.869891	2.519245
C	2.894649	0.644744	-1.107911	H	-1.170762	-1.537475	3.881316
C	4.090574	0.265924	-1.705969	H	-1.428569	-2.517815	2.412999
C	4.136377	-1.730827	-0.357067	C	0.129894	-2.039546	-1.112289
H	2.478367	-1.989254	0.993473	H	0.006018	-1.069775	-1.602319
H	2.401858	1.563895	-1.406760	H	-0.636726	-2.721798	-1.492117
H	4.620904	-2.656178	-0.059427	H	1.119961	-2.434815	-1.357551

*trans-par-***CP2b**

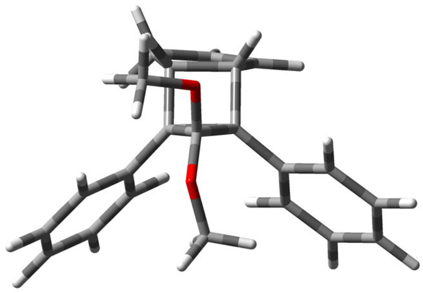

```
#p opt freq rwb97xd/6-31g(d)
```

Charge = 0, Multiplicity = 1

Number of Imaginary Frequencies = 0

Sum of electronic and zero-point Energies = -1001.234714 Hartree

Sum of electronic and thermal Energies = -1001.214249 Hartree

Sum of electronic and enthalpy Energies = -1001.213305 Hartree

Sum of electronic and thermal Free Energies = -1001.284551 Hartree

	Cartesian Coordinates				Cartesian Coordinates		
**Atom**	***X***	***Y***	***Z***	**Atom**	***X***	***Y***	***Z***
C	-0.736578	1.649513	1.365185	H	4.401201	-1.399011	-1.929181
C	0.823234	1.612404	1.371008	H	2.621916	-3.498163	1.366727
C	0.787480	0.620703	0.188129	H	4.340165	-3.370686	-0.421891
C	-0.784502	0.718546	0.138624	C	-1.918879	-0.220982	-0.063329
C	0.054268	1.302212	-0.953501	C	-4.153379	-1.874689	-0.407869
O	0.074581	2.675155	-1.081698	C	-1.930238	-1.514165	0.459346
O	0.130714	0.751539	-2.229422	C	-3.047646	0.240540	-0.749085
C	1.275219	3.171273	-1.641527	C	-4.156854	-0.579279	-0.921684
H	2.149935	2.799461	-1.088270	C	-3.038248	-2.338554	0.283146
H	1.232145	4.258924	-1.557230	H	-1.061162	-1.879671	0.994500
H	1.370396	2.886620	-2.694257	H	-3.039154	1.246973	-1.159487
C	-0.233144	-0.587092	-2.512840	H	-5.023854	-0.209465	-1.461454
H	0.282900	-0.838954	-3.442507	H	-3.028700	-3.346818	0.687067
H	-1.313137	-0.682689	-2.659619	H	-5.016886	-2.518707	-0.545928
H	0.086185	-1.290867	-1.739223	C	1.254729	1.068064	2.744343
C	1.746843	-0.499138	0.005747	H	1.995384	0.264035	2.661527
C	3.618192	-2.568528	-0.300591	H	1.723133	1.854901	3.350557
C	1.727587	-1.613621	0.851470	C	-0.040653	0.609432	3.367870
C	2.725702	-0.435878	-0.991443	H	-0.079192	0.090559	4.321439
C	3.651773	-1.462386	-1.145468	C	-1.105700	0.940415	2.639324
C	2.653903	-2.640559	0.700993	H	-2.135352	0.715059	2.898679
H	0.981981	-1.665884	1.640456	H	1.327085	2.550157	1.127083
H	2.740635	0.416626	-1.663346	H	-1.236630	2.614667	1.236227

trans-*twi*-**CP2b**

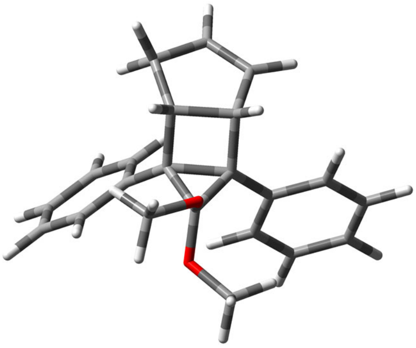

```
#p opt freq rwb97xd/6-31g(d)
```

Charge = 0, Multiplicity = 1

Number of Imaginary Frequencies = 0

Sum of electronic and zero-point Energies = -1001.248724 Hartree

Sum of electronic and thermal Energies = -1001.228311 Hartree

Sum of electronic and enthalpy Energies = -1001.227367 Hartree

Sum of electronic and thermal Free Energies = -1001.297431 Hartree

	Cartesian Coordinates				Cartesian Coordinates		
**Atom**	***X***	***Y***	***Z***	**Atom**	***X***	***Y***	***Z***
C	0.642958	-1.660103	1.430720	H	-4.315471	1.711708	-1.837443
C	-0.918990	-1.609793	1.365211	H	-2.185249	3.669816	1.336151
C	-0.827337	-0.618630	0.183396	H	-3.994803	3.683027	-0.364623
C	0.734315	-0.737018	0.205636	C	1.798448	0.277002	0.001652
C	-0.088451	-1.302732	-0.924721	C	3.882421	2.120379	-0.381425
O	-0.125307	-2.677655	-1.090299	C	1.562495	1.495972	-0.644135
O	-0.088887	-0.595472	-2.111229	C	3.095446	-0.002557	0.446943
C	-1.332499	-3.121222	-1.682104	C	4.128719	0.908860	0.258296
H	-2.202115	-2.792624	-1.095862	C	2.596964	2.406727	-0.832392
H	-1.294188	-4.211909	-1.694244	H	0.567720	1.724820	-1.008559
H	-1.436833	-2.745404	-2.706774	H	3.298264	-0.950905	0.937841
C	1.005261	-0.910236	-2.960543	H	5.128096	0.670610	0.611151
H	0.946291	-0.216581	-3.800660	H	2.393900	3.348491	-1.334318
H	0.935934	-1.940876	-3.325770	H	4.686598	2.835418	-0.527986
H	1.961294	-0.775064	-2.443097	C	-1.413104	-1.048229	2.709489
C	-1.689088	0.575947	0.003506	H	-2.116724	-0.217670	2.578007
C	-3.350732	2.814690	-0.260319	H	-1.943180	-1.815236	3.289284
C	-1.511519	1.694765	0.823809	C	-0.144706	-0.624549	3.405647
C	-2.705239	0.596178	-0.954574	H	-0.147723	-0.111998	4.363211
C	-3.531317	1.707654	-1.085788	C	0.951772	-0.964771	2.730918
C	-2.337145	2.806783	0.694434	H	1.966335	-0.756394	3.055133
H	-0.707721	1.686853	1.555934	H	-1.416452	-2.546674	1.104771
H	-2.834209	-0.260462	-1.609403	H	1.131750	-2.634626	1.327546

AZ3a

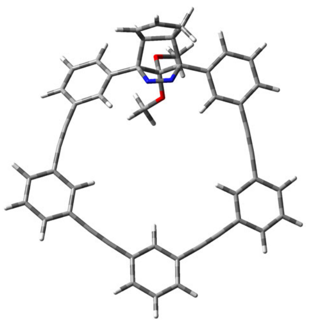

```
#p opt freq rwb97xd/6-31g(d)
```

Charge = 0, Multiplicity = 1

Number of Imaginary Frequencies = 0

Sum of electronic and zero-point Energies = -2106.671553 Hartree

Sum of electronic and thermal Energies = -2106.629861 Hartree

Sum of electronic and enthalpy Energies = -2106.628917 Hartree

Sum of electronic and thermal Free Energies = -2106.747817 Hartree

	Cartesian Coordinates				Cartesian Coordinates		
**Atom**	***X***	***Y***	***Z***	**Atom**	***X***	***Y***	***Z***
O	-2.506316	0.190311	-1.279546	C	4.654383	3.827246	-0.095585
O	-4.710842	-0.401934	-1.610764	C	-2.651242	2.762353	0.208830
N	-2.921942	0.210285	1.475550	H	-1.770469	2.131652	0.191042
N	-2.715643	-1.000334	1.289294	C	-5.300272	0.056596	1.060786
C	3.317480	-4.092672	0.091356	H	-6.127115	0.383445	0.427168
H	2.852897	-3.118582	-0.014913	C	4.507285	-6.589943	0.360867
C	3.663719	4.849210	-0.241798	H	4.971733	-7.565302	0.467721
C	-2.508724	4.146349	0.063626	C	-5.030692	3.010943	0.423503
C	-3.907489	2.183358	0.382721	H	-6.017670	2.586296	0.581448
C	4.698479	-4.181601	0.292456	C	2.310809	4.504383	-0.158723
C	-3.684594	-0.230747	-0.688274	H	2.029228	3.472111	0.018026
C	6.621874	-0.602021	0.351907	C	-5.604314	0.271333	2.562398
C	6.031665	-1.905138	0.355509	H	-4.907636	0.979113	3.026988
C	-3.610469	-1.499894	0.224937	H	-6.612430	0.675562	2.715630
C	-1.393430	-4.452068	-0.677699	C	-3.948394	-3.502574	-1.274127
C	5.792143	0.514725	0.206071	H	-4.928315	-3.119984	-1.540469
H	4.721151	0.377477	0.105214	C	-5.039886	0.744195	-2.383102
C	-1.879827	-3.289869	-0.066256	H	-5.670878	1.434939	-1.813087
H	-1.251738	-2.750507	0.633520	H	-5.591367	0.380833	-3.251849
C	-3.986181	0.691371	0.539626	H	-4.140140	1.274269	-2.708796
C	4.019620	6.186320	-0.466382	C	1.687009	6.811117	-0.521178
H	5.068786	6.455228	-0.530907	H	0.916791	7.567883	-0.627521
C	2.528686	-5.245113	0.018164	C	8.538962	0.865831	0.465368
C	-0.044126	-4.854181	-0.418028	H	9.610955	1.003152	0.567197

	Cartesian Coordinates				Cartesian Coordinates		
Atom	*X*	*Y*	*Z*	Atom	*X*	*Y*	*Z*
C	-1.195716	4.693566	-0.089291	C	-3.647956	4.962031	0.089670
C	6.328868	1.805949	0.187308	H	-3.541112	6.036154	-0.021580
C	-1.976443	-0.608973	-2.329429	C	3.031559	7.154177	-0.604465
H	-1.303514	-1.374717	-1.933706	H	3.312691	8.188217	-0.778261
H	-1.417302	0.068989	-2.977590	C	-3.481366	-4.673771	-1.863477
H	-2.773150	-1.091938	-2.902705	H	-4.111725	-5.210697	-2.565856
C	1.315997	5.478291	-0.296142	C	-4.899437	4.389410	0.271014
C	5.448590	2.923483	0.035227	H	-5.781844	5.021116	0.304436
C	5.463126	-2.973433	0.340505	C	-2.209271	-5.152110	-1.574958
C	-0.056635	5.088526	-0.195778	H	-1.835189	-6.053635	-2.049079
C	3.134870	-6.500715	0.154865	C	-5.038063	-1.465691	0.866977
H	2.525717	-7.396879	0.099522	H	-5.742169	-1.922846	0.167033
C	1.122934	-5.095225	-0.207062	C	-5.157441	-2.039214	2.251411
C	-3.149215	-2.801624	-0.366896	H	-4.996632	-3.091552	2.460678
C	7.714210	1.975140	0.318167	C	8.004945	-0.417587	0.482933
H	8.132749	2.976053	0.304373	H	8.650179	-1.282464	0.596425
C	5.291549	-5.443973	0.429183	C	-5.466659	-1.110211	3.153020
H	6.362664	-5.516855	0.586202	H	-5.600315	-1.298528	4.213744

S-**DR3a**

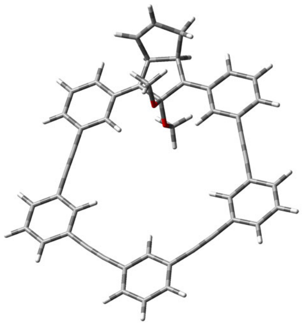

`#p opt freq uwb97xd/6-31g(d) guess=(mix,always)`

Charge = 0, Multiplicity = 1, <S^2> = 1.0228

Number of Imaginary Frequencies = 0

Sum of electronic and zero-point Energies = -1997.192486 Hartree

Sum of electronic and thermal Energies = -1997.151254 Hartree

Sum of electronic and enthalpy Energies = -1997.150310 Hartree

Sum of electronic and thermal Free Energies = -1997.268773 Hartree

	**Cartesian Coordinates**				**Cartesian Coordinates**		
**Atom**	***X***	***Y***	***Z***	**Atom**	***X***	***Y***	***Z***
H	-6.286287	1.048795	-1.059067	C	2.737218	4.191644	-0.022341
C	-5.777497	0.717346	-0.144170	C	4.099707	4.501932	0.050308
C	-6.573449	1.207874	1.106477	C	3.546793	6.853923	0.016238
C	-5.755692	-0.835924	-0.071370	H	1.449650	7.334331	-0.098817
H	-7.515022	1.693534	0.819534	H	2.422590	3.153910	-0.037819
H	-6.005878	1.946118	1.685764	H	3.861544	7.892775	0.031365
H	-6.332543	-1.286246	-0.893193	H	5.557405	6.084780	0.126113
C	-6.826115	-0.050176	1.890790	C	1.880169	-5.191181	-0.101679
H	-7.304061	-0.039428	2.865900	C	4.618651	-5.771706	-0.005267
C	-6.404949	-1.144223	1.264095	C	2.818543	-4.156648	-0.042266
H	-6.475935	-2.156511	1.649308	C	2.325537	-6.520954	-0.112609
C	-4.312492	-1.241768	-0.146679	C	3.685985	-6.800766	-0.064416
C	-4.339558	1.137394	-0.171183	C	4.188284	-4.438268	0.005963
C	-3.386548	-0.041991	-0.147088	H	2.481309	-3.125956	-0.033771
C	-3.871247	-2.597129	-0.210600	H	1.598303	-7.324898	-0.158790
C	-3.033541	-5.292715	-0.333661	H	4.023323	-7.832581	-0.073133
C	-2.496046	-2.932408	-0.175143	H	5.680828	-5.989928	0.032029
C	-4.805256	-3.658370	-0.313297	C	5.114195	-3.349970	0.064306
C	-4.387510	-4.978069	-0.374122	C	5.835222	-2.379272	0.110634
C	-2.081759	-4.262643	-0.233826	C	5.048111	3.432759	0.100802
H	-1.757617	-2.144389	-0.095064	C	5.788418	2.476270	0.137762
H	-5.867342	-3.439400	-0.355070	C	6.580939	-1.160008	0.159752
H	-5.123233	-5.772547	-0.455496	C	7.956794	1.277257	0.250910
H	-2.701118	-6.324440	-0.378811	C	5.879858	0.049218	0.128921

Atom	Cartesian Coordinates			Atom	Cartesian Coordinates		
	X	*Y*	*Z*		*X*	*Y*	*Z*
C	-3.919200	2.499157	-0.266845	C	7.979940	-1.140102	0.236860
C	-3.128218	5.204556	-0.455947	C	8.654665	0.074923	0.281806
C	-4.868892	3.537361	-0.435403	C	6.557675	1.271237	0.173751
C	-2.552700	2.861884	-0.200291	H	4.797145	0.039194	0.069035
C	-2.161225	4.197371	-0.291813	H	8.527354	-2.076581	0.260973
C	-4.474103	4.862341	-0.528311	H	9.738539	0.084960	0.341572
H	-5.924593	3.294672	-0.501541	H	8.486188	2.223699	0.285897
H	-1.802618	2.091739	-0.070308	O	-2.479501	-0.019952	0.939265
H	-5.221389	5.639055	-0.660099	O	-2.473383	-0.043596	-1.227381
H	-2.813655	6.240494	-0.525960	C	-3.073408	0.004316	2.216653
C	-0.688703	-4.587619	-0.188970	H	-3.694456	0.899880	2.359792
C	0.484865	-4.880589	-0.149824	H	-2.249672	0.022969	2.931835
C	-0.776374	4.550537	-0.215800	H	-3.692529	-0.884085	2.397846
C	0.389973	4.867103	-0.151948	C	-3.058257	-0.059525	-2.508208
C	1.777367	5.206544	-0.076526	H	-3.670883	-0.957934	-2.666770
C	4.500878	5.844353	0.069738	H	-2.230027	-0.064556	-3.218411
C	2.193619	6.545620	-0.056774	H	-3.676243	0.831587	-2.686254

T-**DR3a**

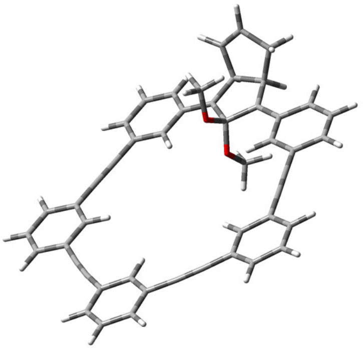

```
#p opt freq uwb97xd/6-31g(d)
```

Charge = 0, Multiplicity = 3, <S^2> = 2.0054

Number of Imaginary Frequencies = 0

Sum of electronic and zero-point Energies = -1997.189636 Hartree

Sum of electronic and thermal Energies = -1997.148366 Hartree

Sum of electronic and enthalpy Energies = -1997.147422 Hartree

Sum of electronic and thermal Free Energies = -1997.266949 Hartree

	Cartesian Coordinates				Cartesian Coordinates		
**Atom**	***X***	***Y***	***Z***	**Atom**	***X***	***Y***	***Z***
H	6.277633	-1.039551	-1.091143	C	-2.739108	-4.190639	-0.016715
C	5.783474	-0.713507	-0.166222	C	-4.100936	-4.503485	0.057591
C	6.601442	-1.210910	1.067689	C	-3.543284	-6.854400	0.031109
C	5.758801	0.843592	-0.078004	H	-1.445224	-7.330882	-0.083382
H	7.547154	-1.678124	0.763440	H	-2.426664	-3.152310	-0.035840
H	6.050987	-1.966049	1.641302	H	-3.855887	-7.893841	0.049854
H	6.336783	1.304960	-0.892445	H	-5.555428	-6.088985	0.139343
C	6.844544	0.038440	1.868410	C	-1.889785	5.186920	-0.103235
H	7.333398	0.018963	2.837892	C	-4.628820	5.766874	-0.016389
C	6.408182	1.137333	1.261319	C	-2.828072	4.152286	-0.044423
H	6.472093	2.144745	1.660311	C	-2.335599	6.516530	-0.118428
C	4.315782	1.256057	-0.145700	C	-3.696259	6.796065	-0.074992
C	4.346007	-1.142298	-0.173035	C	-4.198038	4.433630	-0.000919
C	3.396366	0.045628	-0.145077	H	-2.490624	3.121700	-0.032601
C	3.870557	2.605219	-0.199542	H	-1.608456	7.320580	-0.164169
C	3.022834	5.300294	-0.306014	H	-4.033847	7.827766	-0.087014
C	2.492433	2.936422	-0.169537	H	-5.691169	5.984840	0.017242
C	4.801552	3.672868	-0.288380	C	-5.123780	3.345172	0.057163
C	4.378735	4.990534	-0.341132	C	-5.844619	2.374343	0.103717
C	2.074380	4.265478	-0.219992	C	-5.051440	-3.436030	0.104926
H	1.755923	2.145604	-0.100805	C	-5.793491	-2.480787	0.139014
H	5.864743	3.458136	-0.325197	C	-6.589557	1.154639	0.153650
H	5.111766	5.788579	-0.411771	C	-7.963370	-1.283725	0.246801
H	2.686826	6.331070	-0.344757	C	-5.887379	-0.054011	0.125981

Atom	Cartesian Coordinates			Atom	Cartesian Coordinates		
	X	*Y*	*Z*		*X*	*Y*	*Z*
C	3.923254	-2.498161	-0.269270	C	-7.988651	1.133623	0.228580
C	3.124946	-5.203051	-0.462004	C	-8.662349	-0.081946	0.274534
C	4.870338	-3.540132	-0.444453	C	-6.564147	-1.276560	0.171835
C	2.554757	-2.859383	-0.198740	H	-4.804587	-0.043118	0.067784
C	2.160817	-4.193678	-0.291718	H	-8.536957	2.069639	0.250240
C	4.471709	-4.863152	-0.539292	H	-9.746306	-0.092852	0.332604
H	5.926314	-3.299761	-0.514779	H	-8.491986	-2.230571	0.282559
H	1.806263	-2.088412	-0.065039	O	2.496369	0.022084	0.943036
H	5.216579	-5.641319	-0.676395	O	2.488863	0.046562	-1.227044
H	2.807817	-6.238083	-0.533165	C	3.094077	-0.002586	2.219741
C	0.680057	4.585985	-0.180839	H	3.718657	-0.896098	2.357830
C	-0.494143	4.877112	-0.146272	H	2.271938	-0.026176	2.936418
C	0.775661	-4.545049	-0.211902	H	3.708782	0.888729	2.400027
C	-0.390422	-4.862175	-0.145548	C	3.076562	0.064948	-2.507711
C	-1.777191	-5.203714	-0.068019	H	3.688233	0.964402	-2.662146
C	-4.499417	-5.846612	0.081679	H	2.249499	0.070904	-3.219056
C	-2.190786	-6.543560	-0.043591	H	3.694133	-0.826294	-2.684941

cis-**TS3a**

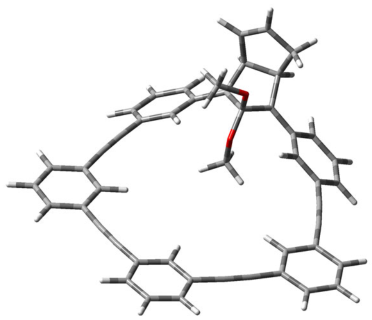

```
#p opt=(ts,calcfc) freq uwb97xd/6-31g(d) guess=(mix,always)
```

Charge = 0, Multiplicity = 1

Number of Imaginary Frequencies = 1, ν_i = -467.77

Sum of electronic and zero-point Energies = -1997.171076 Hartree

Sum of electronic and thermal Energies = -1997.130657 Hartree

Sum of electronic and enthalpy Energies = -1997.129713 Hartree

Sum of electronic and thermal Free Energies = -1997.245847 Hartree

	Cartesian Coordinates				Cartesian Coordinates		
**Atom**	***X***	***Y***	***Z***	**Atom**	***X***	***Y***	***Z***
H	-6.321397	1.271105	0.138513	C	2.596077	4.205863	-0.225641
C	-5.551642	0.869116	0.801398	C	3.955655	4.476487	-0.039582
C	-5.785425	1.336493	2.266034	C	3.495217	6.836150	-0.285275
C	-5.578497	-0.668140	0.808816	H	1.439102	7.372760	-0.644943
H	-6.677238	1.968477	2.352424	H	2.245100	3.179998	-0.202844
H	-4.936625	1.914661	2.648612	H	3.845782	7.863309	-0.307620
H	-6.347039	-1.066489	0.138142	H	5.455912	6.017230	0.075499
C	-5.945864	0.039392	3.021525	C	1.577320	-5.226510	-0.523989
H	-6.103738	0.016143	4.096343	C	4.290714	-5.840225	-0.203358
C	-5.842505	-1.040346	2.247882	C	2.508724	-4.209960	-0.290472
H	-5.908677	-2.069224	2.587678	C	2.016166	-6.555538	-0.595975
C	-4.183850	-0.955318	0.287704	C	3.364956	-6.851448	-0.434158
C	-4.169885	1.091279	0.240448	C	3.866389	-4.506602	-0.131443
C	-3.211348	0.083810	0.756960	H	2.174897	-3.179566	-0.235494
C	-3.851652	-2.186360	-0.424052	H	1.295504	-7.346254	-0.776694
C	-3.244854	-4.627058	-1.713078	H	3.698908	-7.882861	-0.489647
C	-2.580806	-2.773130	-0.307400	H	5.343251	-6.073010	-0.080066
C	-4.809758	-2.859118	-1.205537	C	4.783216	-3.431170	0.090453
C	-4.508322	-4.060303	-1.834483	C	5.491560	-2.465312	0.263661
C	-2.269488	-3.976596	-0.945881	C	4.851987	3.380290	0.163956
H	-1.818580	-2.285113	0.286972	C	5.541033	2.397235	0.317116
H	-5.798727	-2.431727	-1.333984	C	6.239611	-1.259026	0.442014
H	-5.265371	-4.557251	-2.433543	C	7.633772	1.147722	0.765293
H	-3.004294	-5.561123	-2.209895	C	5.576431	-0.034845	0.310945

	Cartesian Coordinates				Cartesian Coordinates		
**Atom**	***X***	***Y***	***Z***	**Atom**	***X***	***Y***	***Z***
C	-3.796463	2.292904	-0.497466	C	7.609312	-1.269051	0.738124
C	-3.085344	4.672426	-1.825805	C	8.293351	-0.069121	0.897604
C	-4.702899	2.944966	-1.350719	C	6.264251	1.172436	0.469225
C	-2.527401	2.864852	-0.317648	H	4.516694	-0.021410	0.081202
C	-2.163240	4.040302	-0.980238	H	8.126773	-2.217156	0.840697
C	-4.349079	4.120224	-2.001479	H	9.354256	-0.082426	1.127157
H	-5.687699	2.519736	-1.517549	H	8.170391	2.082559	0.888981
H	-1.818542	2.385919	0.348059	O	-2.941327	0.224126	2.122008
H	-5.061859	4.606848	-2.660264	O	-1.941698	0.041964	0.132692
H	-2.802444	5.584221	-2.341503	C	-2.412664	-0.925493	2.746416
C	-0.944499	-4.502265	-0.814410	H	-3.044378	-1.805144	2.559730
C	0.198979	-4.876862	-0.684617	H	-2.404742	-0.720383	3.818647
C	-0.838956	4.553963	-0.801425	H	-1.390704	-1.133461	2.408574
C	0.304059	4.916984	-0.639391	C	-1.817616	0.023973	-1.279602
C	1.684113	5.242844	-0.445647	H	-2.786173	-0.017226	-1.785560
C	4.401802	5.804580	-0.068998	H	-1.233816	-0.854968	-1.570247
C	2.144563	6.566091	-0.475055	H	-1.293529	0.929593	-1.601482

cis-*par*-**CP3a**

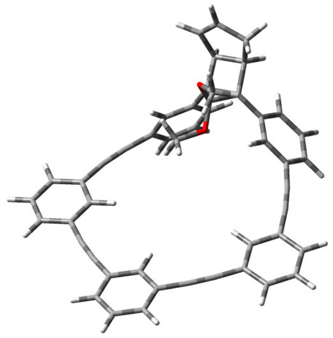

```
#p opt freq rwb97xd/6-31g(d)
```

Charge = 0, Multiplicity = 1

Number of Imaginary Frequencies = 0

Sum of electronic and zero-point Energies = -1997.189069 Hartree

Sum of electronic and thermal Energies = -1997.148331 Hartree

Sum of electronic and enthalpy Energies = -1997.147387 Hartree

Sum of electronic and thermal Free Energies = -1997.264806 Hartree

	Cartesian Coordinates				Cartesian Coordinates		
**Atom**	***X***	***Y***	***Z***	**Atom**	***X***	***Y***	***Z***
H	-6.357053	0.606759	0.279498	C	2.123461	4.209414	-0.373612
C	-5.641824	0.206824	1.000765	C	3.443618	4.613121	-0.149582
C	-6.107384	0.471568	2.449890	C	2.772333	6.908362	-0.496389
C	-5.441923	-1.319882	0.860647	H	0.689660	7.235610	-0.945334
H	-7.090946	0.957205	2.471332	H	1.869531	3.155988	-0.329430
H	-5.429670	1.126155	3.008430	H	3.025437	7.962949	-0.544289
H	-6.026027	-1.777210	0.053813	H	4.786634	6.293582	-0.038231
C	-6.163735	-0.917010	3.051045	C	1.877860	-5.033329	-0.739483
H	-6.464127	-1.082329	4.081999	C	4.538547	-5.600664	-0.068342
C	-5.779337	-1.879053	2.212771	C	2.771824	-3.998995	-0.445899
H	-5.722587	-2.934839	2.456699	C	2.327493	-6.358483	-0.694739
C	-3.988510	-1.108260	0.378406	C	3.650408	-6.630359	-0.359781
C	-4.202100	0.472321	0.499287	C	4.102057	-4.269988	-0.111020
C	-3.163397	-0.263547	1.302683	H	2.428590	-2.970877	-0.483096
C	-3.399759	-1.879265	-0.746352	H	1.638828	-7.165687	-0.921401
C	-2.258816	-3.297259	-2.887161	H	3.993869	-7.659610	-0.326005
C	-2.358863	-2.777021	-0.529363	H	5.569438	-5.818281	0.190788
C	-3.890758	-1.729484	-2.046718	C	4.962683	-3.161481	0.168634
C	-3.337110	-2.446723	-3.102259	C	5.586520	-2.144899	0.375168
C	-1.751775	-3.455948	-1.592608	C	4.428500	3.611964	0.125748
H	-1.985181	-2.937177	0.475045	C	5.194233	2.699518	0.340753
H	-4.699443	-1.029523	-2.234008	C	6.213851	-0.875920	0.583694
H	-3.728650	-2.317582	-4.106667	C	7.354985	1.653489	0.961208
H	-1.791025	-3.820353	-3.714744	C	5.454498	0.281032	0.379328

Atom	Cartesian Coordinates			Atom	Cartesian Coordinates		
	X	*Y*	*Z*		*X*	*Y*	*Z*
C	-3.895716	1.561410	-0.472145	C	7.552330	-0.756187	0.980208
C	-3.356206	3.625948	-2.315425	C	8.110386	0.504015	1.165205
C	-4.736731	1.792305	-1.567782	C	6.014812	1.548853	0.564872
C	-2.789719	2.395600	-0.309436	H	4.418672	0.193750	0.070232
C	-2.499091	3.408384	-1.231197	H	8.144872	-1.650978	1.139716
C	-4.475203	2.817408	-2.471372	H	9.147943	0.591462	1.472133
H	-5.608827	1.162228	-1.721989	H	7.794035	2.635015	1.106235
H	-2.115168	2.237210	0.520803	O	-3.240150	-0.424275	2.669238
H	-5.143149	2.977203	-3.312446	O	-1.876809	0.029144	0.857981
H	-3.133504	4.411095	-3.030544	C	-2.844610	0.728859	3.389097
C	-0.546541	-4.188989	-1.338621	H	-3.258289	1.639032	2.935671
C	0.531544	-4.671680	-1.073310	H	-1.752852	0.823237	3.432022
C	-1.284152	4.149002	-1.063365	H	-3.237537	0.614528	4.401437
C	-0.207343	4.672272	-0.884744	C	-0.814930	-0.765223	1.362312
C	1.125419	5.146370	-0.661315	H	-0.344976	-1.311914	0.539577
C	3.763598	5.976146	-0.211210	H	-1.162348	-1.468694	2.124055
C	1.460452	6.505225	-0.722375	H	-0.075751	-0.095491	1.814260

cis-*twi*-**CP3a**

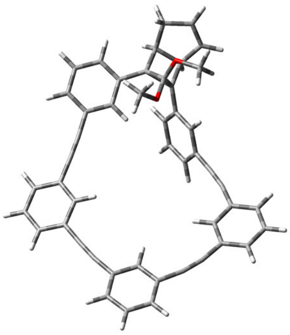

#p opt freq rwb97xd/6-31g(d)

Charge = 0, Multiplicity = 1

Number of Imaginary Frequencies = 0

Sum of electronic and zero-point Energies = -1997.201114 Hartree

Sum of electronic and thermal Energies = -1997.160386 Hartree

Sum of electronic and enthalpy Energies = -1997.159442 Hartree

Sum of electronic and thermal Free Energies = -1997.276710 Hartree

	Cartesian Coordinates				Cartesian Coordinates		
**Atom**	***X***	***Y***	***Z***	**Atom**	***X***	***Y***	***Z***
C	-6.207717	-0.130368	0.178274	C	3.880766	5.614121	0.489029
C	-4.795311	0.283427	-0.275468	C	2.344548	3.776251	0.143345
C	-5.633356	-1.416024	0.819892	C	1.502390	5.977923	0.674353
C	-4.203265	-1.048246	0.345917	C	2.804273	6.459918	0.728210
H	-6.580295	0.576472	0.923761	C	3.656634	4.262624	0.194560
H	-5.652316	-1.415852	1.915030	H	2.167537	2.731226	-0.086609
C	-3.016601	-1.249326	1.213667	H	0.661631	6.638883	0.857517
C	-0.851814	-1.690920	2.934060	H	2.981788	7.506076	0.957364
C	-2.008160	-2.142951	0.857162	H	4.898153	5.989015	0.528904
C	-2.926434	-0.573511	2.433790	C	5.663731	-3.174195	-0.360239
C	-1.852842	-0.796408	3.289362	C	6.355042	-2.191262	-0.507895
C	-0.920489	-2.367544	1.708202	C	4.745456	3.367663	-0.052218
H	-2.052408	-2.652056	-0.099630	C	5.631922	2.571281	-0.264302
H	-3.699313	0.141930	2.702717	C	-4.127234	-0.771566	-1.121764
H	-1.790718	-0.262660	4.232689	O	-4.850705	-1.442692	-2.084077
H	-0.005074	-1.863762	3.590269	O	-2.832995	-0.480012	-1.522751
C	-4.327176	1.690523	-0.192240	C	-2.764258	0.260309	-2.733016
C	-3.566618	4.393362	-0.105572	H	-3.059303	-0.356037	-3.588885
C	-5.272621	2.711081	-0.346166	H	-1.723123	0.568036	-2.840413
C	-2.989647	2.040976	0.019364	H	-3.403588	1.149139	-2.693220
C	-2.602607	3.386787	0.049565	C	-4.381012	-2.758736	-2.309145
C	-4.896105	4.048814	-0.300198	H	-3.369984	-2.753005	-2.733639
H	-6.314582	2.452868	-0.514349	H	-5.074631	-3.221073	-3.013222
H	-2.239921	1.268061	0.140221	H	-4.378137	-3.335393	-1.375215

	Cartesian Coordinates				Cartesian Coordinates		
**Atom**	***X***	***Y***	***Z***	**Atom**	***X***	***Y***	***Z***
H	-5.644504	4.825726	-0.424173	C	6.564723	1.509759	-0.490183
H	-3.260009	5.433829	-0.078090	C	8.291616	-0.659471	-0.914102
C	-1.228547	3.759250	0.207503	C	6.090620	0.198198	-0.413508
C	-0.082752	4.140049	0.297462	C	7.917719	1.729371	-0.782063
C	0.150761	-3.232220	1.311001	C	8.765658	0.646720	-0.991593
C	1.097349	-3.909134	0.977964	C	6.942742	-0.891696	-0.621475
C	2.330432	-4.506969	0.557328	H	5.045030	0.021363	-0.186829
C	4.807962	-5.527261	-0.274945	H	8.294935	2.744819	-0.843790
C	3.403032	-3.650495	0.300668	H	9.812538	0.823387	-1.218094
C	2.507343	-5.886554	0.393578	H	8.959104	-1.499195	-1.076813
C	3.740575	-6.382687	-0.018809	C	-7.311621	-0.567817	-0.809223
C	4.642633	-4.146566	-0.116292	H	-7.071300	-0.308830	-1.845894
H	3.271219	-2.581377	0.424513	H	-8.278296	-0.106362	-0.571799
H	1.680341	-6.560674	0.590856	C	-6.439996	-2.534915	0.224915
H	3.871622	-7.453166	-0.143085	H	-6.298098	-3.580314	0.481150
H	5.765781	-5.921540	-0.597984	C	-7.356149	-2.070256	-0.625658
C	1.260657	4.627726	0.378732	H	-8.061984	-2.692099	-1.169600

trans-**TS3a**

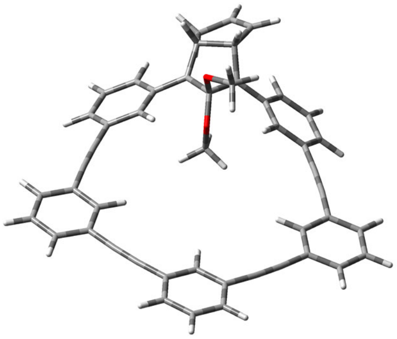

`#p opt=(ts,calcfc) freq uwb97xd/6-31g(d) guess=(mix,always)`

Charge = 0, Multiplicity = 1

Number of Imaginary Frequencies = 1, ν_i = -441.35

Sum of electronic and zero-point Energies = -1997.164973 Hartree

Sum of electronic and thermal Energies = -1997.124665 Hartree

Sum of electronic and enthalpy Energies = -1997.123721 Hartree

Sum of electronic and thermal Free Energies = -1997.239560 Hartree

	**Cartesian Coordinates**				**Cartesian Coordinates**		
**Atom**	***X***	***Y***	***Z***	**Atom**	***X***	***Y***	***Z***
H	5.655601	1.491712	1.989023	C	-2.661400	4.188330	-0.180629
C	5.472147	0.833522	1.127895	C	-4.030313	4.453453	-0.069195
C	6.732077	0.833445	0.208817	C	-3.567483	6.815493	-0.284382
C	5.293915	-0.656361	1.504333	H	-1.496894	7.360933	-0.528004
H	7.374251	1.702587	0.388428	H	-2.307967	3.163832	-0.140542
H	6.453249	0.849861	-0.852862	H	-3.920526	7.841285	-0.324109
H	5.035593	-0.794154	2.564097	H	-5.541522	5.987298	-0.034089
C	7.410319	-0.477272	0.511937	C	-1.633474	-5.220362	-0.454218
H	8.411344	-0.713873	0.163374	C	-4.353690	-5.857904	-0.262420
C	6.608924	-1.305529	1.175888	C	-2.583927	-4.212111	-0.266961
H	6.850632	-2.322815	1.466096	C	-2.056626	-6.553329	-0.545100
C	4.066133	-0.938164	0.664659	C	-3.408964	-6.861032	-0.447383
C	4.132310	1.111279	0.491509	C	-3.944997	-4.520591	-0.172352
C	3.095479	0.155150	0.976231	H	-2.262460	-3.178649	-0.197994
C	3.780874	-2.168025	-0.072425	H	-1.321249	-7.337821	-0.690069
C	3.226080	-4.575179	-1.434631	H	-3.730721	-7.895436	-0.517233
C	2.506156	-2.751579	-0.018148	H	-5.408788	-6.099978	-0.189122
C	4.768109	-2.813849	-0.838300	C	-4.878965	-3.451606	0.004077
C	4.491064	-4.002658	-1.501785	C	-5.596968	-2.487190	0.142017
C	2.220445	-3.942975	-0.692234	C	-4.934054	3.355123	0.083599
H	1.718789	-2.270389	0.549021	C	-5.633475	2.374219	0.198753
H	5.752535	-2.367482	-0.919756	C	-6.350714	-1.279572	0.283122
H	5.268051	-4.483182	-2.088649	C	-7.750885	1.131714	0.537561
H	3.005544	-5.498729	-1.959812	C	-5.677440	-0.057631	0.187219

	Cartesian Coordinates				Cartesian Coordinates		
**Atom**	***X***	***Y***	***Z***	**Atom**	***X***	***Y***	***Z***
C	3.799113	2.361816	-0.194824	C	-7.733651	-1.285215	0.509116
C	3.127346	4.852394	-1.356993	C	-8.420635	-0.082903	0.634747
C	4.756818	3.134828	-0.877304	C	-6.367951	1.151943	0.311655
C	2.498680	2.888306	-0.090946	H	-4.607376	-0.047806	0.011536
C	2.155323	4.112067	-0.670914	H	-8.259015	-2.231540	0.584272
C	4.422065	4.358180	-1.445576	H	-9.491884	-0.092569	0.810020
H	5.776391	2.784981	-0.969032	H	-8.289849	2.068314	0.634886
H	1.745005	2.340435	0.461123	O	1.879163	0.109309	0.263888
H	5.182360	4.930752	-1.968207	O	2.744898	0.364118	2.317415
H	2.861614	5.802700	-1.808290	C	1.872746	0.054113	-1.153501
C	0.892889	-4.473889	-0.624549	H	1.428200	0.975181	-1.544686
C	-0.252161	-4.858463	-0.549071	H	1.268114	-0.802869	-1.464328
C	0.805938	4.577700	-0.562227	H	2.877247	-0.054077	-1.571389
C	-0.351900	4.916015	-0.464380	C	2.047752	-0.709581	2.915515
C	-1.743070	5.229655	-0.347317	H	1.046047	-0.827786	2.487782
C	-4.480081	5.779666	-0.120604	H	1.962196	-0.471837	3.977577
C	-2.207451	6.551115	-0.399300	H	2.598440	-1.655641	2.804641

trans-par-**CP3a**

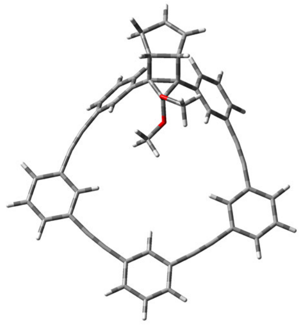

```
#p opt freq rwb97xd/6-31g(d)
```

Charge = 0, Multiplicity = 1

Number of Imaginary Frequencies = 0

Sum of electronic and zero-point Energies = -1997.201345 Hartree

Sum of electronic and thermal Energies = -1997.160596 Hartree

Sum of electronic and enthalpy Energies = -1997.159652 Hartree

Sum of electronic and thermal Free Energies = -1997.277163 Hartree

Atom	Cartesian Coordinates			Atom	Cartesian Coordinates		
	X	***Y***	***Z***		***X***	***Y***	***Z***
H	-4.919651	-1.597112	2.629257	C	2.621818	-4.100421	-0.402089
C	-5.120583	-1.124152	1.666665	C	3.970314	-4.378057	-0.158423
C	-6.372496	-1.702843	0.979435	C	3.491019	-6.736526	-0.374368
C	-5.286741	0.431194	1.682242	H	1.443236	-7.261771	-0.797699
H	-6.960754	-2.316127	1.673851	H	2.282032	-3.070561	-0.416967
H	-6.118490	-2.355027	0.134632	H	3.830934	-7.767446	-0.363372
H	-5.275370	0.937476	2.654303	H	5.446917	-5.933683	0.043804
C	-7.145421	-0.482027	0.547444	C	1.254678	5.175678	-0.654067
H	-8.080037	-0.548287	-0.002217	C	3.936836	5.916056	-0.322358
C	-6.569712	0.656338	0.931524	C	2.232962	4.205736	-0.410547
H	-6.957592	1.650278	0.731327	C	1.631687	6.522415	-0.730562
C	-3.996992	0.602887	0.847983	C	2.965369	6.881022	-0.563546
C	-3.856650	-0.964265	0.803136	C	3.574481	4.564406	-0.245640
C	-2.879158	-0.109254	1.561061	H	1.945763	3.161450	-0.354672
C	-3.764167	1.650637	-0.186716	H	0.876786	7.278589	-0.919288
C	-3.290300	3.640845	-2.124624	H	3.251283	7.926583	-0.623278
C	-2.671751	2.510840	-0.077916	H	4.976529	6.199225	-0.195355
C	-4.625603	1.813589	-1.278272	C	4.535026	3.529180	-0.013182
C	-4.397879	2.807876	-2.225112	C	5.274429	2.588324	0.169305
C	-2.409421	3.484255	-1.049028	C	4.856966	-3.276395	0.060672
H	-1.977638	2.396490	0.744417	C	5.509972	-2.270282	0.223632
H	-5.486063	1.161348	-1.382532	C	6.064703	1.410855	0.360855
H	-5.081595	2.919961	-3.061310	C	7.544101	-0.941304	0.717209
H	-3.091446	4.394860	-2.879319	C	5.453916	0.161258	0.213306

Atom	Cartesian Coordinates			Atom	Cartesian Coordinates		
	X	*Y*	*Z*		*X*	*Y*	*Z*
C	-3.454202	-1.840470	-0.325496	C	7.425764	1.473067	0.689214
C	-2.615650	-3.443920	-2.475285	C	8.152171	0.300474	0.864324
C	-4.091197	-1.761254	-1.567496	C	6.184095	-1.018958	0.388837
C	-2.421029	-2.759165	-0.166897	H	4.401612	0.106813	-0.043306
C	-1.961931	-3.530399	-1.240863	H	7.903480	2.440375	0.804549
C	-3.688001	-2.572047	-2.624356	H	9.206105	0.355100	1.118555
H	-4.897195	-1.047412	-1.706001	H	8.113792	-1.854485	0.854084
H	-1.932545	-2.859266	0.795359	O	-1.638141	0.125604	0.990511
H	-4.193099	-2.499555	-3.582691	O	-2.853216	-0.203646	2.942060
H	-2.264022	-4.038537	-3.312047	C	-0.559635	-0.694542	1.413551
C	-1.189831	4.229017	-0.945291	H	-0.836550	-1.314266	2.270941
C	-0.100057	4.740260	-0.816920	H	0.264009	-0.036648	1.708102
C	-0.751227	-4.278280	-1.070428	H	-0.230775	-1.330403	0.585959
C	0.340902	-4.763151	-0.875776	C	-2.481452	1.011105	3.568494
C	1.704889	-5.130689	-0.633680	H	-1.429494	1.255527	3.378086
C	4.401830	-5.711130	-0.144626	H	-2.633300	0.870936	4.639997
C	2.149848	-6.458077	-0.618883	H	-3.106150	1.841665	3.213610

trans-*twi*-**CP3a**

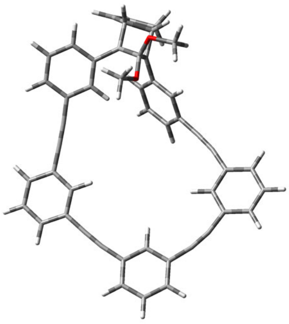

#p opt freq rwb97xd/6-31g(d)

Charge = 0, Multiplicity = 1

Number of Imaginary Frequencies = 0

Sum of electronic and zero-point Energies = -1997.210253 Hartree

Sum of electronic and thermal Energies = -1997.169517 Hartree

Sum of electronic and enthalpy Energies = -1997.168573 Hartree

Sum of electronic and thermal Free Energies = -1997.285731 Hartree

	Cartesian Coordinates				Cartesian Coordinates		
**Atom**	***X***	***Y***	***Z***	**Atom**	***X***	***Y***	***Z***
C	6.211790	-0.574522	0.298681	C	-1.121912	5.955067	-0.718395
C	4.813767	-0.028317	0.631584	C	-2.394346	6.495374	-0.856899
C	5.578536	-1.907370	-0.220578	C	-3.378925	4.355549	-0.317010
C	4.170875	-1.324808	0.034575	H	-1.982420	2.764893	0.084785
C	3.012126	-1.472216	-0.878673	H	-0.241677	6.570974	-0.870600
C	0.879621	-1.786842	-2.664184	H	-2.508786	7.541948	-1.121557
C	1.958635	-2.330051	-0.567923	H	-4.516107	6.128027	-0.762813
C	2.983701	-0.766044	-2.085204	C	-5.812305	-2.956471	0.279005
C	1.926166	-0.928468	-2.974148	C	-6.455240	-1.934887	0.373433
C	0.885255	-2.490140	-1.451202	C	-4.521913	3.520390	-0.108269
H	1.953858	-2.857566	0.380229	C	-5.457994	2.775286	0.075042
H	3.795786	-0.080080	-2.312207	C	4.066748	-1.016908	1.495436
H	1.912182	-0.374048	-3.907435	O	4.776484	-1.729955	2.445532
H	0.043935	-1.909613	-3.345451	O	2.795183	-0.657689	1.894873
C	4.431669	1.402453	0.532883	C	2.767437	0.123559	3.081705
C	3.802203	4.138807	0.460412	H	3.069816	-0.471022	3.950622
C	5.405413	2.373359	0.793910	H	1.735182	0.455136	3.201847
C	3.131337	1.819718	0.233626	H	3.423149	0.997087	2.998133
C	2.808273	3.182510	0.208167	C	4.199381	-2.994919	2.718153
C	5.094877	3.727906	0.751676	H	3.207882	-2.891703	3.174047
H	6.414795	2.062264	1.047493	H	4.871011	-3.502838	3.412232
H	2.356724	1.086164	0.043414	H	4.104678	-3.590128	1.799222
H	5.864672	4.465922	0.956154	H	5.788945	-2.798426	0.374591
H	3.546292	5.192937	0.435314	H	6.910890	-0.630272	1.139768

Atom	Cartesian Coordinates			Atom	Cartesian Coordinates		
	X	*Y*	*Z*		*X*	*Y*	*Z*
C	1.467876	3.621571	-0.041326	C	-6.457397	1.770678	0.274942
C	0.349909	4.055828	-0.207049	C	-8.321056	-0.290997	0.654762
C	-0.236973	-3.308232	-1.099126	C	-6.054089	0.433782	0.246407
C	-1.227988	-3.939045	-0.807214	C	-7.808461	2.070319	0.496106
C	-2.508389	-4.469950	-0.441845	C	-8.724588	1.040594	0.684296
C	-5.073241	-5.351233	0.277380	C	-6.974783	-0.603057	0.433036
C	-3.548711	-3.557250	-0.255602	H	-5.010341	0.195028	0.074682
C	-2.762040	-5.835770	-0.264611	H	-8.130919	3.106021	0.520170
C	-4.038094	-6.262676	0.091260	H	-9.769587	1.279274	0.856007
C	-4.831251	-3.983615	0.104653	H	-9.041410	-1.089131	0.800471
H	-3.357398	-2.498453	-0.389940	C	6.010035	-2.077138	-1.687157
H	-1.960892	-6.553471	-0.407332	H	5.160273	-2.280774	-2.349463
H	-4.228636	-7.322862	0.226368	H	6.701446	-2.922056	-1.801834
H	-6.064754	-5.691974	0.556725	C	6.814055	0.021802	-0.947593
C	-0.961756	4.603721	-0.376436	H	7.280753	1.001440	-0.962008
C	-3.521562	5.707403	-0.657124	C	6.693106	-0.772753	-2.009382
C	-2.096455	3.810195	-0.180922	H	7.057388	-0.534694	-3.004433

AZ3b

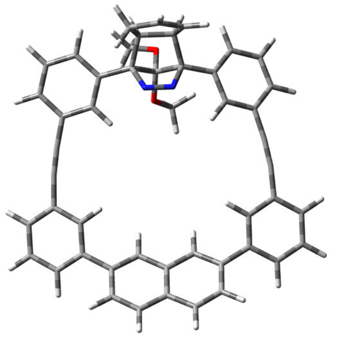

```
#p opt freq rwb97xd/6-31g(d)
```

Charge = 0, Multiplicity = 1

Number of Imaginary Frequencies = 0

Sum of electronic and zero-point Energies = -2107.993627 Hartree

Sum of electronic and thermal Energies = -2107.953553 Hartree

Sum of electronic and enthalpy Energies = -2107.952609 Hartree

Sum of electronic and thermal Free Energies = -2108.065664 Hartree

	Cartesian Coordinates				Cartesian Coordinates		
Atom	*X*	*Y*	*Z*	Atom	*X*	*Y*	*Z*
C	3.859114	0.932288	0.492161	C	-2.453759	-4.831857	-0.231667
C	3.585241	-0.116696	-0.639010	C	-5.243355	-4.709066	-0.207252
C	3.725109	-1.308132	0.370735	C	-3.206585	-5.980337	-0.503496
C	5.177607	-1.067449	0.887308	C	-3.115382	-3.635743	0.068018
H	5.881815	-1.481205	0.161306	C	-4.507933	-3.550344	0.066037
C	5.269245	0.486173	0.958098	C	-4.594905	-5.909980	-0.481477
H	6.012164	0.854321	0.247953	H	-2.702311	-6.913411	-0.732275
N	2.925881	0.418142	1.540858	H	-2.519257	-2.763119	0.313724
N	2.855041	-0.819565	1.466715	H	-5.180443	-6.798578	-0.697194
O	2.313279	0.078638	-1.155431	H	-6.328578	-4.666077	-0.232085
O	4.550922	-0.235899	-1.627108	C	-1.982751	4.914850	-0.495339
C	1.958501	-0.687928	-2.300247	C	-4.770649	5.027107	-0.628937
H	0.872436	-0.791322	-2.272926	C	-2.761461	3.804755	-0.148623
H	2.415123	-1.679113	-2.295390	C	-2.617303	6.093982	-0.904200
H	2.248005	-0.165637	-3.219490	C	-4.005546	6.140931	-0.963354
C	4.747222	0.915612	-2.436479	C	-4.154270	3.839136	-0.221078
H	3.810373	1.458260	-2.595145	H	-2.259751	2.908531	0.201518
H	5.136498	0.561717	-3.392994	H	-2.021818	6.960874	-1.171061
H	5.472861	1.596475	-1.977643	H	-4.498225	7.053604	-1.284764
C	3.332529	-2.682991	-0.087670	H	-5.853258	5.072988	-0.707919
C	2.474689	-5.161672	-1.084660	C	-4.928590	2.621864	0.129464
C	4.226445	-3.532945	-0.742440	H	-3.540470	1.293598	-0.807918
C	2.007525	-3.087901	0.066991	C	-4.450656	1.379745	-0.219985
C	1.563527	-4.315002	-0.440180	C	-6.816201	1.567625	1.241789

	Cartesian Coordinates				Cartesian Coordinates		
**Atom**	***X***	***Y***	***Z***	**Atom**	***X***	***Y***	***Z***
C	3.798331	-4.766188	-1.227183	C	-5.107715	0.191812	0.178671
H	5.258107	-3.232014	-0.890964	C	-6.145563	2.700959	0.861514
H	1.304772	-2.433815	0.571268	C	-6.309589	0.279308	0.928240
H	4.503964	-5.419588	-1.731201	C	-4.568968	-1.081319	-0.121232
H	2.135663	-6.114646	-1.477518	H	-6.526748	3.676519	1.150003
C	3.609303	2.390396	0.228093	H	-7.846829	-0.869901	1.930649
C	3.037918	5.093817	-0.267647	H	-7.735615	1.645562	1.816375
C	2.291960	2.818279	0.068639	C	-5.156342	-2.241050	0.330742
C	4.636216	3.333097	0.152491	H	-3.662964	-1.127698	-0.719680
C	4.350046	4.672818	-0.099229	C	-6.367050	-2.144353	1.071168
C	1.994014	4.163578	-0.178291	H	-6.834021	-3.051454	1.444290
H	1.484637	2.099030	0.140101	C	-6.928491	-0.925851	1.351784
H	5.669038	3.029885	0.295713	C	5.644905	0.846090	2.415604
H	5.159303	5.394512	-0.155308	H	4.896159	1.494057	2.886267
H	2.811057	6.137773	-0.457940	H	6.600535	1.382391	2.464533
C	0.173491	-4.639116	-0.340129	C	5.726389	-0.494614	3.102799
C	-1.023541	-4.809834	-0.278742	H	5.959148	-0.586406	4.159139
C	0.623204	4.548246	-0.319752	C	5.470621	-1.518138	2.291477
C	-0.559704	4.786956	-0.416446	H	5.457493	-2.563569	2.581404

S-**DR3b**

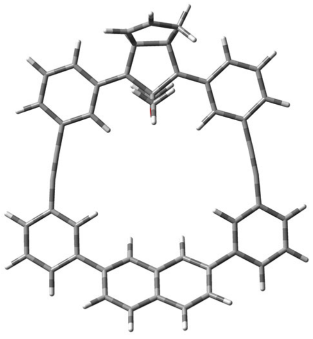

```
#p opt freq uwb97xd/6-31g(d) guess=(mix,always)
```

Charge = 0, Multiplicity = 1, $\langle S^2 \rangle$ = 1.0222

Number of Imaginary Frequencies = 0

Sum of electronic and zero-point Energies = -1998.515907 Hartree

Sum of electronic and thermal Energies = -1998.476355 Hartree

Sum of electronic and enthalpy Energies = -1998.475411 Hartree

Sum of electronic and thermal Free Energies = -1998.587650 Hartree

	Cartesian Coordinates				Cartesian Coordinates		
**Atom**	***X***	***Y***	***Z***	**Atom**	***X***	***Y***	***Z***
C	-4.152604	-1.245639	-0.074232	C	2.797151	-6.001621	-0.704483
C	-3.230154	-0.043701	-0.119129	C	4.187256	-6.001320	-0.726243
C	-4.184433	1.134802	-0.086906	C	4.235781	-3.655780	-0.120821
C	-5.618148	0.711861	0.014813	H	2.297712	-2.773987	0.201233
C	-5.590433	-0.842329	0.077960	H	2.239670	-6.903301	-0.935876
C	-3.769488	2.497393	-0.200943	H	4.719861	-6.912693	-0.980760
C	-2.977557	5.201227	-0.438049	H	5.989087	-4.852809	-0.495358
C	-4.722807	3.539972	-0.313920	C	4.952758	-2.389207	0.170943
C	-2.401168	2.856598	-0.211894	H	3.504665	-1.177187	-0.832117
C	-2.007912	4.190407	-0.328909	C	4.414361	-1.190802	-0.237672
C	-4.327184	4.863148	-0.430246	C	6.779537	-1.187668	1.235419
H	-5.781857	3.302440	-0.318685	C	5.002909	0.046076	0.113499
H	-1.647272	2.084217	-0.125174	C	6.169765	-2.370875	0.907105
H	-5.077738	5.642920	-0.518468	C	6.204778	0.057158	0.867582
H	-2.664546	6.236087	-0.528211	C	4.386075	1.271583	-0.228898
C	-3.710802	-2.599588	-0.169142	H	6.600712	-3.310527	1.241230
C	-2.860053	-5.289472	-0.360957	H	7.670229	1.333099	1.823808
C	-2.334528	-2.927015	-0.207909	H	7.700260	-1.191543	1.813270
C	-4.641319	-3.667143	-0.231305	C	4.896341	2.479129	0.188863
C	-4.216831	-4.982973	-0.326266	H	3.477609	1.241013	-0.824543
C	-1.912508	-4.253659	-0.301122	C	6.112899	2.483338	0.926040
H	-1.597790	-2.134907	-0.160242	H	6.521733	3.430064	1.267847
H	-5.705862	-3.456252	-0.214990	C	6.750045	1.312191	1.245465
H	-4.950377	-5.782135	-0.375688	O	-2.376138	-0.039689	-1.247213

	Cartesian Coordinates				Cartesian Coordinates		
**Atom**	***X***	***Y***	***Z***	**Atom**	***X***	***Y***	***Z***
H	-2.524616	-6.318676	-0.433287	O	-2.265394	-0.024428	0.916744
C	-0.510983	-4.541958	-0.333177	C	-3.029485	-0.051428	-2.494467
C	0.682699	-4.741573	-0.360329	H	-3.647034	-0.951101	-2.623997
C	-0.613067	4.510836	-0.338552	H	-3.658759	0.838594	-2.634251
C	0.576054	4.737426	-0.350748	H	-2.241067	-0.050390	-3.248686
C	2.002240	4.852755	-0.361909	C	-2.789553	-0.005444	2.224379
C	4.794243	4.934714	-0.405736	H	-3.397803	0.891886	2.406026
C	2.663035	6.048024	-0.670917	H	-1.928500	0.005402	2.894381
C	2.756711	3.713072	-0.064010	H	-3.401972	-0.892365	2.433390
C	4.151631	3.731250	-0.095954	H	-6.175776	1.047546	-0.869589
C	4.052849	6.079505	-0.684947	H	-6.208598	-1.289568	-0.715065
H	2.086383	6.938303	-0.899459	C	-6.168768	-1.158801	1.443660
H	2.232264	2.803662	0.209834	H	-6.218923	-2.173456	1.825962
H	4.565957	7.004512	-0.930177	C	-6.556942	-0.068718	2.098073
H	5.879038	4.970461	-0.453454	H	-6.982916	-0.064056	3.097026
C	2.111044	-4.823965	-0.383367	C	-6.346716	1.194201	1.308872
C	4.903992	-4.842052	-0.442385	H	-5.749474	1.929407	1.861612
C	2.841012	-3.669373	-0.081971	H	-7.302386	1.680908	1.075259

T-**DR3b**

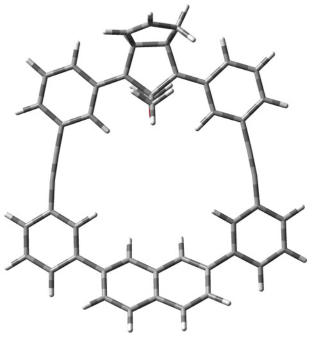

```
#p opt freq uwb97xd/6-31g(d)
```

Charge = 0, Multiplicity = 3, <S^2> = 2.0914

Number of Imaginary Frequencies = 0

Sum of electronic and zero-point Energies = -1998.513107 Hartree

Sum of electronic and thermal Energies = -1998.473501 Hartree

Sum of electronic and enthalpy Energies = -1998.472557 Hartree

Sum of electronic and thermal Free Energies = -1998.585968 Hartree

	Cartesian Coordinates				Cartesian Coordinates		
**Atom**	***X***	***Y***	***Z***	**Atom**	***X***	***Y***	***Z***
C	-4.155204	-1.260455	-0.074361	C	2.807230	-5.997217	-0.708717
C	-3.239595	-0.047498	-0.116499	C	4.197304	-5.996275	-0.732923
C	-4.191179	1.139251	-0.086693	C	4.245805	-3.651295	-0.125654
C	-5.625230	0.707063	-0.004411	H	2.307962	-2.770668	0.200651
C	-5.593411	-0.851169	0.070928	H	2.249771	-6.898983	-0.939843
C	-3.774428	2.496211	-0.199750	H	4.729841	-6.907217	-0.989129
C	-2.976686	5.200194	-0.437752	H	5.999079	-4.847146	-0.504102
C	-4.726047	3.542468	-0.316266	C	4.962397	-2.384512	0.166069
C	-2.404010	2.854423	-0.208297	H	3.512282	-1.173082	-0.834674
C	-2.009019	4.187354	-0.325438	C	4.422527	-1.186279	-0.241055
C	-4.327351	4.863994	-0.433182	C	6.788924	-1.182097	1.230001
H	-5.785529	3.306902	-0.323834	C	5.009981	0.050849	0.110934
H	-1.651085	2.081335	-0.120203	C	6.180119	-2.365643	0.901038
H	-5.076200	5.645122	-0.524346	C	6.212395	0.062505	0.864124
H	-2.661660	6.234385	-0.528077	C	4.391260	1.275948	-0.229370
C	-3.708648	-2.607998	-0.161805	H	6.612352	-3.305191	1.233797
C	-2.847055	-5.297155	-0.342358	H	7.676852	1.339201	1.820885
C	-2.329728	-2.931300	-0.205268	H	7.710174	-1.185574	1.807018
C	-4.635376	-3.681910	-0.213521	C	4.899972	2.483625	0.189870
C	-4.205462	-4.995586	-0.303119	H	3.482447	1.244933	-0.824462
C	-1.903553	-4.256691	-0.292700	C	6.117125	2.488509	0.926073
H	-1.595420	-2.136555	-0.165976	H	6.524856	3.435319	1.268960
H	-5.700708	-3.475141	-0.192894	C	6.756230	1.317800	1.243254
H	-4.935755	-5.798236	-0.344527	O	-2.391472	-0.041323	-1.245914

	Cartesian Coordinates				Cartesian Coordinates		
Atom	*X*	*Y*	*Z*	Atom	*X*	*Y*	*Z*
H	-2.507699	-6.325330	-0.410253	O	-2.281694	-0.027449	0.921444
C	-0.501180	-4.540628	-0.328978	C	-3.047444	-0.053919	-2.493011
C	0.692694	-4.738787	-0.359319	H	-3.663447	-0.954683	-2.619935
C	-0.613836	4.506561	-0.333189	H	-3.676502	0.836222	-2.630848
C	0.575059	4.734519	-0.344507	H	-2.260079	-0.052439	-3.248120
C	2.001024	4.852454	-0.355705	C	-2.809612	-0.009631	2.228645
C	4.792974	4.940034	-0.400680	H	-3.422288	0.885110	2.406238
C	2.659399	6.049471	-0.663249	H	-1.950023	0.006191	2.900211
C	2.757968	3.713933	-0.059733	H	-3.417029	-0.900106	2.435766
C	4.152844	3.734871	-0.092411	H	-6.170006	1.038971	-0.898293
C	4.049140	6.083758	-0.677805	H	-6.212061	-1.308083	-0.715741
H	2.080883	6.938929	-0.890287	C	-6.171764	-1.156026	1.439871
H	2.235478	2.803105	0.213121	H	-6.214415	-2.166755	1.833330
H	4.560273	7.010166	-0.921889	C	-6.575835	-0.062337	2.078035
H	5.877674	4.977977	-0.448905	H	-7.012944	-0.051095	3.072042
C	2.121046	-4.820182	-0.385384	C	-6.375487	1.193783	1.275924
C	4.914065	-4.836949	-0.449320	H	-5.794532	1.944034	1.825433
C	2.851106	-3.665567	-0.084384	H	-7.335902	1.663718	1.026942

cis-TS3b

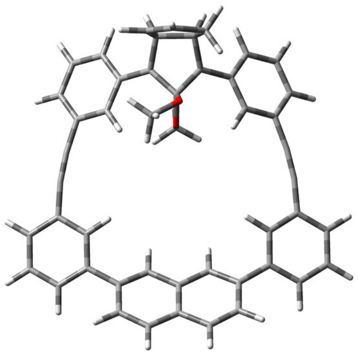

```
#p opt=(ts,calcfc) freq uwb97xd/6-31g(d) guess=(mix,always)
```

Charge = 0, Multiplicity = 1

Number of Imaginary Frequencies = 1, v_i = -490.96

Sum of electronic and zero-point Energies = -1998.493257 Hartree

Sum of electronic and thermal Energies = -1998.454537 Hartree

Sum of electronic and enthalpy Energies = -1998.453593 Hartree

Sum of electronic and thermal Free Energies = -1998.563371 Hartree

	Cartesian Coordinates				Cartesian Coordinates		
**Atom**	***X***	***Y***	***Z***	**Atom**	***X***	***Y***	***Z***
C	-4.022174	-0.954740	0.292501	C	2.527730	-6.018799	-0.959816
C	-3.039366	0.078597	0.750754	C	3.912604	-6.039526	-0.834822
C	-4.010197	1.085615	0.258639	C	3.929047	-3.712233	-0.159266
C	-5.378318	0.861901	0.852002	H	1.980201	-2.810416	-0.013009
C	-5.404758	-0.674958	0.848935	H	1.986600	-6.904479	-1.276048
C	-3.652251	2.297942	-0.472043	H	4.457262	-6.951667	-1.059150
C	-2.958889	4.693103	-1.786397	H	5.695575	-4.927146	-0.385132
C	-4.578672	2.972213	-1.285962	C	4.628602	-2.462111	0.231087
C	-2.374816	2.858313	-0.322822	H	3.312387	-1.210479	-0.897428
C	-2.016572	4.038022	-0.982730	C	4.155157	-1.248424	-0.212223
C	-4.234023	4.155882	-1.926843	C	6.356512	-1.308362	1.494507
H	-5.572213	2.559088	-1.429850	C	4.728616	-0.026869	0.211423
H	-1.649193	2.364588	0.313077	C	5.762909	-2.475872	1.089233
H	-4.963268	4.660192	-2.553592	C	5.847505	-0.048328	1.083790
H	-2.683624	5.608253	-2.300296	C	4.179961	1.215403	-0.183654
C	-3.701891	-2.188414	-0.423020	H	6.140845	-3.427585	1.452106
C	-3.104816	-4.632000	-1.715535	H	7.236713	1.187021	2.194608
C	-2.423572	-2.762834	-0.340207	H	7.212800	-1.336735	2.163483
C	-4.675332	-2.877247	-1.170896	C	4.676599	2.408801	0.288539
C	-4.378604	-4.080335	-1.799143	H	3.337541	1.210133	-0.870211
C	-2.114409	-3.964925	-0.983923	C	5.809893	2.379481	1.147649
H	-1.649223	-2.263285	0.228136	H	6.205897	3.314563	1.533719
H	-5.672793	-2.462551	-1.272942	C	6.380549	1.191328	1.524895
H	-5.148160	-4.590354	-2.370686	O	-1.782607	0.041431	0.098208

	Cartesian Coordinates				Cartesian Coordinates		
Atom	*X*	*Y*	*Z*	Atom	*X*	*Y*	*Z*
H	-2.869181	-5.564971	-2.216765	O	-2.738070	0.210411	2.109897
C	-0.767406	-4.441843	-0.899155	C	-1.687733	0.031417	-1.316175
C	0.407659	-4.717492	-0.802488	H	-2.666643	-0.001055	-1.802420
C	-0.668300	4.500837	-0.851935	H	-1.165480	0.936594	-1.642819
C	0.507252	4.763167	-0.728420	H	-1.115432	-0.849337	-1.624616
C	1.927904	4.859973	-0.580877	C	-2.195892	-0.943114	2.715241
C	4.709648	4.862457	-0.327517	H	-2.165268	-0.745183	3.788429
C	2.651437	6.031079	-0.833061	H	-1.181121	-1.148376	2.354638
C	2.613160	3.704741	-0.187813	H	-2.830900	-1.821861	2.536051
C	4.003265	3.681377	-0.074587	H	-6.163272	1.268472	0.210087
C	4.035440	6.023214	-0.696823	H	-6.187895	-1.068463	0.192409
H	2.129470	6.933395	-1.134090	C	-5.635010	-1.057989	2.290835
H	2.036043	2.814431	0.039469	H	-5.693180	-2.089296	2.624687
H	4.598664	6.929860	-0.896288	C	-5.719753	0.016157	3.074581
H	5.794055	4.867458	-0.261304	H	-5.851758	-0.014945	4.152702
C	1.828171	-4.840410	-0.675582	C	-5.576844	1.318830	2.324935
C	4.611074	-4.900339	-0.443982	H	-4.718794	1.893804	2.691430
C	2.538038	-3.706825	-0.263567	H	-6.466034	1.950429	2.437198

cis-par-**CP3b**

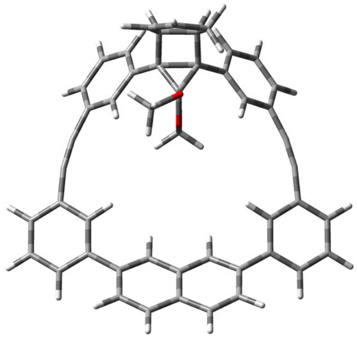

#p opt freq rwb97xd/6-31g(d)

Charge = 0, Multiplicity = 1

Number of Imaginary Frequencies = 0

Sum of electronic and zero-point Energies = -1998.505783 Hartree

Sum of electronic and thermal Energies = -1998.466957 Hartree

Sum of electronic and enthalpy Energies = -1998.466013 Hartree

Sum of electronic and thermal Free Energies = -1998.576206 Hartree

	Cartesian Coordinates				Cartesian Coordinates		
**Atom**	***X***	***Y***	***Z***	**Atom**	***X***	***Y***	***Z***
C	-3.842946	-0.757829	0.468620	C	2.386179	-5.999491	-1.042985
C	-2.780593	0.058137	1.164253	C	3.762658	-6.029217	-0.842309
C	-3.836740	0.863815	0.484624	C	3.760040	-3.702891	-0.162883
C	-5.182955	0.831781	1.231956	H	1.811444	-2.792305	-0.113590
C	-5.207462	-0.711497	1.197434	H	1.858171	-6.880507	-1.392637
C	-3.531839	1.931611	-0.502398	H	4.312499	-6.944569	-1.039279
C	-2.911865	3.972827	-2.329434	H	5.527849	-4.930118	-0.299653
C	-4.351260	2.169147	-1.609905	C	4.442579	-2.452403	0.256857
C	-2.416322	2.743655	-0.308853	H	3.176746	-1.203824	-0.931570
C	-2.073684	3.738092	-1.233395	C	3.988263	-1.239735	-0.209501
C	-4.050853	3.193343	-2.503402	C	6.112790	-1.293009	1.591106
H	-5.222869	1.541420	-1.777126	C	4.540947	-0.016341	0.235866
H	-1.767130	2.565280	0.541843	C	5.538520	-2.462446	1.163513
H	-4.696182	3.369659	-3.358677	C	5.621010	-0.034221	1.155790
H	-2.653485	4.742541	-3.049310	C	4.008100	1.224302	-0.185282
C	-3.553096	-1.807408	-0.541697	H	5.901869	-3.412685	1.544736
C	-2.975735	-3.825650	-2.412831	H	6.960182	1.206343	2.321075
C	-2.452722	-2.644901	-0.371316	H	6.939499	-1.319007	2.296412
C	-4.377834	-2.008134	-1.653397	C	4.482019	2.420015	0.304668
C	-4.099182	-3.020008	-2.567765	H	3.195819	1.215859	-0.907298
C	-2.130599	-3.627775	-1.315164	C	5.578051	2.394257	1.210913
H	-1.792543	-2.495059	0.476287	H	5.956634	3.330874	1.610552
H	-5.237052	-1.360404	-1.807423	C	6.133271	1.207583	1.615536
H	-4.749774	-3.166017	-3.424774	O	-1.475237	0.043356	0.649419

Atom	Cartesian Coordinates			Atom	Cartesian Coordinates		
	X	*Y*	*Z*		*X*	*Y*	*Z*
H	-2.734460	-4.586077	-3.148282	O	-2.652109	0.127035	2.528724
C	-0.868619	-4.290246	-1.172251	C	-1.130287	0.041960	-0.726649
C	0.273016	-4.659168	-1.005235	H	-1.989475	0.060684	-1.398798
C	-0.799956	4.374723	-1.077329	H	-0.518634	0.927893	-0.926866
C	0.348183	4.720846	-0.906352	H	-0.546328	-0.859795	-0.938852
C	1.759481	4.850622	-0.694866	C	-2.133530	-1.050811	3.113461
C	4.528258	4.873992	-0.321963	H	-2.716738	-1.928861	2.804017
C	2.484373	6.024271	-0.925856	H	-2.226204	-0.927226	4.193848
C	2.436112	3.704537	-0.260757	H	-1.081779	-1.195460	2.844915
C	3.820028	3.689492	-0.090256	H	-5.958328	1.283861	0.608021
C	3.861682	6.026794	-0.728592	H	-5.979196	-1.132213	0.542435
H	1.970846	6.920357	-1.258346	C	-5.381504	-1.125637	2.631233
H	1.855657	2.812488	-0.049222	H	-5.446860	-2.159953	2.954276
H	4.426745	6.935910	-0.910875	C	-5.464666	-0.062756	3.432454
H	5.608896	4.887741	-0.210440	H	-5.599650	-0.113832	4.509519
C	1.681375	-4.817473	-0.793364	C	-5.310936	1.259180	2.710779
C	4.447953	-4.895489	-0.413640	H	-4.426516	1.798106	3.066045
C	2.376622	-3.691121	-0.337638	H	-6.178831	1.909831	2.873115

cis-*twi*-**CP3b**

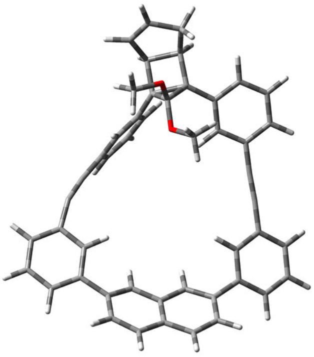

```
#p opt freq rwb97xd/6-31g(d)
```

Charge = 0, Multiplicity = 1

Number of Imaginary Frequencies = 0

Sum of electronic and zero-point Energies = -1998.516986 Hartree

Sum of electronic and thermal Energies = -1998.477875 Hartree

Sum of electronic and enthalpy Energies = -1998.476931 Hartree

Sum of electronic and thermal Free Energies = -1998.588301 Hartree

	Cartesian Coordinates				Cartesian Coordinates		
**Atom**	***X***	***Y***	***Z***	**Atom**	***X***	***Y***	***Z***
C	3.794914	-0.578693	-1.191431	H	-1.323683	6.555194	1.226791
C	4.025571	-1.072826	0.199176	H	-3.745187	7.059331	1.077544
C	4.525920	0.363339	-0.263864	H	-5.358462	5.298854	0.436542
C	4.066988	1.745400	0.040505	C	-4.758089	2.662515	-0.180105
C	3.332025	4.429273	0.453261	H	-3.746723	1.273300	1.088046
C	2.737174	2.080486	0.319114	C	-4.515221	1.410932	0.331945
C	5.018238	2.772413	-0.003778	C	-6.502148	1.723991	-1.594742
C	4.656619	4.097463	0.211837	C	-5.186146	0.269009	-0.158217
C	2.360111	3.419697	0.494616	C	-5.803657	2.812483	-1.133499
H	1.982357	1.304039	0.361471	C	-6.182109	0.410449	-1.156655
H	6.054091	2.533820	-0.226701	C	-4.778775	-1.020265	0.241413
H	5.412135	4.876434	0.172795	H	-6.016024	3.800012	-1.534096
H	3.033622	5.462738	0.595218	H	-7.519366	-0.683816	-2.464810
C	2.954635	-1.486942	1.139603	H	-7.276067	1.854975	-2.346763
C	1.092503	-2.422367	3.009456	C	-5.245086	-2.155160	-0.381281
C	1.957167	-2.380549	0.749875	H	-4.028534	-1.096789	1.022741
C	2.997238	-1.051392	2.466831	C	-6.276966	-2.014127	-1.349602
C	2.076370	-1.522099	3.396567	H	-6.669368	-2.900248	-1.841146
C	1.017552	-2.849469	1.675812	C	-6.745040	-0.773085	-1.707115
H	1.893786	-2.699428	-0.285068	H	2.119738	-1.178618	4.425614
H	3.758832	-0.335848	2.766068	H	-4.978865	-6.781927	0.402102
H	0.361146	-2.784646	3.724563	C	5.504241	-1.459654	0.460396
C	0.981942	3.783814	0.641010	H	5.638217	-1.631565	1.533754
C	-0.171782	4.151206	0.679564	C	5.983336	-0.070865	-0.019854

	Cartesian Coordinates				Cartesian Coordinates		
**Atom**	***X***	***Y***	***Z***	**Atom**	***X***	***Y***	***Z***
C	-0.085877	-3.665275	1.259357	H	6.416090	0.518219	0.792359
C	-1.105109	-4.242279	0.947104	O	4.427088	-1.071292	-2.313167
C	-2.467575	-4.511097	0.576817	O	2.459269	-0.279599	-1.413937
C	-5.201349	-4.686318	-0.005435	C	3.965907	-2.355998	-2.687085
C	-3.198425	-3.389362	0.177935	H	4.595533	-2.684312	-3.515559
C	-3.115888	-5.746972	0.650217	H	4.068611	-3.063885	-1.854592
C	-4.473185	-5.822754	0.343043	H	2.917502	-2.325263	-3.007336
C	-4.567886	-3.441620	-0.078071	C	2.246521	0.604201	-2.505924
H	-2.666741	-2.450453	0.078498	H	2.421476	0.098606	-3.461551
H	-2.567794	-6.633424	0.952146	H	1.204193	0.920392	-2.441098
H	-6.268864	-4.765469	-0.192336	H	2.896528	1.483453	-2.441055
C	-1.562491	4.492081	0.649144	C	6.991160	-0.305997	-1.166313
C	-4.297157	5.070431	0.482829	H	7.966996	0.147894	-0.952737
C	-2.483919	3.508220	0.278103	H	6.641934	0.104042	-2.119963
C	-2.028187	5.781121	0.940578	C	7.085320	-1.815896	-1.230423
C	-3.387737	6.059362	0.850811	H	7.746241	-2.318537	-1.931352
C	-3.852739	3.775642	0.201539	C	6.268443	-2.441335	-0.381767
H	-2.113361	2.521676	0.020082	H	6.175041	-3.518440	-0.283117

trans-**TS3b**

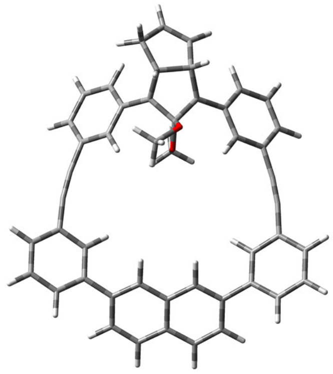

`#p opt=(ts,calcfc) freq uwb97xd/6-31g(d) guess=(mix,always)`

Charge = 0, Multiplicity = 1

Number of Imaginary Frequencies = 1, v_i = -242.93

Sum of electronic and zero-point Energies = -1998.485460 Hartree

Sum of electronic and thermal Energies = -1998.446830 Hartree

Sum of electronic and enthalpy Energies = -1998.445886 Hartree

Sum of electronic and thermal Free Energies = -1998.555548 Hartree

	Cartesian Coordinates				Cartesian Coordinates		
**Atom**	***X***	***Y***	***Z***	**Atom**	***X***	***Y***	***Z***
C	-5.286661	-0.558741	1.353439	C	2.774463	3.655591	-0.049777
H	-5.087947	-0.653078	2.428417	C	4.140704	3.608629	0.228626
C	-4.018508	-0.894219	0.608410	C	4.045462	5.961672	0.801105
C	-3.052695	0.211775	0.920574	H	2.114781	6.913222	0.659362
C	-4.029722	1.205593	0.380587	H	2.265231	2.770335	-0.416031
C	-5.413111	0.934118	0.930946	H	4.548595	6.862683	1.138809
H	-5.640626	1.633457	1.752422	H	5.830399	4.769894	0.896469
C	-3.698699	-2.102433	-0.144573	C	4.735363	-2.543237	-0.022384
C	-3.013216	-4.424518	-1.595464	H	3.181942	-1.288283	0.744796
C	-2.464740	-2.739625	0.048605	C	4.179482	-1.330065	0.315324
C	-4.572070	-2.642060	-1.106517	C	6.763156	-1.399938	-0.725713
C	-4.234953	-3.794029	-1.807349	C	4.865987	-0.111591	0.100259
C	-2.109947	-3.884446	-0.672115	C	6.061330	-2.562067	-0.537291
H	-1.751453	-2.326307	0.750869	C	6.180834	-0.139073	-0.432995
H	-5.497174	-2.123659	-1.336289	C	4.247082	1.134868	0.356600
H	-2.742371	-5.312373	-2.157275	H	7.840331	1.086841	-1.090246
C	-3.608794	2.417154	-0.312404	H	7.769452	-1.431902	-1.135486
C	-2.722989	4.798849	-1.548814	C	4.870099	2.325986	0.060368
C	-2.334923	2.948134	-0.041574	H	3.247489	1.134759	0.783378
C	-4.426919	3.116577	-1.217665	C	6.196386	2.288719	-0.452794
C	-3.990145	4.292888	-1.815379	C	6.833072	1.096736	-0.681724
C	-1.881601	4.115494	-0.660999	H	-4.923460	-4.192498	-2.546396
H	-1.683746	2.437149	0.657130	H	4.168483	-7.092132	0.893115
H	-5.400795	2.723647	-1.480430	C	-6.590163	0.878504	-0.025530

	Cartesian Coordinates				Cartesian Coordinates		
Atom	*X*	*Y*	*Z*	Atom	*X*	*Y*	*Z*
H	-4.640554	4.813102	-2.512118	H	-6.922906	1.736000	-0.599372
H	-2.374622	5.706540	-2.030604	C	-7.218951	-0.294788	-0.008879
C	-0.793965	-4.416861	-0.477673	H	-8.107816	-0.528836	-0.587503
C	0.357467	-4.727997	-0.269885	C	-6.603473	-1.252898	0.976324
C	-0.537230	4.538573	-0.401943	H	-7.248329	-1.348949	1.859931
C	0.626611	4.767130	-0.159655	H	-6.463063	-2.267076	0.590969
C	1.761192	-4.885838	-0.028633	H	6.695641	3.221853	-0.697958
C	4.503159	-5.011604	0.482862	H	6.508155	-3.512157	-0.816198
C	2.567263	-3.749286	-0.157276	O	-2.771984	0.448299	2.277888
C	2.346081	-6.100282	0.346038	O	-1.801251	0.127981	0.283236
C	3.713481	-6.152506	0.594695	C	-1.719541	0.064075	-1.131087
C	3.936640	-3.788624	0.106369	H	-2.702310	-0.026307	-1.602073
H	2.105423	-2.824349	-0.486418	H	-1.119388	-0.809355	-1.403197
H	1.730285	-6.988155	0.444697	H	-1.235634	0.973548	-1.502284
H	5.563567	-5.067405	0.712297	C	-2.126181	-0.618468	2.941011
C	2.032357	4.831159	0.110056	H	-2.075798	-0.343315	3.996282
C	4.771217	4.782832	0.655082	H	-1.111847	-0.776498	2.557923
C	2.680687	5.995808	0.535505	H	-2.696308	-1.554660	2.843868

trans-*par*-**CP3b**

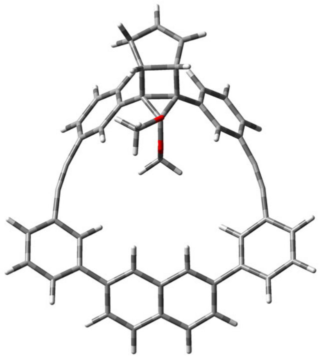

```
#p opt freq rwb97xd/6-31g(d)
```

Charge = 0, Multiplicity = 1

Number of Imaginary Frequencies = 0

Sum of electronic and zero-point Energies = -1998.514302 Hartree

Sum of electronic and thermal Energies = -1998.475645 Hartree

Sum of electronic and enthalpy Energies = -1998.474701 Hartree

Sum of electronic and thermal Free Energies = -1998.584265 Hartree

	Cartesian Coordinates				Cartesian Coordinates		
**Atom**	***X***	***Y***	***Z***	**Atom**	***X***	***Y***	***Z***
C	-5.188615	-0.666635	1.483241	C	2.603781	3.666056	-0.157179
H	-5.069002	-1.100526	2.478265	C	3.949073	3.647206	0.209772
C	-3.884253	-0.682129	0.659564	C	3.772249	6.006730	0.734034
C	-2.856018	0.117639	1.432734	H	1.835592	6.918040	0.461250
C	-3.866059	0.904649	0.657815	H	2.135540	2.766266	-0.541876
C	-5.205320	0.893479	1.415795	H	4.236035	6.922733	1.087365
H	-5.199197	1.452180	2.356912	H	5.570020	4.853370	0.964530
C	-3.536050	-1.704902	-0.358125	C	4.604707	-2.498803	0.046530
C	-2.774397	-3.617017	-2.271353	H	2.996492	-1.249335	0.702820
C	-2.500548	-2.600328	-0.098137	C	4.022106	-1.287584	0.344896
C	-4.200487	-1.788964	-1.586107	C	6.673018	-1.346779	-0.509868
C	-3.835722	-2.753613	-2.521753	C	4.715951	-0.066118	0.175388
C	-2.081962	-3.528811	-1.058189	C	5.964723	-2.512168	-0.370362
H	-1.951400	-2.526184	0.834923	C	6.064816	-0.087783	-0.264754
H	-4.995189	-1.082620	-1.810647	C	4.072151	1.177457	0.378506
H	-2.456451	-4.334467	-3.020750	H	7.757819	1.146604	-0.811893
C	-3.475983	1.940592	-0.334593	H	7.706295	-1.374852	-0.846218
C	-2.622274	3.881631	-2.176972	C	4.703850	2.371500	0.112628
C	-2.398791	2.775365	-0.041421	H	3.045695	1.171983	0.736065
C	-4.138817	2.103549	-1.555099	C	6.063523	2.340453	-0.304546
C	-3.726307	3.082650	-2.454929	C	6.724033	1.151513	-0.475961
C	-1.936054	3.716592	-0.968519	H	-4.362637	-2.811796	-3.469362
H	-1.862316	2.646667	0.892852	H	3.948411	-7.052753	0.894345
H	-4.976518	1.457601	-1.798173	C	-6.399702	1.192129	0.551846

Atom	Cartesian Coordinates			Atom	Cartesian Coordinates		
	X	*Y*	*Z*		*X*	*Y*	*Z*
H	-4.251859	3.203158	-3.397494	H	-6.659862	2.202581	0.252068
H	-2.269531	4.609269	-2.900641	C	-7.067650	0.093418	0.202264
C	-0.859212	-4.235203	-0.808520	H	-7.962822	0.081785	-0.413028
C	0.248680	-4.635684	-0.526880	C	-6.472828	-1.161290	0.792446
C	-0.682995	4.358204	-0.694594	H	-7.161006	-1.614941	1.517699
C	0.440539	4.703054	-0.401730	H	-6.273711	-1.932421	0.038949
C	1.630179	-4.827702	-0.191615	H	6.571967	3.275262	-0.523145
C	4.326974	-4.973190	0.520603	H	6.434866	-3.460545	-0.614753
C	2.453131	-3.697564	-0.257079	O	-2.908919	0.183040	2.807405
C	2.176868	-6.046959	0.220023	O	-1.507989	0.099993	1.073619
C	3.522883	-6.108763	0.567565	C	-1.024915	0.066042	-0.260713
C	3.797972	-3.744556	0.109132	H	-1.813255	0.088125	-1.014554
H	2.023092	-2.768917	-0.617051	H	-0.439359	-0.849188	-0.397669
H	1.550193	-6.931204	0.271136	H	-0.381465	0.939263	-0.410121
H	5.366879	-5.037915	0.828324	C	-2.399800	-0.973021	3.445736
C	1.828270	4.827075	-0.060494	H	-2.607168	-0.856670	4.510982
C	4.528636	4.840650	0.655136	H	-1.321077	-1.070494	3.288267
C	2.425185	6.010431	0.384939	H	-2.901047	-1.879321	3.075826

trans-*twi*-**CP3b**

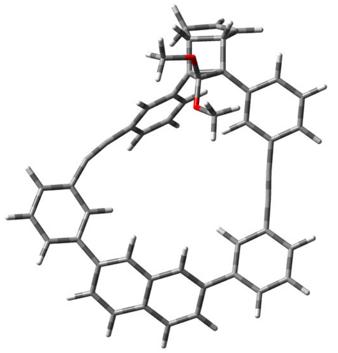

```
#p opt freq rwb97xd/6-31g(d)
```

Charge = 0, Multiplicity = 1

Number of Imaginary Frequencies = 0

Sum of electronic and zero-point Energies = -1998.525597 Hartree

Sum of electronic and thermal Energies = -1998.486509 Hartree

Sum of electronic and enthalpy Energies = -1998.485565 Hartree

Sum of electronic and thermal Free Energies = -1998.596672 Hartree

	Cartesian Coordinates				Cartesian Coordinates		
Atom	*X*	*Y*	*Z*	Atom	*X*	*Y*	*Z*
C	-4.005243	-0.807598	1.426234	H	2.979615	-2.389689	-0.440519
C	-4.674616	0.103515	0.421732	H	2.563870	-6.533999	0.530592
C	-4.062742	-1.279126	0.008386	H	6.328414	-4.543298	1.150818
C	-2.886328	-1.602145	-0.834003	C	6.752332	-1.831539	-0.241980
C	-0.769230	-2.306273	-2.520841	H	8.235258	-0.472063	-0.957250
C	-1.917372	-2.507067	-0.400851	C	7.251788	-0.579448	-0.506791
C	-2.776391	-1.040423	-2.109230	C	4.742233	-0.868610	0.655469
C	-1.727970	-1.398869	-2.950308	C	6.469744	0.584929	-0.277496
C	-0.849279	-2.859446	-1.233680	C	5.451038	-1.994843	0.306058
H	-1.973291	-2.918429	0.601795	C	5.202643	0.426246	0.337595
H	-3.523172	-0.319489	-2.431860	C	6.843759	1.889692	-0.698140
H	-1.651659	-0.959894	-3.940359	H	3.762319	-0.966763	1.114379
H	0.062306	-2.578166	-3.162918	C	5.972252	2.944380	-0.588977
C	-4.250570	1.512096	0.211227	H	7.815134	2.035094	-1.163897
C	-3.564694	4.229533	0.001866	C	4.682460	2.778175	-0.009443
C	-5.223766	2.513609	0.314206	C	4.352895	1.543276	0.496299
C	-2.921980	1.890197	-0.008832	H	3.394641	1.390565	0.984826
C	-2.569923	3.245573	-0.088074	O	-4.780831	-1.378068	2.420362
C	-4.886285	3.856980	0.197496	O	-2.736140	-0.452556	1.837787
H	-6.255817	2.237966	0.509546	C	-4.270187	-2.624218	2.860448
H	-2.148550	1.133609	-0.076597	H	-4.986327	-3.018909	3.583064
H	-5.657492	4.617140	0.277844	H	-3.290523	-2.508407	3.338477
H	-3.285972	5.275916	-0.066259	H	-4.171938	-3.327646	2.021878
C	-1.199905	3.653942	-0.186834	C	-2.721889	0.444316	2.940469

Atom	Cartesian Coordinates			Atom	Cartesian Coordinates		
	X	*Y*	*Z*		*X*	*Y*	*Z*
C	-0.060157	4.064560	-0.185959	H	-3.038702	-0.059108	3.860429
C	0.234708	-3.669979	-0.760966	H	-3.372337	1.307036	2.761090
C	1.249395	-4.224862	-0.397236	H	-1.690155	0.784049	3.041929
C	1.307184	4.483389	-0.105489	C	-6.078642	-0.427657	0.089802
C	3.993808	5.208303	0.177665	C	-5.483042	-1.835902	-0.236346
C	1.657918	5.829025	0.069801	H	3.265807	7.222019	0.343118
C	2.319314	3.520603	-0.159208	H	4.942743	-6.585915	1.215864
C	3.665410	3.860433	0.002762	H	7.333135	-2.712570	-0.501390
C	2.996342	6.179688	0.201658	H	6.253451	3.915213	-0.987501
H	0.881250	6.585815	0.107602	H	-5.753431	-2.638038	0.453394
H	2.038209	2.488696	-0.339708	H	-6.814842	-0.355272	0.897532
H	5.031053	5.497913	0.321018	C	-6.602845	0.033756	-1.246426
C	2.608449	-4.438370	0.018714	H	-7.025986	1.020803	-1.402098
C	5.295110	-4.506424	0.816552	C	-6.474671	-0.889727	-2.197260
C	3.415505	-3.299406	-0.043964	H	-6.787989	-0.761958	-3.229244
C	3.167706	-5.634030	0.476889	C	-5.863617	-2.169764	-1.688348
C	4.507911	-5.656430	0.860568	H	-6.586977	-2.994524	-1.729191
C	4.745151	-3.300012	0.373285	H	-4.998546	-2.486974	-2.282605

Optimized Macrocyclic Skeleton in **3a**

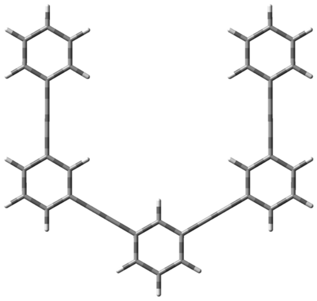

```
#p opt rwb97xd/6-31g(d)
```

Charge = 0, Multiplicity = 1

Number of Imaginary Frequencies = 0

Electronic energies = -1460.564270 Hartree

	Cartesian Coordinates				Cartesian Coordinates		
**Atom**	***X***	***Y***	***Z***	**Atom**	***X***	***Y***	***Z***
C	4.714457	5.698155	-0.004151	H	-8.091332	-0.619647	-0.005084
C	7.135470	4.319345	-0.002580	H	-3.797671	-0.635048	0.000371
C	4.716108	4.309049	-0.002977	H	-8.097051	-3.100075	-0.007735
C	5.917015	6.401125	-0.004539	H	-5.953593	-4.348798	-0.006326
C	7.125506	5.708416	-0.003750	C	5.944588	-0.465746	0.001274
C	5.928823	3.605191	-0.002186	C	5.957138	-3.263894	0.003711
H	3.782259	3.756147	-0.002671	C	4.738534	-1.174374	0.000869
H	5.912425	7.486921	-0.005458	C	7.156782	-1.170988	0.002912
H	8.065008	6.252773	-0.004049	C	7.155195	-2.560353	0.004112
H	8.074079	3.774526	-0.001966	C	4.736830	-2.573094	0.002085
C	-4.714351	5.698201	0.005401	H	3.797664	-0.635051	-0.000393
C	-7.135385	4.319431	0.002297	H	8.091329	-0.619783	0.003228
C	-5.916897	6.401189	0.005428	H	8.096971	-3.100210	0.005378
C	-4.716023	4.309096	0.003833	H	5.953477	-4.348867	0.004656
C	-5.928747	3.605258	0.002263	C	3.498981	-3.289909	0.001682
C	-7.125400	5.708502	0.003874	C	2.451837	-3.896484	0.001375
H	-5.912288	7.486986	0.006665	C	-3.499069	-3.289915	-0.002004
H	-3.782183	3.756179	0.003809	C	-2.451936	-3.896510	-0.001180
H	-8.064892	6.252875	0.003894	C	1.214289	-4.613674	0.001081
H	-8.074001	3.774626	0.001090	C	-1.206203	-6.015956	0.000597
C	5.934684	2.174820	-0.000992	C	-0.000049	-3.919519	0.000055
C	5.938393	0.964478	0.000027	C	1.206125	-6.015943	0.001856
C	-5.934633	2.174887	0.000670	C	-0.000036	-6.705521	0.001606
C	-5.938349	0.964546	-0.000660	C	-1.214381	-4.613686	-0.000188
C	-5.944588	-0.465678	-0.002200	H	-0.000054	-2.835050	-0.000536

Atom	Cartesian Coordinates X	Y	Z	Atom	Cartesian Coordinates X	Y	Z
C	-5.957223	-3.263825	-0.005183	H	2.149118	-6.552558	0.002658
C	-7.156803	-1.170882	-0.004485	H	-0.000030	-7.791030	0.002218
C	-4.738557	-1.174342	-0.001417	H	-2.149190	-6.552580	0.000423
C	-4.736894	-2.573061	-0.002891	H	3.770389	6.234562	-0.004766
C	-7.155258	-2.560248	-0.005963	H	-3.770275	6.234592	0.006614

Strained Macrocyclic Skeleton in **AZ3a**

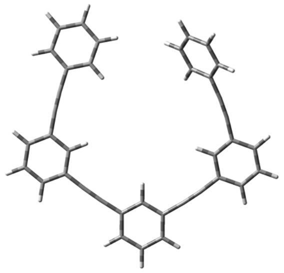

```
#p opt rwb97xd/6-31g(d)
```

Charge = 0, Multiplicity = 1

Number of Imaginary Frequencies = 0

Electronic energies = -1460.561218 Hartree

	Cartesian Coordinates				Cartesian Coordinates		
**Atom**	***X***	***Y***	***Z***	**Atom**	***X***	***Y***	***Z***
C	4.569615	-0.127060	-0.165453	H	6.945906	-2.532900	-0.499356
H	3.495227	-0.014964	-0.070020	C	-2.424880	-4.058263	0.246920
C	-3.723262	-3.466166	0.350977	C	-3.905645	3.139975	-0.522184
C	-5.159302	2.545657	-0.343376	H	-3.013175	2.528621	-0.462156
C	-3.787527	4.504410	-0.782943	C	7.323585	-0.413088	-0.407265
C	5.123411	-1.405688	-0.283471	H	8.399122	-0.524664	-0.503314
C	2.411028	-4.429621	-0.160223	C	-4.947480	5.274983	-0.877131
C	3.435928	-3.433608	-0.223570	H	-4.883044	6.335337	-1.102730
C	3.303471	4.464895	0.307954	C	-3.858553	-2.084729	0.179214
C	1.078215	-4.020752	-0.044994	H	-2.982831	-1.481305	-0.033160
H	0.842869	-2.962655	-0.012211	C	1.539867	6.578272	0.764511
C	2.047368	4.487968	-0.310774	H	0.845997	7.384965	0.977467
H	1.756169	3.670936	-0.960971	C	-6.241273	-2.259292	0.544860
C	-4.860579	-4.239774	0.621175	H	-7.215171	-1.786589	0.617754
H	-4.757072	-5.311564	0.754504	C	1.682665	-6.734235	-0.129712
C	5.385029	1.009055	-0.161540	H	1.918286	-7.793359	-0.163402
C	4.141238	3.318598	0.123847	C	-6.313563	3.336250	-0.424050
C	-5.228164	1.137407	-0.100662	H	-7.287803	2.878440	-0.286947
C	0.046264	-4.961571	0.030003	C	-6.107162	-3.632269	0.716292
C	-5.113120	-1.472986	0.273366	H	-6.984689	-4.236060	0.925723
C	-1.304495	-4.505136	0.148394	C	2.797928	6.575025	1.359845
C	4.247288	-2.536460	-0.262751	H	3.086055	7.393526	2.012545
C	-5.212790	-0.058886	0.082327	C	-6.199791	4.693575	-0.691840
C	6.772408	0.857695	-0.283826	H	-7.093706	5.305128	-0.767552
H	7.407936	1.737092	-0.282094	C	3.681242	5.525228	1.140847

Atom	Cartesian Coordinates			Atom	Cartesian Coordinates		
	X	*Y*	*Z*		*X*	*Y*	*Z*
C	4.765403	2.291754	-0.019846	H	4.654515	5.510339	1.620423
C	1.155614	5.532633	-0.079334	C	2.708191	-5.798531	-0.203205
C	0.358533	-6.327428	-0.012952	H	3.741303	-6.117696	-0.292832
H	-0.440551	-7.059101	0.044586	H	-2.809306	4.952807	-0.922878
C	6.512698	-1.542445	-0.406707	H	0.177540	5.531319	-0.549708

Strained Macrocyclic Skeleton in S-**DR3a**

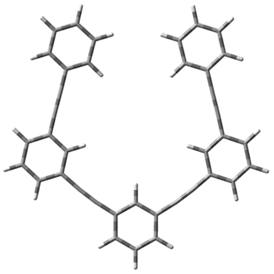

`#p opt rwb97xd/6-31g(d)`

Charge = 0, Multiplicity = 1

Number of Imaginary Frequencies = 0

Electronic energies = -1460.559894 Hartree

	Cartesian Coordinates				Cartesian Coordinates		
**Atom**	***X***	***Y***	***Z***	**Atom**	***X***	***Y***	***Z***
C	2.578984	5.431406	-0.135714	H	-7.329922	0.069285	0.004273
C	5.278019	4.611944	0.027904	H	-3.145412	-0.887768	-0.009122
C	2.920069	4.057512	-0.105358	H	-7.878141	-2.348415	-0.010693
C	3.636233	6.373735	-0.077480	H	-6.062975	-4.039204	-0.023871
C	4.957653	5.964957	0.003564	C	5.197291	-0.307625	0.030871
C	4.252011	3.652131	-0.026615	C	5.789391	-3.045106	0.065524
H	2.135205	3.312774	-0.150272	C	4.166747	-1.252133	0.016087
H	3.412772	7.435669	-0.086542	C	6.528924	-0.746333	0.063399
H	5.748988	6.707080	0.049904	C	6.814486	-2.106335	0.080316
H	6.311114	4.286424	0.089142	C	4.454157	-2.621411	0.033412
C	-2.517485	5.460436	-0.083115	H	3.134647	-0.920052	-0.008769
C	-5.219613	4.667953	0.142867	H	7.329776	-0.014330	0.074994
C	-3.559757	6.412696	0.039623	H	7.847709	-2.438504	0.105398
C	-2.874422	4.090794	-0.084413	H	6.012102	-4.106920	0.079062
C	-4.208290	3.698493	0.025365	C	3.369795	-3.553646	0.019087
C	-4.883105	6.017194	0.150947	C	2.402165	-4.280162	0.007007
H	-3.321548	7.471367	0.055320	C	-3.413134	-3.518070	-0.022243
H	-2.101064	3.338623	-0.178224	C	-2.453518	-4.255245	-0.023557
H	-5.663020	6.766629	0.246693	C	1.186076	-5.032525	-0.006744
H	-6.254249	4.352714	0.227563	C	-1.245330	-6.421460	-0.032696
C	4.582873	2.259909	-0.004796	C	-0.026107	-4.335893	-0.009742
C	4.880801	1.087061	0.012233	C	1.172099	-6.433684	-0.016944
C	-4.555583	2.310110	0.015499	C	-0.040053	-7.114919	-0.029917
C	-4.867204	1.140716	0.007352	C	-1.245239	-5.020239	-0.022454
C	-5.200763	-0.250121	-0.001799	H	-0.020671	-3.251522	-0.001639

Atom	Cartesian Coordinates			Atom	Cartesian Coordinates		
	X	*Y*	*Z*		*X*	*Y*	*Z*
C	-5.827026	-2.980183	-0.017965	H	2.110886	-6.977659	-0.014643
C	-6.538064	-0.672501	-0.001985	H	-0.045496	-8.200443	-0.037845
C	-4.181804	-1.207163	-0.009817	H	-2.189517	-6.955919	-0.042595
C	-4.486316	-2.572867	-0.017379	H	1.543896	5.749128	-0.198100
C	-6.840631	-2.028910	-0.010355	H	-1.481051	5.767952	-0.169840

Strained Macrocyclic Skeleton in *cis*-*par*-**CP3a**

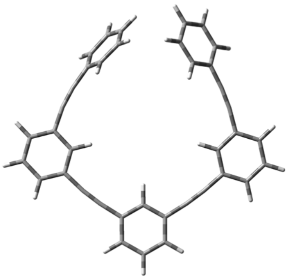

#p opt rwb97xd/6-31g(d)

Charge = 0, Multiplicity = 1

Number of Imaginary Frequencies = 0

Electronic energies = -1460.556960 Hartree

	Cartesian Coordinates				Cartesian Coordinates		
**Atom**	***X***	***Y***	***Z***	**Atom**	***X***	***Y***	***Z***
C	-2.740683	-4.281408	1.018569	H	-4.634207	5.432239	-0.361109
C	-1.386929	-5.151102	-1.285016	H	-1.268606	2.807556	0.084438
C	-1.356034	-4.420195	1.018175	H	-3.093125	7.367370	-0.522505
C	-3.445227	-4.622783	-0.139683	H	-0.641312	7.029652	-0.383739
C	-2.774694	-5.071357	-1.272949	C	3.300799	-3.998642	-0.086856
C	-0.665410	-4.809190	-0.135822	C	5.882874	-2.912197	-0.022940
H	-0.791175	-4.196197	1.915328	C	3.487028	-2.613839	-0.033470
H	-4.525603	-4.515743	-0.157657	C	4.420816	-4.838464	-0.108414
H	-3.336407	-5.330662	-2.165169	C	5.699086	-4.289673	-0.075693
H	-0.856228	-5.452054	-2.182308	C	4.770687	-2.060337	-0.002516
C	-5.040048	-1.675237	1.301778	H	2.622164	-1.959576	-0.020437
C	-6.090379	0.041685	-0.673077	H	4.283155	-5.913897	-0.149734
C	-6.037487	-2.109853	0.420153	H	6.564185	-4.945264	-0.092076
C	-4.592132	-0.359431	1.185153	H	6.881906	-2.489577	0.000881
C	-5.089463	0.494349	0.193406	C	4.885669	-0.634716	0.042814
C	-6.562126	-1.259057	-0.547632	C	4.849128	0.574828	0.073416
H	-6.410805	-3.128485	0.486118	C	0.615305	4.649088	-0.067427
H	-3.815638	0.003904	1.844280	C	1.786034	4.347689	-0.006744
H	-7.335954	-1.619268	-1.218847	C	4.672949	1.994577	0.098081
H	-6.478120	0.702063	-1.441845	C	4.227072	4.759074	0.134993
C	0.764937	-4.716930	-0.142208	C	3.370861	2.502562	0.039443
C	1.953062	-4.486055	-0.115671	C	5.752806	2.883899	0.176193
C	-4.499875	1.793288	0.059609	C	5.521678	4.255038	0.193961
C	-3.896848	2.839345	-0.026165	C	3.137338	3.881296	0.057017
C	-3.051888	3.991380	-0.125171	H	2.531750	1.818424	-0.023337

	Cartesian Coordinates				Cartesian Coordinates		
Atom	*X*	*Y*	*Z*	Atom	*X*	*Y*	*Z*
C	-1.316462	6.183427	-0.310480	H	6.764333	2.494109	0.221760
C	-3.561034	5.284840	-0.298942	H	6.361719	4.939930	0.254233
C	-1.667444	3.807597	-0.045885	H	4.050765	5.829550	0.148746
C	-0.792720	4.894834	-0.139464	H	-4.650108	-2.324599	2.075298
C	-2.692324	6.367385	-0.388718	H	-3.256223	-3.963762	1.916758

Strained Macrocyclic Skeleton in *cis*-*twi*-**CP3a**

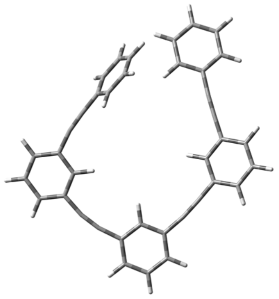

```
#p opt rwb97xd/6-31g(d)
```

Charge = 0, Multiplicity = 1

Number of Imaginary Frequencies = 0

Electronic energies = -1460.558700 Hartree

	Cartesian Coordinates				Cartesian Coordinates		
**Atom**	***X***	***Y***	***Z***	**Atom**	***X***	***Y***	***Z***
C	-3.417843	-3.790053	0.127155	H	2.764136	-1.862377	0.095409
C	-1.465582	-3.356501	2.087198	H	3.303704	-6.105491	-0.217989
C	-2.076039	-4.044889	-0.150756	H	5.705212	-5.750305	-0.711474
C	-3.771161	-3.308547	1.390830	H	6.644992	-3.459364	-0.806599
C	-2.801271	-3.097219	2.364966	C	-2.489011	3.466000	0.264127
C	-1.092701	-3.827783	0.820661	C	-0.700263	5.557862	0.788152
H	-1.786120	-4.391725	-1.136782	C	-1.112323	3.268873	0.118850
H	-4.814044	-3.086094	1.601612	C	-2.960906	4.720650	0.678889
H	-3.086507	-2.718760	3.341706	C	-2.068075	5.753096	0.937787
H	-0.700506	-3.184968	2.837323	C	-0.212529	4.310310	0.376787
C	-5.869836	-1.679990	-1.241151	H	-0.738220	2.302738	-0.201961
C	-6.531383	1.025279	-0.873490	H	-4.029067	4.873908	0.792183
C	-7.175650	-1.221873	-1.451095	H	-2.442223	6.720556	1.257950
C	-4.895275	-0.764556	-0.830311	H	-0.001490	6.363420	0.988168
C	-5.216334	0.588024	-0.658941	C	5.199032	-1.144213	-0.401484
C	-7.503256	0.116726	-1.266075	C	5.334136	0.058899	-0.389705
H	-7.941773	-1.921716	-1.773460	C	1.190573	4.079054	0.217996
H	-3.877383	-1.096007	-0.662588	C	2.366825	3.833140	0.073144
H	-8.521676	0.452672	-1.436437	C	3.713578	3.380028	-0.096900
H	-6.773528	2.073982	-0.735930	C	6.306613	2.364173	-0.431019
C	-4.216202	1.546603	-0.294830	C	3.933264	2.002727	-0.171896
C	-3.412874	2.412224	-0.027789	C	4.807683	4.250760	-0.190605
C	0.293053	-4.022526	0.512810	C	6.089556	3.736869	-0.357197
C	1.473622	-4.120914	0.264349	C	5.222168	1.484324	-0.336870
C	2.872182	-4.003381	-0.027282	H	3.091115	1.323490	-0.098855

Atom	Cartesian Coordinates			Atom	Cartesian Coordinates		
	X	***Y***	***Z***		***X***	***Y***	***Z***
C	5.593066	-3.611262	-0.588410	H	4.646622	5.322226	-0.133524
C	3.409544	-2.715648	-0.080948	H	6.932894	4.416551	-0.429902
C	3.711276	-5.100709	-0.257841	H	7.309404	1.970280	-0.559118
C	5.059771	-4.895537	-0.534498	H	-4.171606	-3.980288	-0.627520
C	4.763931	-2.506830	-0.361274	H	-5.622105	-2.721870	-1.402245

Strained Macrocyclic Skeleton in *trans*-*par*-**CP3a**

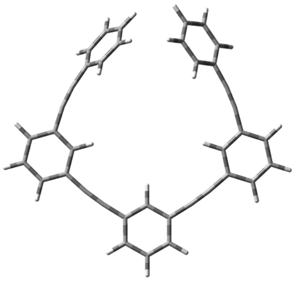

`#p opt rwb97xd/6-31g(d)`

Charge = 0, Multiplicity = 1

Number of Imaginary Frequencies = 0

Electronic energies = -1460.556101 Hartree

	Cartesian Coordinates				Cartesian Coordinates		
**Atom**	***X***	***Y***	***Z***	**Atom**	***X***	***Y***	***Z***
C	-4.726236	-2.236857	1.245862	H	5.033146	-5.333775	-0.198064
C	-5.981133	-0.707436	-0.760895	H	2.823934	-1.660032	-0.003721
C	-4.512822	-0.861623	1.154521	H	7.151993	-4.047007	-0.185253
C	-5.592228	-2.832605	0.321058	H	7.114883	-1.571541	-0.082432
C	-6.223038	-2.071896	-0.658928	C	-3.573196	3.647957	-0.122567
C	-5.107434	-0.095997	0.144736	C	-2.105180	6.030111	-0.272343
H	-3.833633	-0.376377	1.843245	C	-2.176454	3.625914	-0.046698
H	-5.779883	-3.899660	0.374529	C	-4.230052	4.875588	-0.274717
H	-6.894893	-2.553561	-1.363107	C	-3.493327	6.053383	-0.347072
H	-6.445161	-0.117603	-1.544745	C	-1.434384	4.808749	-0.122504
C	-2.137391	-4.597876	1.082653	H	-1.663833	2.677003	0.066993
C	-0.703008	-5.476884	-1.168060	H	-5.313221	4.898131	-0.334122
C	-2.803004	-5.123121	-0.028958	H	-4.008218	7.001880	-0.464344
C	-0.746508	-4.556291	1.064884	H	-1.533711	6.950518	-0.331924
C	-0.021319	-4.946677	-0.066785	C	-0.007229	4.725703	-0.053895
C	-2.088995	-5.577778	-1.133667	C	1.190046	4.557001	0.003556
H	-3.887930	-5.159498	-0.029623	C	4.878377	-0.023070	0.016503
H	-0.208013	-4.181034	1.927311	C	4.682264	1.170806	0.054390
H	-2.620136	-5.983868	-1.989100	C	2.586568	4.250629	0.062890
H	-0.146647	-5.780531	-2.048766	C	5.301550	3.568703	0.172946
C	-4.724226	1.279549	0.027024	C	2.981164	2.909054	0.034706
C	-4.272158	2.400420	-0.041810	C	3.565122	5.250403	0.146928
C	1.382358	-4.659662	-0.105399	C	4.910279	4.902686	0.201240
C	2.524080	-4.256931	-0.101467	C	4.334293	2.558349	0.088832
C	3.787433	-3.580108	-0.099445	H	2.228590	2.131097	-0.032916

Atom	Cartesian Coordinates			Atom	Cartesian Coordinates		
	X	*Y*	*Z*		*X*	*Y*	*Z*
C	6.186844	-2.133653	-0.086402	H	3.263340	6.292344	0.168984
C	5.015748	-4.249889	-0.152359	H	5.663612	5.681570	0.266231
C	3.773427	-2.182907	-0.040476	H	6.352053	3.300649	0.215040
C	4.964457	-1.450572	-0.034907	H	-4.256655	-2.815424	2.031275
C	6.202159	-3.522957	-0.144662	H	-2.680716	-4.274580	1.962239

Strained Macrocyclic Skeleton in *trans*-*twi*-**CP3a**

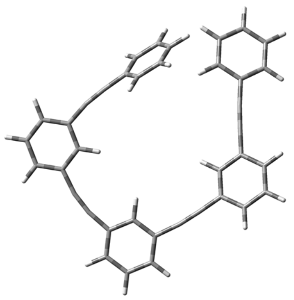

```
#p opt rwb97xd/6-31g(d)
```

Charge = 0, Multiplicity = 1

Number of Imaginary Frequencies = 0

Electronic energies = -1460.558439 Hartree

	Cartesian Coordinates				Cartesian Coordinates		
**Atom**	***X***	***Y***	***Z***	**Atom**	***X***	***Y***	***Z***
C	-3.375074	-3.798815	0.162508	H	2.792285	-1.843750	0.097186
C	-1.407693	-3.274431	2.083198	H	3.364293	-6.083734	-0.201233
C	-2.036332	-4.070959	-0.113166	H	5.761246	-5.711334	-0.703361
C	-3.718810	-3.252717	1.403003	H	6.682842	-3.413623	-0.810332
C	-2.740443	-2.998655	2.358635	C	-2.532103	3.428741	0.275733
C	-1.044623	-3.807317	0.838129	C	-0.754442	5.528898	0.803720
H	-1.754310	-4.463584	-1.084555	C	-1.154717	3.240849	0.124228
H	-4.761320	-3.019893	1.604308	C	-3.010259	4.678205	0.698734
H	-3.017145	-2.572497	3.318024	C	-2.122842	5.714820	0.959737
H	-0.636152	-3.066992	2.817543	C	-0.260500	4.286622	0.383836
C	-5.870838	-1.752181	-1.199023	H	-0.776006	2.278830	-0.203427
C	-6.539796	0.963149	-0.937122	H	-4.078931	4.824217	0.816815
C	-7.164353	-1.297943	-1.480906	H	-2.501716	6.678237	1.286444
C	-4.910277	-0.825805	-0.782275	H	-0.059962	6.337827	1.005078
C	-5.234436	0.531621	-0.661717	C	5.218134	-1.107529	-0.409837
C	-7.496723	0.045215	-1.345007	C	5.337789	0.097272	-0.402623
H	-7.914812	-2.003912	-1.824935	C	1.143538	4.066329	0.217605
H	-3.897940	-1.151138	-0.573375	C	2.321375	3.832366	0.065969
H	-8.506143	0.377813	-1.567522	C	3.672885	3.396358	-0.111007
H	-6.785397	2.015371	-0.838037	C	6.277938	2.415862	-0.457876
C	-4.245734	1.499236	-0.290402	C	3.911457	2.022042	-0.182428
C	-3.449799	2.370390	-0.019345	C	4.754053	4.281998	-0.214876
C	0.339437	-4.010140	0.528500	C	6.041989	3.785624	-0.387711
C	1.519737	-4.109022	0.278774	C	5.206593	1.521227	-0.353739
C	2.916714	-3.984245	-0.017312	H	3.079437	1.331296	-0.101700

	Cartesian Coordinates				Cartesian Coordinates		
**Atom**	***X***	***Y***	***Z***	**Atom**	***X***	***Y***	***Z***
C	5.632788	-3.572751	-0.588296	H	4.578253	5.351298	-0.160749
C	3.443927	-2.692583	-0.077583	H	6.875158	4.476859	-0.468334
C	3.763611	-5.075851	-0.246172	H	7.285369	2.035678	-0.590919
C	5.109636	-4.860981	-0.527696	H	-5.619910	-2.798797	-1.318966
C	4.795688	-2.473997	-0.362961	H	-4.134115	-4.023569	-0.577347

Optimized Macrocyclic Skeleton in **3b**

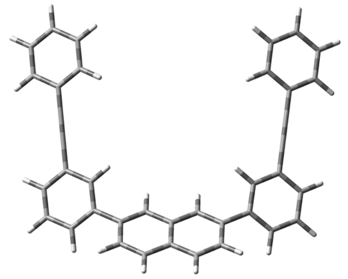

```
#p opt rwb97xd/6-31g(d)
```

Charge = 0, Multiplicity = 1

Number of Imaginary Frequencies = 0

Electronic energies = -1461.916613 Hartree

	Cartesian Coordinates				Cartesian Coordinates		
**Atom**	***X***	***Y***	***Z***	**Atom**	***X***	***Y***	***Z***
H	7.559727	3.391575	-0.741056	H	3.367271	-4.987515	1.151691
C	6.746765	3.991694	-0.345160	H	-1.249415	-6.076605	1.750763
C	4.647244	5.513672	0.673206	H	1.247167	-6.076961	1.750718
C	6.890076	5.365596	-0.198390	C	-2.439152	-3.291460	0.169578
C	5.546351	3.364452	0.017821	H	-1.229627	-1.769169	-0.718579
C	4.496268	4.140306	0.529303	C	-2.425171	-4.530615	0.867592
C	5.842604	6.129892	0.310406	H	-3.369229	-4.986558	1.151807
H	7.823570	5.841998	-0.482410	C	-1.245101	-5.134484	1.208628
H	3.567222	3.655008	0.810672	C	-3.729943	-2.653171	-0.192301
H	5.957668	7.203601	0.424007	C	-6.165410	-1.443573	-0.891517
H	3.828264	6.105738	1.070391	C	-4.796983	-3.418639	-0.676192
C	5.394352	1.950109	-0.131642	C	-3.901207	-1.274578	-0.058715
C	5.264822	0.753160	-0.257694	C	-5.110006	-0.659387	-0.406440
C	5.109229	-0.660834	-0.406563	C	-6.001986	-2.816344	-1.021969
C	4.795397	-3.419989	-0.676373	H	-4.672832	-4.490154	-0.804576
C	6.164383	-1.445312	-0.891713	H	-3.096074	-0.665841	0.340917
C	3.900267	-1.275684	-0.058801	H	-6.817948	-3.422459	-1.403651
C	3.728600	-2.654224	-0.192417	H	-7.103351	-0.968584	-1.159642
C	6.000557	-2.818032	-1.022193	C	-5.265190	0.754655	-0.257599
H	7.102448	-0.970585	-1.159868	C	-5.394097	1.951680	-0.131624
H	3.095326	-0.666726	0.340880	C	-5.544966	3.366158	0.017698
H	6.816328	-3.424372	-1.403928	C	-5.839014	6.131859	0.310037
H	4.670930	-4.491464	-0.804775	C	-4.493885	4.141368	0.528112
C	2.437641	-3.292150	0.169501	C	-6.745258	3.994176	-0.344337
H	1.228519	-1.769514	-0.718613	C	-6.887472	5.368206	-0.197692

Atom	Cartesian Coordinates			Atom	Cartesian Coordinates		
	X	*Y*	*Z*		*X*	*Y*	*Z*
C	1.238157	-2.703729	-0.162186	C	-4.643767	5.514866	0.671894
C	1.243102	-5.134838	1.208586	H	-3.564933	3.655467	0.808748
C	-0.000757	-3.302000	0.180027	H	-7.558994	3.394557	-0.739399
C	2.423332	-4.531304	0.867510	H	-7.820883	5.845212	-0.480972
C	-0.000921	-4.541246	0.874617	H	-3.824020	6.106430	1.068245
C	-1.239512	-2.703380	-0.162149	H	-5.953224	7.205670	0.423540

Strained Macrocyclic Skeleton in **AZ3b**

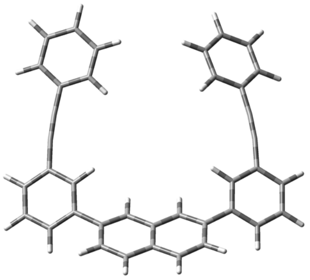

`#p opt rwb97xd/6-31g(d)`

Charge = 0, Multiplicity = 1

Number of Imaginary Frequencies = 0

Electronic energies = -1461.912331 Hartree

	Cartesian Coordinates				Cartesian Coordinates		
**Atom**	***X***	***Y***	***Z***	**Atom**	***X***	***Y***	***Z***
C	1.804918	5.370704	0.417842	H	2.939047	-0.384537	0.374390
C	4.393126	5.051351	-0.617983	H	7.383188	-2.191432	-0.797590
C	2.477153	6.450275	-0.159096	H	5.494309	-3.736489	-0.441166
C	2.442440	4.132246	0.475241	C	-4.713540	-1.177396	-0.464009
C	3.727181	3.956243	-0.052583	C	-4.322623	-3.921534	-0.810006
C	3.765175	6.289242	-0.663294	C	-3.479872	-1.767942	-0.166974
H	1.994750	7.419277	-0.231673	C	-5.760951	-1.980538	-0.930990
H	1.927791	3.286877	0.918495	C	-5.557456	-3.345944	-1.095846
H	4.278631	7.137292	-1.106077	C	-3.263251	-3.134549	-0.345321
H	5.389826	4.920263	-1.026444	H	-2.687086	-1.141474	0.228700
C	-3.233957	4.705598	0.705823	H	-6.722054	-1.532048	-1.160024
C	-5.792489	3.696909	0.142631	H	-6.367921	-3.968708	-1.462344
C	-3.418375	3.348879	0.442751	H	-4.173191	-4.985491	-0.971411
C	-4.346461	5.548952	0.699538	C	-1.924931	-3.701807	-0.043127
C	-5.613864	5.046590	0.414180	H	-0.872425	-2.029944	-0.859802
C	-4.689124	2.833599	0.161222	C	-0.790706	-2.982998	-0.343532
H	-2.565222	2.681011	0.459477	C	-0.543189	-5.448418	0.932526
H	-4.233523	6.605453	0.923641	C	0.497855	-3.444207	0.015263
H	-6.469702	5.714739	0.412916	C	-1.780408	-4.965477	0.593604
H	-6.779370	3.301210	-0.074185	C	0.631473	-4.695919	0.670530
C	4.296695	2.643648	-0.055336	C	1.651855	-2.663966	-0.230830
C	4.680403	1.495703	-0.083429	H	-2.670119	-5.536805	0.843091
C	-4.821328	1.430841	-0.087563	H	2.043068	-6.073436	1.564095
C	-4.843592	0.235193	-0.276064	H	-0.451792	-6.408026	1.434865
C	4.959767	0.093419	-0.144905	C	2.900364	-3.065980	0.186153

Atom	Cartesian Coordinates			Atom	Cartesian Coordinates		
	X	*Y*	*Z*		*X*	*Y*	*Z*
C	5.341324	-2.666352	-0.333581	H	1.531725	-1.721362	-0.758277
C	6.223809	-0.417579	-0.462273	C	3.026482	-4.327311	0.831546
C	3.903646	-0.793462	0.092366	H	4.004467	-4.650652	1.176669
C	4.070347	-2.174376	-0.016175	C	1.930219	-5.118510	1.057201
C	6.404649	-1.793472	-0.546421	H	-2.243819	5.092385	0.923870
H	7.049831	0.262262	-0.643278	H	0.803013	5.489725	0.817588

Strained Macrocyclic Skeleton in S-**DR3b**

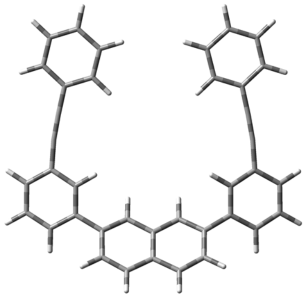

`#p opt rwb97xd/6-31g(d)`

Charge = 0, Multiplicity = 1

Number of Imaginary Frequencies = 0

Electronic energies = -1461.910376 Hartree

	Cartesian Coordinates				Cartesian Coordinates		
**Atom**	***X***	***Y***	***Z***	**Atom**	***X***	***Y***	***Z***
C	2.554815	5.207959	0.274284	H	2.787219	-0.811140	0.274350
C	5.248962	4.400934	-0.016187	H	6.960202	-3.112949	-1.022100
C	3.609087	6.153897	0.226717	H	4.909803	-4.430445	-0.636253
C	2.897252	3.839273	0.169957	C	-4.837819	-0.556088	-0.309617
C	4.226250	3.438581	0.026440	C	-4.890044	-3.338058	-0.559187
C	4.927424	5.750927	0.083725	C	-3.692520	-1.319046	-0.058699
H	3.384543	7.213637	0.294256	C	-6.023499	-1.204165	-0.676894
H	2.115622	3.090753	0.204959	C	-6.040196	-2.589440	-0.793512
H	5.716405	6.496125	0.046997	C	-3.695968	-2.707970	-0.192685
H	6.279993	4.082129	-0.127451	H	-2.790803	-0.807380	0.260988
C	-2.542529	5.209807	0.301719	H	-6.918079	-0.621162	-0.869735
C	-5.242454	4.407220	0.052117	H	-6.957793	-3.092200	-1.083850
C	-2.886745	3.843514	0.169059	H	-4.914037	-4.416791	-0.686122
C	-3.598556	6.155430	0.303173	C	-2.438529	-3.458686	0.049517
C	-4.919399	5.754599	0.179378	H	-1.207976	-1.960480	-0.852266
C	-4.218372	3.445158	0.047176	C	-1.233258	-2.908400	-0.321318
H	-2.103759	3.095573	0.166358	C	-1.260396	-5.368484	0.986853
H	-3.374663	7.213707	0.392143	C	-0.003992	-3.534687	-0.011081
H	-5.709476	6.499562	0.180109	C	-2.435755	-4.723244	0.700871
H	-6.275622	4.090162	-0.043004	C	-0.008298	-4.785278	0.659186
C	-4.523779	2.052736	-0.080509	C	1.229280	-2.911006	-0.310496
C	-4.737968	0.866232	-0.189108	H	-3.380911	-5.164429	1.004741
C	4.529588	2.043821	-0.078396	H	1.248752	-6.328142	1.513198
C	4.741614	0.855615	-0.171738	H	-1.276058	-6.326392	1.500493
C	4.839485	-0.567790	-0.280240	C	2.430115	-3.463385	0.071519

Atom	Cartesian Coordinates			Atom	Cartesian Coordinates		
	X	*Y*	*Z*		*X*	*Y*	*Z*
C	4.887296	-3.351082	-0.514584	H	1.210367	-1.963689	-0.842727
C	6.026850	-1.220569	-0.633576	C	2.418772	-4.727391	0.723916
C	3.690385	-1.326792	-0.034611	H	3.360116	-5.170196	1.037065
C	3.691513	-2.716394	-0.161718	C	1.239627	-5.370434	0.999029
C	6.041327	-2.606464	-0.742461	H	1.521940	5.519615	0.385294
H	6.924326	-0.640631	-0.822164	H	-1.507524	5.519667	0.396620

Strained Macrocyclic Skeleton in *cis*-*par*-**CP3b**

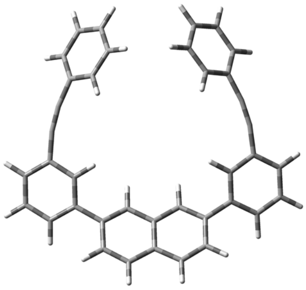

```
#p opt rwb97xd/6-31g(d)
```

Charge = 0, Multiplicity = 1

Number of Imaginary Frequencies = 0

Electronic energies = -1461.905517 Hartree

Atom	Cartesian Coordinates X	Y	Z	Atom	Cartesian Coordinates X	Y	Z
C	1.755632	4.751783	1.102629	H	2.811934	-0.630901	0.310145
C	3.869384	4.613432	-0.743903	H	7.050153	-2.730196	-1.074683
C	2.020030	5.817546	0.237752	H	5.061760	-4.135504	-0.704564
C	2.580976	3.631157	1.044146	C	-4.812412	-0.595474	-0.269012
C	3.612597	3.531281	0.103439	C	-4.789385	-3.371316	-0.567618
C	3.077164	5.751793	-0.663044	C	-3.651247	-1.336644	-0.027472
H	1.390210	6.702196	0.265086	C	-5.978124	-1.265671	-0.650070
H	2.390358	2.795720	1.707370	C	-5.956741	-2.648108	-0.788184
H	3.271868	6.588939	-1.326193	C	-3.611070	-2.719913	-0.190054
H	4.668527	4.546714	-1.474227	H	-2.764249	-0.807876	0.301148
C	-2.050128	4.620383	1.122827	H	-6.887214	-0.704486	-0.836784
C	-4.161972	4.368880	-0.719910	H	-6.860377	-3.169794	-1.087650
C	-2.802886	3.450667	1.049247	H	-4.787625	-4.446763	-0.715931
C	-2.387839	5.676140	0.269827	C	-2.327963	-3.435424	0.025032
C	-3.442893	5.553967	-0.627412	H	-1.147180	-1.850973	-0.785903
C	-3.830979	3.295443	0.112138	C	-1.141306	-2.825611	-0.307847
H	-2.556635	2.616576	1.694819	C	-1.081634	-5.349744	0.856833
H	-1.815679	6.598771	0.303553	C	0.109358	-3.423975	-0.033940
H	-3.693121	6.385233	-1.279230	C	-2.278157	-4.732889	0.604807
H	-4.959060	4.260366	-1.447392	C	0.149175	-4.709051	0.563063
C	-4.408785	1.992807	-0.029032	C	1.320322	-2.747547	-0.304434
C	-4.712233	0.826983	-0.135021	H	-3.205775	-5.225376	0.879537
C	4.273155	2.267559	-0.028305	H	1.460257	-6.254706	1.318817
C	4.651890	1.123620	-0.129838	H	-1.063504	-6.334815	1.314931
C	4.842410	-0.289871	-0.260263	C	2.542265	-3.280958	0.031892

	Cartesian Coordinates				Cartesian Coordinates		
**Atom**	***X***	***Y***	***Z***	**Atom**	***X***	***Y***	***Z***
C	4.995445	-3.062010	-0.556792	H	1.266013	-1.774346	-0.782308
C	6.048450	-0.885430	-0.639776	C	2.572896	-4.578962	0.611812
C	3.730350	-1.102851	-0.018634	H	3.529084	-5.011554	0.889361
C	3.778084	-2.485920	-0.180762	C	1.417224	-5.270497	0.860534
C	6.114942	-2.266567	-0.776611	H	0.949891	4.803018	1.825293
H	6.920324	-0.267934	-0.826259	H	-1.245937	4.713649	1.842798

Strained Macrocyclic Skeleton in *cis*-*twi*-**CP3b**

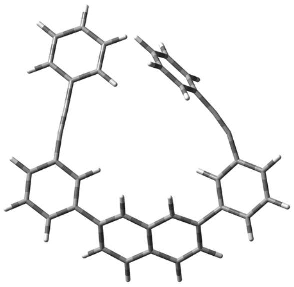

`#p opt rwb97xd/6-31g(d)`

Charge = 0, Multiplicity = 1

Number of Imaginary Frequencies = 0

Electronic energies = -1461.902802 Hartree

Atom	Cartesian Coordinates X	Y	Z	Atom	Cartesian Coordinates X	Y	Z
C	5.392981	-1.909247	-1.344395	H	-6.751276	-1.457135	-0.084203
C	6.386526	0.611825	-0.588898	C	2.478202	3.335640	0.553222
C	4.539569	-0.949405	-0.788787	C	0.575198	5.348174	0.964113
C	6.748960	-1.590872	-1.491203	C	1.123769	3.108346	0.291438
C	7.241836	-0.348946	-1.106873	C	2.874695	4.592565	1.028999
C	5.023485	0.319593	-0.439599	C	1.922464	5.586772	1.225621
H	3.486538	-1.171938	-0.662227	C	0.159316	4.096563	0.501919
H	7.425930	-2.319242	-1.928011	H	0.827792	2.144657	-0.109632
H	8.297262	-0.125321	-1.230129	H	3.923099	4.783698	1.233328
H	6.754690	1.594075	-0.311616	H	2.233206	6.559606	1.594607
C	2.759750	-4.119162	-0.307211	H	-0.158979	6.129274	1.141745
C	0.926596	-4.177492	1.808220	C	-1.257570	3.756912	0.216553
C	1.393414	-4.227614	-0.565525	H	-1.091320	1.844666	1.152857
C	3.196063	-4.019296	1.016688	C	-1.721398	2.513634	0.572594
C	2.285438	-4.055635	2.067123	C	-3.371807	4.203458	-0.903432
C	0.468188	-4.251468	0.484955	C	-2.977228	2.039801	0.133551
H	1.040667	-4.271901	-1.590424	C	-2.127637	4.623558	-0.502176
H	4.258594	-3.909691	1.218150	C	-3.817135	2.879921	-0.639483
H	0.207356	-4.192874	2.620602	C	-3.342225	0.694444	0.344912
C	4.132468	1.352883	-0.001690	H	-1.778083	5.615406	-0.775746
C	3.412923	2.283521	0.287639	H	-5.679005	2.956043	-1.746094
C	-0.942374	-4.233275	0.227055	H	-4.009632	4.869296	-1.479177
C	-2.135594	-4.085259	0.073470	C	-4.443318	0.136288	-0.262735
C	-3.436539	-3.486223	-0.046470	H	-2.688953	0.080959	0.958152
C	-5.819288	-2.015603	-0.101464	C	-5.309130	0.990687	-0.999405

Atom	Cartesian Coordinates			Atom	Cartesian Coordinates		
	X	*Y*	*Z*		*X*	*Y*	*Z*
C	-3.429885	-2.098600	-0.207700	H	-6.192412	0.571469	-1.473614
C	-4.663683	-4.147371	0.053960	C	-5.016234	2.322976	-1.161617
C	-5.842321	-3.404929	0.009839	H	2.635428	-3.979209	3.092045
C	-4.599023	-1.339188	-0.197588	H	-6.796520	-3.917023	0.090317
H	-2.470864	-1.610355	-0.334277	H	5.018088	-2.876178	-1.655680
H	-4.694336	-5.225332	0.174068	H	3.466024	-4.120812	-1.128750

Strained Macrocyclic Skeleton in *trans*-*par*-**CP3b**

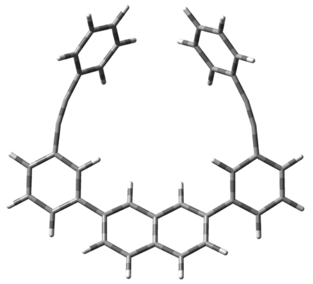

`#p opt rwb97xd/6-31g(d)`

Charge = 0, Multiplicity = 1

Number of Imaginary Frequencies = 0

Electronic energies = -1461.903339 Hartree

	Cartesian Coordinates				Cartesian Coordinates		
**Atom**	***X***	***Y***	***Z***	**Atom**	***X***	***Y***	***Z***
C	0.320155	5.133481	-0.852408	H	6.025359	-2.599620	-0.572111
C	2.372584	5.262320	1.065683	C	-4.470162	-1.858535	-0.247287
C	1.459648	4.339964	-0.966678	C	-3.714072	-4.537789	-0.455754
C	0.229364	6.007338	0.235648	C	-5.427178	-2.831691	-0.545503
C	1.258272	6.084984	1.168927	C	-3.143942	-2.251202	-0.042150
C	2.469027	4.360304	0.001767	C	-2.740430	-3.580208	-0.154797
H	1.537807	3.634118	-1.785345	C	-5.040040	-4.162809	-0.642818
H	-0.660725	6.616610	0.359207	H	-6.460384	-2.542370	-0.703553
H	3.150958	5.287893	1.820602	H	-2.421212	-1.490038	0.226646
C	-3.224126	4.044001	-0.838052	H	-5.780988	-4.919410	-0.881462
C	-5.012366	2.960514	1.039198	H	-3.430563	-5.579755	-0.567327
C	-3.677094	2.731363	-0.956030	C	3.337510	-2.458606	0.049486
C	-3.697446	4.817830	0.225658	H	1.710410	-1.381508	-0.821458
C	-4.599869	4.283185	1.139315	C	2.021918	-2.289516	-0.314374
C	-4.533115	2.164693	-0.005476	C	2.786078	-4.657775	0.926952
H	-3.309502	2.113033	-1.766627	C	1.036253	-3.261700	-0.027976
H	-3.355320	5.841100	0.336722	C	3.712551	-3.683799	0.666403
H	-4.963930	4.897681	1.956928	C	1.416945	-4.469580	0.609165
H	-5.678255	2.531101	1.780000	C	-0.328652	-3.029619	-0.314235
C	3.477147	3.342956	-0.063360	H	0.683258	-6.337713	1.415432
C	4.166551	2.353888	-0.155987	H	3.088998	-5.580322	1.414481
C	-4.760014	0.750481	-0.069662	C	-1.309843	-3.921815	0.050441
C	-4.751104	-0.455285	-0.159271	H	-0.594079	-2.107431	-0.821933
C	4.743299	1.044075	-0.246085	C	-0.914607	-5.140470	0.667857
C	5.660064	-1.583609	-0.459087	C	0.402813	-5.408141	0.927801

	Cartesian Coordinates				Cartesian Coordinates		
Atom	*X*	*Y*	*Z*	Atom	*X*	*Y*	*Z*
C	3.882304	-0.038358	-0.040732	H	1.175323	6.773086	2.004449
C	6.084951	0.796742	-0.546732	H	7.571629	-0.710340	-0.886082
C	6.531011	-0.515709	-0.646068	H	-1.676750	-5.853069	0.967162
C	4.313610	-1.358399	-0.155826	H	4.745655	-3.831231	0.965126
H	2.854250	0.170097	0.230608	H	-2.544253	4.457098	-1.573413
H	6.765311	1.626484	-0.704412	H	-0.460968	5.083497	-1.601340

Strained Macrocyclic Skeleton in *trans*-*twi*-**CP3b**

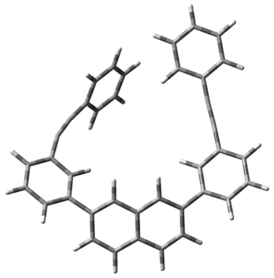

`#p opt rwb97xd/6-31g(d)`

Charge = 0, Multiplicity = 1

Number of Imaginary Frequencies = 0

Electronic energies = -1461.901928 Hartree

Atom	Cartesian Coordinates X	Y	Z	Atom	Cartesian Coordinates X	Y	Z
C	2.806617	-4.080020	0.197570	H	0.809593	2.022592	0.217299
C	0.707908	-3.672867	2.001169	H	-0.139022	6.199037	-0.109041
C	1.488289	-4.299761	-0.201310	C	-3.382209	-3.501540	-0.374853
C	3.063080	-3.638331	1.498707	C	-5.719420	-2.077389	-0.981946
C	2.018786	-3.445349	2.397201	C	-3.453901	-2.115857	-0.209782
C	0.430291	-4.090632	0.690600	C	-4.510502	-4.182358	-0.839329
H	1.277023	-4.605265	-1.220957	C	-5.669670	-3.463206	-1.127445
H	4.091230	-3.448318	1.795895	C	-4.593826	-1.381493	-0.531241
H	2.227067	-3.104804	3.406840	H	-2.584804	-1.606789	0.190671
H	-0.114710	-3.507542	2.689360	H	-4.482633	-5.259017	-0.971520
C	5.580203	-2.097722	-0.817259	H	-6.625100	-1.536734	-1.243185
C	6.458826	0.546746	-0.451838	C	-5.482134	0.879436	0.282917
C	6.932787	-1.760303	-0.949191	H	-5.981786	2.763144	1.155181
C	4.668038	-1.089536	-0.488542	C	-5.225720	2.183136	0.632102
C	5.096872	0.236528	-0.330671	C	-3.305016	0.691230	-0.716024
C	7.368989	-0.454788	-0.755489	C	-3.952648	2.768535	0.394323
H	7.650049	-2.526456	-1.228138	C	-4.491185	0.090634	-0.362212
H	3.613542	-1.323448	-0.396155	C	-2.990731	2.006561	-0.315024
H	8.423023	-0.215781	-0.860117	C	-3.556085	4.039078	0.892011
H	6.784316	1.573674	-0.322541	H	-2.547357	0.121510	-1.246906
C	4.161136	1.300923	-0.118705	C	-2.261281	4.475784	0.763250
C	3.417269	2.252239	-0.022307	H	-4.281053	4.643697	1.430989
C	-0.936398	-4.166808	0.264211	C	-1.282823	3.692882	0.087431
C	-2.103891	-4.071542	-0.048312	C	-1.682974	2.511014	-0.489137
C	2.482402	3.336864	0.011249	H	-0.973878	1.908805	-1.050169

Atom	Cartesian Coordinates			Atom	Cartesian Coordinates		
	X	***Y***	***Z***		***X***	***Y***	***Z***
C	0.588355	5.394118	-0.051342	H	2.271436	6.721149	-0.187884
C	2.897251	4.672193	-0.085049	H	-6.546815	-3.992485	-1.487637
C	1.115670	3.056328	0.098390	H	-6.432828	0.424552	0.547685
C	0.153428	4.068773	0.046067	H	-1.965338	5.416581	1.219022
C	1.948443	5.687570	-0.107473	H	5.251000	-3.116824	-0.977134
H	3.955757	4.902416	-0.147793	H	3.615915	-4.260082	-0.500077

Benzene

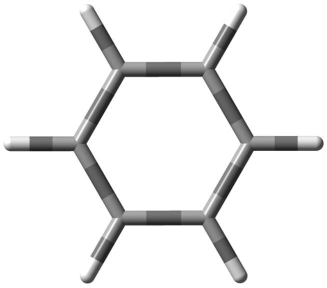

`#p opt rwb97xd/6-31g(d)`

Charge = 0, Multiplicity = 1

Number of Imaginary Frequencies = 0

Electronic energies = -232.164448 Hartree

Atom	Cartesian Coordinates			Atom	Cartesian Coordinates		
	X	***Y***	***Z***		***X***	***Y***	***Z***
C	0.466676	-1.312280	0.000001	H	0.830706	-2.335686	0.000002
C	1.369853	-0.251939	-0.000018	H	2.438150	-0.448446	-0.000007
C	0.903141	1.060284	0.000016	H	1.607350	1.887312	0.000020
C	-0.466730	1.312260	-0.000003	H	-0.830632	2.335713	0.000007
C	-1.369843	0.251995	-0.000014	H	-2.438164	0.448372	-0.000014
C	-0.903098	-1.060321	0.000013	H	-1.607408	-1.887262	0.000025

11. References

[S1] R. Izask and F. Neese, *J. Chem. Phys.*, 2011, **135**, 144105.

[S2] F. Neese, F. Wennmohs, A. Hansen and U. Becker, *Chem. Phys.*, 2009, **356**, 98–109.

[S3] F. Weigend, *J. Comput. Chem.*, 2008, **29**, 167–175.

[S4] A. Schäfer, H. Horn and R. Ahlrichs, *J. Chem. Phys.*, 1992, **97**, 2571–2577.
